# Supplementary material for: Characterization of PYL gene family and identification of HaPYL genes response to drought and salt stress in sunflower
Source: PeerJ. 2024 Mar 7;12:e16831. doi: 10.7717/peerj.16831 (PMC10924776; doi:10.7717/peerj.16831)
Supplement: Supplemental Information 1 [file peerj-12-16831-s001.docx]

**Additional File 1. PYL protein sequences of Helianthus annuus**

**Additional File 2. PYL protein sequences of Arabidopsis thaliana(At), Oryza sativa(Os), Zea mays(Zm), and Nicotiana tabacum(Nt).**

**Additional File 3. The 2000 bp sequences upstream of HaPYL genes.**

**Additional File 4. mRNA sequences of HaPYLs for qRT-PCR**

**Additional File 5. List of primers used for qRT-PCR validation.**

**Additional File 6. Gene Ontology enrichment of HaPYLs**

**Additional File 7. Protein interaction network of the HaPYL protein with other sunflower proteins predicted by the STRING online service.**

**Additional file 8. Protein annotation of HaPYLs interacting proteins predicted by the STRING online service.**

**Additional File 1. PYL protein sequence of *Helianthus annuus***

>transcript:mRNA:HanXRQr2_Chr03g0121241

MEPKSIPQGLTADEYSELQLLINAYHLFDKIPNTCTSLITQRIDAPTNIVWPLVRRFDNPQRYKHFIKSCSMFGDGGVGSIREVTVISGLPASTSTERLELLDDEKHILSFRVVGGEHRLNNYLSVTSVNEFEKDGKVYTVVLESYIVDIPTGNTVEDTKMFTDTVVKLNLQKLGLVAMACLHGSE

>transcript:mRNA:HanXRQr2_Chr04g0182281

MDAAHYIRRHHRHTLGHKQCASVVVRHIKAPVDIVWSLVRRFDEPQKYKPFVSRCTMHGEINIGSVREVNVKSGLPATTSTERLELLDDKEHILGIKIVGGDHRLKNYSSILTVHPEVSEGSGTLVVESFVVDIPDGNTMEETCYFVRALINCNLKCLSDVSERMAICPDSGMKRSEMAHGVPV

>transcript:mRNA:HanXRQr2_Chr06g0241551

MALTGDDDATDSSENSYIRKHHKHEVRDNQCSSSLVKHIKAPVHLVWSLVRRFDQPQKYKPFVSGCNVQGDLEIGSVREVCVRSGLPATTSMERLELLDEEEHILGIKIVGGDHRLRNYSSIITLHPEILDGRPGTLVIESFVVDVPDGNTKDETCYFVEALIKCNLKSLADVSERLSVQDRTESIVGA

>transcript:mRNA:HanXRQr2_Chr06g0242911

MEQAGTSTPEHQNPPPQTTTTTHHLSLPPGLTEHEFNQLKTFVSNFHTYHLSPSQCSSLLAQHIHAPVDVVWSVVRRFDKPQTYKHFIKSCTVGENFKMEVGCTRDVNVISGLPAATSTERLDLLDDDNHVMAFTIIGGEHRLRNYHAVTTVHEVVTENDDSVTVVLESYVVDVPEGNTEEDTRLFADTVVKLNLQKLAAVTEAMAVDGGASIKNLNLR

>transcript:mRNA:HanXRQr2_Chr06g0246931

MDAAQYIRRHHRHNPGRQQCASAVVKHIKAPVDIVWSLVRRFDQPQKYKPFVSRCTMRGGLNIGSVREVDVKSGLPATTSTERLELLDDTEHILGIKIVGGDHRLKNYSSILTVHPEVIEGRSGTLVIESFVVDIPNGNTQDETCYFVKALINCNLKSLSDISERMAVQTQM

>transcript:mRNA:HanXRQr2_Chr07g0285411

MDRNTNSNSNSIPQGLTQEEYTQLQPLINAYHMFDKMPNTCTSLITQRIEAPAHVVWALVRRFDNPQRYKHFIKSCSMIGDGGVGSIREVNVISGIPASTSTERLELVDEERHILSFRVVGGEHRLNNYLSVTSVNEFSKEEKVYTIVLESYVVDVPEGNTVDDTKMFTDTVVKLNLQKLGVVAMGCLHGGES

>transcript:mRNA:HanXRQr2_Chr07g0313141

MPSPIQIQRIHPTTTTPTTTTTVNHHKQPLPTTTIWRVPSSICIPEDLMHHHTHVVGPNRCSSVVVQTISAPVDIVWSVVRRFDNPQAYKHFLKSCHVILGDGDVGSLRKVHVVSGLPAGSSTERLEILDDERHVMSFSVVGGDHRLNNYRSVTTLHPSQCDGCTVVVESYVVDVPAENTKEETCVFVDTIVRCNMISLKQIAENLVKK

>transcript:mRNA:HanXRQr2_Chr07g0316871

MSSCRDNNNNISSLESEYIKKHHRHENIADNQCTSVLLKHIKAPVHLVWSLVRRFDEPQKYKPFVSRCVAQGNLEIGSLREVDVKSGLPATTSTERLELLDDDQHIFSIRIIGGDHRLRNYSSIISVHPEIIEGRPGTLVVESFVVDVPEGNTKDETCYFVQALIKCNLKSLADVSERLAVQDRTEPIDRM

>transcript:mRNA:HanXRQr2_Chr16g0742371

MNTNPIPQGLTQDEYSNLQPIITTHHLFTKTPNTCTSLITQRINAPAHIVWSLVRRFDNPQRYKHFIKSCTMTGDGGVGSIREVTVISGLPASTSTERLELVDDEKHILSFRVVGGEHRLNNYCSVTSVNEFSEGDKVYTIVLESYVVDVPQGNTVEDTKMFTDTVVKLNLHKLGAVAVGCLHGDG

>transcript:mRNA:HanXRQr2_Chr08g0334961

MCTSIQIQPHPKSTMNHQSSTIGTGIVCKQLPQLINIPEDLHRHHTHRVSHNQCFSTIIQPIAAPLETVWSLVRRFDNPQAYKHFLKSCDLIVGDGDVGSVREVQVVSGLPAASSMERLDILDDERHVISFSVVGGDHRLKNYRSVTTLHSSPTCGGGTVVVESYVVDVPPENTKEETCVFVETIVRCNLLSLKQLAQDLVHDK

>transcript:mRNA:HanXRQr2_Chr15g0671331

MTISGTGDGDSGGTSLTERDYIKRHHKHEVRACQCTSTVVKRIKAPVHLVWSLVRRFDEPQKYKPFVSGCNVQGDLEIGSVRQVNVCSGLPATTSIERLELLNEEEHILGMRIVGGDHRLQNYSSVITLHPDIIEGRPGTLVIESFVVDVPDGNTIDETCYFVEALIKCNLKSLANVSERLTVQDHTDPIVHV

>transcript:mRNA:HanXRQr2_Chr02g0070891

MLSNPRTTTHPFPDTISSLHTHPVGHHQCCSAVIQIINAPVSTVWAVVRRFDNPQAYKNFVKSCHVLNGSGDVGTLRQVHVISGLPAATSTERLDILDDERHVLSFSVIGGDHRLSNYRSVTTLHPTPTGTGTVVVESYVVDIPTGNTKEETVGFVDTIVKCNLQSLAKIADDKLS

>transcript:mRNA:HanXRQr2_Chr10g0429361

MLSNPQNSPSFLLDRINTTTVTGGSGACGNTILQPTTTIPIPDSVARHHIHPVSPNQCCSAVIQHINAPISDVWSVIRRFDNPQAYKHFVKSCHVINGDGNVGTLREVHVISGLPAARSTERLEILDDEQHVISFSVVDGDHRLANYRSVTTLHPTPAGDGTVVVESYVVDIPPGNTKEEACVFVDTIVKCNLQSLAQISENKLR

>transcript:mRNA:HanXRQr2_Chr12g0530941

METTHHLHPPPGVTEQEFDTLKSSIKEFHTYIHTRAQCSSLLAQHIHTSSDIVWSIIRQFDKPQVYKHFIKSCSVKEGSVIAEGCTRDVIVISGLPAATSTERLDRLDDENHVMEFSIIGGEHRLRNYRAVTTVHQIKPNETIVLESYMVDVPEGNSEEDTRFFADTVVRLNLQKLANVTEAIARGAETAAAAESLSIR

>transcript:mRNA:HanXRQr2_Chr13g0596431

MDQNSIPEGLTPEEYSQLQLLINAHHMFDKMPNTCTSLITQHIDAPARVVWPLVRSFDNPQRYKHFIKSCNMSGDGGVGSIREVTVMSGLPASTSTERLEVLDDEKHILSFRVLGGEHRLSNYLSVTSVNEFKKGEKVYTVVLESYIVDVPVGNTVEDTKMFTDTVVKMNLQKLGLVALSCLRGNE

>transcript:mRNA:HanXRQr2_Chr14g0666021

MPSSVQVHRIHPTTTTTTTTTTVNNHKQPSTTTIWRVPSSINIPEDLNHHHTHAVGPHQCSSAVVQTIAAPIAAVWSVVRRFDKPQVYKHFLKSCHVILGDGDVGTLREVHVVSGLPAGSSTERLEILDDDRHVMSFSVVGGDHRLNNYRSVTTLHASPTCDGSTVVVESYVVDVPPENTKEETCVFVDTIVRCNLISLKQIAESK

>transcript:mRNA:HanXRQr2_Chr08g0342731

MLSNPQKSPSFFLDGINTTTTTTTTGSSGSCGTNKPPPTNTTPVPDSVARYHIHAVGPDQCCSVVIQHITAPISDVWSIVRRFDNPQAYKHFVKTCHVILGDGNVGTLREIHVISGLPAARSTERLEILDDEQHVISFSVVDGDHRLANYRSVTTLHPTPDGTGTVVVESYVVDIPPGNTKEETCVFVDTIVKCNLQSLAQKIRRN

>transcript:mRNA:HanXRQr2_Chr05g0211711

MCSSIQIQRIQENPKSTVNHHTTTTVRRVPPSVYLPEHIHHHHIHTMTLNQCSSTIVQTISAPVETVWSVVRRFDEPHAYKHFLKSCDVIVGDGDVGSVREVRVVSGLPAGSSLERLEICDDERHVMSFSVVGGDHRLKNYRSVTTLHSSPTCRVTTVVVESYVVDVPPENTKEETCVFVETIVRCNLMSLKHIAQSLAQK

>transcript:mRNA:HanXRQr2_Chr17g0780891

MVMNGHDDATDSSEYSHIRRHHKHEVRANQCTSSLVKHIKAPVHLVWSLVRRFDQPQKYKPFVSGCYVQGNLGIGSVREVCVRSGLPATTSMERLELLDEEEHILGIKIVGGDHRLRNYSSIMTLHPEIIDGRPGTLVIESFVVDVPDGNTKDETCYFVEALIKCNLKSLADVSERLAVQDRTEPIVGV

**Additional File 2. PYL protein sequence of *Arabidopsis thaliana*(At), *Oryza sativa*(Os), *Zea mays*(Zm), *Nicotiana tabacum*(Nt)**

>AtPYR1

MPSELTPEERSELKNSIAEFHTYQLDPGSCSSLHAQRIHAPPELVWSIVRRFDKPQTYKHFIKSCSVEQNFEMRVGCTRDVIVISGLPANTSTERLDILDDERRVTGFSIIGGEHRLTNYKSVTTVHRFEKENRIWTVVLESYVVDMPEGNSEDDTRMFADTVVKLNLQKLATVAEAMARNSGDGSGSQVT

>AtPYL1

MANSESSSSPVNEEENSQRISTLHHQTMPSDLTQDEFTQLSQSIAEFHTYQLGNGRCSSLLAQRIHAPPETVWSVVRRFDRPQIYKHFIKSCNVSEDFEMRVGCTRDVNVISGLPANTSRERLDLLDDDRRVTGFSITGGEHRLRNYKSVTTVHRFEKEEEEERIWTVVLESYVVDVPEGNSEEDTRLFADTVIRLNLQKLASITEAMNR

NNNNNNSSQVR

>AtPYL2

MSSSPAVKGLTDEEQKTLEPVIKTYHQFEPDPTTCTSLITQRIHAPASVVWPLIRRFDNPERYKHFVKRCRLISGDGDVGSVREVTVISGLPASTSTERLEFVDDDHRVLSFRVVGGEHRLKNYKSVTSVNEFLNQDSGKVYTVVLESYTVDIPEGNTEEDTKMFVDTVVKLNLQKLGVAATSAPMHDDE

>AtPYL3

MNLAPIHDPSSSSTTTTSSSTPYGLTKDEFSTLDSIIRTHHTFPRSPNTCTSLIAHRVDAPAHAIWRFVRDFANPNKYKHFIKSCTIRVNGNGIKEIKVGTIREVSVVSGLPASTSVEILEVLDEEKRILSFRVLGGEHRLNNYRSVTSVNEFVVLEKDKKKRVYSVVLESYIVDIPQGNTEEDTRMFVDTVVKSNLQNLAVISTASPT

>AtPYL4

MLAVHRPSSAVSDGDSVQIPMMIASFQKRFPSLSRDSTAARFHTHEVGPNQCCSAVIQEISAPISTVWSVVRRFDNPQAYKHFLKSCSVIGGDGDNVGSLRQVHVVSGLPAASSTERLDILDDERHVISFSVVGGDHRLSNYRSVTTLHPSPISGTVVVESYVVDVPPGNTKEETCDFVDVIVRCNLQSLAKIAENTAAESKKKMSL

>AtPYL5

MRSPVQLQHGSDATNGFHTLQPHDQTDGPIKRVCLTRGMHVPEHVAMHHTHDVGPDQCCSSVVQMIHAPPESVWALVRRFDNPKVYKNFIRQCRIVQGDGLHVGDLREVMVVSGLPAVSSTERLEILDEERHVISFSVVGGDHRLKNYRSVTTLHASDDEGTVVVESYIVDVPPGNTEEETLSFVDTIVRCNLQSLARSTNRQ

>AtPYL6

MPTSIQFQRSSTAAEAANATVRNYPHHHQKQVQKVSLTRGMADVPEHVELSHTHVVGPSQCFSVVVQDVEAPVSTVWSILSRFEHPQAYKHFVKSCHVVIGDGREVGSVREVRVVSGLPAAFSLERLEIMDDDRHVISFSVVGGDHRLMNYKSVTTVHESEEDSDGKKRTRVVESYVVDVPAGNDKEETCSFADTIVRCNLQSLAKLAENTSKFS

>AtPYL7

MEMIGGDDTDTEMYGALVTAQSLRLRHLHHCRENQCTSVLVKYIQAPVHLVWSLVRRFDQPQKYKPFISRCTVNGDPEIGCLREVNVKSGLPATTSTERLEQLDDEEHILGINIIGGDHRLKNYSSILTVHPEMIDGRSGTMVMESFVVDVPQGNTKDDTCYFVESLIKCNLKSLACVSERLAAQDITNSIATFCNASNGYREKNHTETNL

>AtPYL8

MEANGIENLTNPNQEREFIRRHHKHELVDNQCSSTLVKHINAPVHIVWSLVRRFDQPQKYKPFISRCVVKGNMEIGTVREVDVKSGLPATRSTERLELLDDNEHILSIRIVGGDHRLKNYSSIISLHPETIEGRIGTLVIESFVVDVPEGNTKDETCYFVEALIKCNLKSLADISERLAVQDTTESRV

>AtPYL9

MMDGVEGGTAMYGGLETVQYVRTHHQHLCRENQCTSALVKHIKAPLHLVWSLVRRFDQPQKYKPFVSRCTVIGDPEIGSLREVNVKSGLPATTSTERLELLDDEEHILGIKIIGGDHRLKNYSSILTVHPEIIEGRAGTMVIESFVVDVPQGNTKDETCYFVEALIRCNLKSLADVSERLASQDITQ

>AtPYL10

MNGDETKKVESEYIKKHHRHELVESQCSSTLVKHIKAPLHLVWSIVRRFDEPQKYKPFISRCVVQGKKLEVGSVREVDLKSGLPATKSTEVLEILDDNEHILGIRIVGGDHRLKNYSSTISLHSETIDGKTGTLAIESFVVDVPEGNTKEETCFFVEALIQCNLNSLADVTERLQAESMEKKI

>AtPYL11

METSQKYHTCGSTLVQTIDAPLSLVWSILRRFDNPQAYKQFVKTCNLSSGDGGEGSVREVTVVSGLPAEFSRERLDELDDESHVMMISIIGGDHRLVNYRSKTMAFVAADTEEKTVVVESYVVDVPEGNSEEETTSFADTIVGFNLKSLAKLSERVAHLKL

>AtPYL12

MKTSQEQHVCGSTVVQTINAPLPLVWSILRRFDNPKTFKHFVKTCKLRSGDGGEGSVREVTVVSDLPASFSLERLDELDDESHVMVISIIGGDHRLVNYQSKTTVFVAAEEEKTVVVESYVVDVPEGNTEEETTLFADTIVGCNLRSLAKLSEKMMELT

>AtPYL13

MESSKQKRCRSSVVETIEAPLPLVWSILRSFDKPQAYQRFVKSCTMRSGGGGGKGGEGKGSVRDVTLVSGFPADFSTERLEELDDESHVMVVSIIGGNHRLVNYKSKTKVVASPEDMAKKTVVVESYVVDVPEGTSEEDTIFFVDNIIRYNLTSLAKLTKKMMK

>OsPYL1

MEQQEEVPPPPAGLGLTAEEYAQVRATVEAHHRYAVGPGQCSSLLAQRIHAPPAAVWAVVRRFDCPQVYKHFIRSCVLRPDPHHDDNGNDLRPGRLREVSVISGLPASTSTERLDLLDDAHRVFGFTITGGEHRLRNYRSVTTVSQLDEICTLVLESYIVDVPDGNTEDDTRLFADTVIRLNLQKLKSVSEANANAAAAAAAPPPPPPAAAE*

>OsPYL2

MEAHVERALREGLTEEERAALEPAVMAHHTFPPSTTTATTAAATCTSLVTQRVAAPVRAVWPIVRSFGNPQRYKHFVRTCALAAGDGASVGSVREVTVVSGLPASTSTERLEMLDDDRHIISFRVVGGQHRLRNYRSVTSVTEFQPPAAGPAPAPPYCVVVESYVVDVPDGNTAEDTRMFTDTVVKLNLQKLAAVAEDSSSASRRRD*

>OsPYL3

MEPHMERALREAVASEAERRELEGVVRAHHTFPAAERAAGPGRRPTCTSLVAQRVDAPLAAVWPIVRGFANPQRYKHFIKSCELAAGDGATVGSVREVAVVSGLPASTSTERLEILDDDRHVLSFRVVGGDHRLRNYRSVTSVTEFSSPSSPPSPPRPYCVVVESYVVDVPEGNTEEDTRMFTDTVVKLNLQKLAAVATSSSPPAAGNHH*

>OsPYL4

MPYAAVRPSPPPQLSRPIGSGAGGGKACPAVPCEVARYHEHAVGAGQCCSTVVQAIAAPADAVWSVVRRFDRPQAYKKFIKSCRLVDGDGGEVGSVREVRVVSGLPATSSRERLEVLDDDRRVLSFRIVGGEHRLANYRSVTTVHEAAAPAMAVVVESYVVDVPPGNTWEETRVFVDTIVRCNLQSLARTVERLAPEAPRANGSIDHA*

>OsPYL5

MMPYTAPRPSPPQHSRIGGCGGGGVLKAAGAAGHAASCVAVPAEVARHHEHAAGVGQCCSAVVQAIAAPVDAVWSVVRRFDRPQAYKHFIRSCRLLDGDGDGGAVAVGSVREVRVVSGLPATSSRERLEILDDERRVLSFRVVGGEHRLSNYRSVTTVHETAAGAAAAVVVESYVVDVPHGNTADETRMFVDTIVRCNLQSLARTAEQLALAAPRAA*

>OsPYL6

MPCIPASSPGIPHQHQHQHHRALAGVGMAVGCAAEAAVAAAGVAGTRCGAHDGEVPMEVARHHEHAEPGSGRCCSAVVQHVAAPAPAVWSVVRRFDQPQAYKRFVRSCALLAGDGGVGTLREVRVVSGLPAASSRERLEILDDESHVLSFRVVGGEHRLKNYLSVTTVHPSPSAPTAATVVVESYVVDVPPGNTPEDTRVFVDTIVKCNLQSLANTAEKLAAGARAAGS*

>OsPYL7

MNSGAGGAGGAAVGRMPAGSLQWAQWRLADERCELREEEMEYMRRFHRHEIGSNQCNSFIAKHVRAPLQNVWSLVRRFDQPQIYKPFVRKCVMRGNVETGSVREIIVQSGLPATRSIERLEFLDDNEYILRVKFIGGDHMLKKCGP

>OsPYL8

MNGAGGAGGAAAGKLPMVSHRQVQWRLADERCELREEEMEYIRQFHRHEPSSNQCTSFVAKHIKAPLQTVWSLVRRFDQPQLFKPFVRKCVMRENIIATGCVREVNVQSGLPATRSTERLELLDDNEHILKVKFIGGDHMLKNYSSILTIHSEVIDGQLGTLVVESFVVDIPEGNTKDDICYFIENILRCNLMTLADVSEERLANP*

>OsPYL9

MNGVGGAGGAAAGKLPMVSHRRVQWRLADERCELREEEMEYIRRFHRHEPSSNQCTSFAAKHIKAPLHTVWSLVRRFDQPQLFKPFVRNCVMRENIIATGCIREVNVQSGLPATRSTERLELLDDNEHILKVKFIGGDHMLKNYSSILTIHSEVIDGQLGTLVVESFIVDVLEGNTKDDISYFIENVLRCNLRTLADVSEERLANP*

>OsPYL10

MVEVGGGAAEAAAGRRWRLADERCDLRAAETEYVRRFHRHEPRDHQCSSAVAKHIKAPVHLVWSLVRRFDQPQLFKPFVSRCEMKGNIEIGSVREVNVKSGLPATRSTERLELLDDNEHILSVRFVGGDHRLKNYSSILTVHPEVIDGRPGTLVIESFVVDVPEGNTKDETCYFVEALLKCNLKSLAEVSERLVVKDQTEPLDR*

>OsPYL11

MVGLVGGGGWRVGDDAAGGGGGGAVAAGAAAAAEAEHMRRLHSHAPGEHQCSSALVKHIKAPVHLVWSLVRSFDQPQRYKPFVSRCVVRGGDLEIGSVREVNVKTGLPATTSTERLELLDDDEHILSVKFVGGDHRLRNYSSIITVHPESIDGRPGTLVIESFVVDVPDGNTKDETCYFVEAVIKCNLTSLAEVSERLAVQSPTSPLEQ*

>OsPYL12

MRGSTSLAVGCVREVDFKSGFPAKSSVERLEILDDKEHVFGVRIIGGDHRLKNYSSVLTAKPEVIDGEPATLVSESFVVDVPEGNTADETRHFVEFLIRCNLRSLAMVSQRLLLAQGDLAEPPAQ*

>OsPYL13

MNGCTGGAGGVAAGRLPAVSLQQAQWKLVDERCELREEEMEYVRWFHRYELVATGATPSLPNTSGCPSKLGLPSTRRIERLGFPDDNDHTLRVKFIGGDHMLKDYSSTLIIHLEVIDGQLVTLVIESFVVDILEGNTKDEISYFIENLLKFNLRTLRV*

>ZmPYL1

MDQQGAGGDVEVPAGLGLTAAEYEQLRPTVDAHHRYAVGEGQCSSLLAQRIHAPPAAVWAIVRRFDCPQVYKHFIRSCAVRPDPDAGDALRPGRLREVCVISGLPASTSTERLDHLDDAARVFGFSITGGEHRLRNYRSVTTVSELAGPGICTVVLESYAVDVPDGNTEDDTRLFADTVIRLNLQKLKSVAEASTSSSAPPPPSE

>ZmPYL2

MDQQGAGGDAEVPAGLGLTAAEYEQLRSTVDAHHRYAVGEGQCSSLLAQRIHAPPEAVWAVVRRFDCPQVYKHFIRSCALRPDPEAGDALCPGRLREVSVISGLPASTSTERLDLLDDAARVFGFSITGGEHRLRNYRSVTTVSELADPAICTVVLESYVVDVPDGNTEDDTRLFADTVIRLNLQKLKSVAEANAAEAAATTNSVLLPRPAE

>ZmPYL3

MEPHMESALRQGLSEAEQRELEGVVRAHHTFPGRAPGTCTSLVTQRVDAPLAAVWPIVRGFGSPQRYKHFIKSCDLKAGDGATVGSVREVTVVSGLPASTSTERLEILDDHRHILSFRVVGGDHRLRNYRSVTSVTEFQPGPYCVVLESYVVDVPDGNTEEDTRMFTDTVVKLNLQKLAAIATSSSAN

>ZmPYL4

MPYTAPRPSPQQHSRVLSGGGAKAASHGASCAAVPAEVARHHEHAARAGQCCSAVVQAIAAPVGAVWSVVRRFDRPQAYKHFIRSCRLVGGGDVAVGSVREVRVVSGLPATSSRERLEILDDERRVLSFRVVGGEHRLANYRSVTTVHEAGAGAGTGTVVVESYVVDVPHGNTADETRVFVDTIVRCNLQSLARTAERLA

>ZmPYL5

MPCLQASSPGSMPYQHHGRGVGCAAEAGAAVGASAGTGTRCGAHDGEVPAEAARHHEHAAPGPGRCCSAVVQRVAAPAEAVWSVVRRFDQPQAYKRFVRSCALLAGDGGVGTLREVRVVSGLPAASSRERLEVLDDESHVLSFRVVGGEHRLQNYLSVTTVHPSPAAPDAATVVVESYVVDVPPGNTPEDTRVFVDTIVKCNLQSLATTAEKLALAAV

>ZmPYL6

MVGLVGGSTARAEHVVANAGGEAEYVRRMHRHAPTEHQCTSTLVKHIKAPVHLVWELVRRFDQPQRYKPFVRNCVVRGDQLEVGSLRDVNVKTGLPATTSTERLEQLDDDLHILGVKFVGGDHRLQNYSSIITVHPESIDGRPGTLVIESFVVDVPDGNTKDETCYFVEAVIKCNLNSLAEVSEQLAVESPTSLIDQ

>ZmPYL7

MPYAATRTSPQQHSRVASNGRAVAACAGHAGVPDEVARHHEHAVAAGQCCSVMVQSIAAPADAVWSLVRRFDQPQGYKRFIRSCHLVDGDGVEVGSVRELLVVSGLPAENSRERLEIRDDERRVISFRILGGDHRLANYRSVTTVHEAASEGGPLTMVVESYVVDVPPGNTVEETRIFVDTIVRCNLQSLEDTVIRQQAMAAPAAPHNDHNHS

>ZmPYL8

MVVEMDGGVGVAAAGGGGAQTPAPPPPRRWRLADERCDLRAMETDYVRRFHRHEPRDHQCSSAVAKHIKAPVHLVWSLVRRFDQPQLFKPFVSRCEMKGNIEIGSVREVNVKSGLPATRSTERLELLDDDERILSVRFVGGDHRLQNYSSILTVHPEVIDGRPGTLVIESFVVDVPDGNTKDETCYFVEALLKCNLRSLAEVSEGQVIMDQTEPLDR

>ZmPYL9

MVGLVGGSTARAEHVVANAGGETEYVRRLHRHAPAEHQCTSTLVKHIKAPVHLVWELVRSFDQPQRYKPFVRNCVVRGDQLEVGSLRDVNVKTGLPATTSTERLEQLDDDLHILGVKFVGGDHRLQNYSSIITVHPESIDGRPGTLVIESFVVDVPDGNTKDETCYFVEAVIKCNLKSLAEVSEQLAVESPTSPIDQ

>ZmPYL10

MVMVEMDGGVGGGGGGGQTPAPRRWRLADERCDLRAMETDYVRRFHRHEPREHQCSSAVAKHIKAPVHLVWSLVRRFDQPQLFKPFVSRCEMKGNIEIGSVREVNVKSGLPATRSTERLELLDDNEHILSVRFVGGDHRLQNYSSILTVHPEVIDGRPGTLVIESFVVDVPDGNTKDETCYFVEALLKCNLKSLAEVSERQVVKDQTEPLDR

>ZmPYL11

MPCIQASSPGGMPHQHGRGRVLGGGVGCAAEVAAAVAASAGGMRCGAHDGEVPAEAARHHEHAAAGPGRCCSAVVQHVAAPAAAVWSVVRRFDQPQVYKRFVRSCALLAGDGGVGTLREVRVVSGLPAASSRERLEVLDDESHVLSFRVVGGEHRLRNYLSVTTVHPSPAAPDAATVVVESYVVDVPPGNTPEDTRVFVDTIVKCNLQSLATTAEKLAAV

>ZmPYL12

MVGLVGGSTARAEHVVANAGGEAEYVRRMHRHAPTEHQCTSTLVKHIKAPVHLVWELVRRFDQPQRYKPFVRNCVVRGDQLEVGSLRDVNVNPGLPATTSTERLEQLDDDLHILGVKFVGGDHRLQS

>ZmPYL13

MRERNSSIDQEHQRGSSSRSTMPFAASRTSQQQHSRVATNGRAVAVCAGHAGVPDEVARHHEHAVAAGQCCAAMVQSIAAPVDAVWSLVRRFDQPQRYKRFIRSCHLVDGDGAEVGSVRELLLVSGLPAESSRERLEIRDDERRVISFRVLGGDHRLANYRSVTTVHEAAPSQDGRPLTMVVESYVVDVPPGNTVEETRIFVDTIVRCNLQSLEGTVIRQLEIAAMPHDDNQN

>NtPYL1

MDGGQVPQGLTEEEFAELEALIQNYHTFDHLPNTCTSLITQRIDAPAHVVWPFVRRFDNPEKYKHFIKSCKMTTGEGEVGSIREVTVVSGIPASTSTERLEILDDEKHILSFRVVGGEHRLNNYRSVTSVNEFKKDGKVYAIVLESYIVDIPEGNTEEDTKMFTDTVVKLNLQKLGVVAMAALHGHEL

>NtPYL2

MDGADQVPQGLTEEEFAELEPLIRNYHTFDHLPNTCTSLITQHIDAPAHVVWPFVRRFDNPEKYKHFIKSCKMTAGDGEVGSIREVTVVSGIPASTSTERLEILDDEKHILSFRVVGGEHRLNNYRSVTSVNEFKKDGKIYTIVLESYIVDIPEGNTGEDTKMFTDTVVKLNLQKLGVVAMAALHGHEL

>NtPYL3

MSRSQVPHGLKEEEFIELEPLILNYHTFDHLPNTCTSLITQRIEAPANAVWPFVRRFDNPQKYKHFIKSCKMTGDGGVGSMREVSVVSGIPASTSTERLEILDDEKHILSFRVVGGEHRLNNYRSVTSVNEFKKNGKAYTIVLESYIVDIPEGNTGEDTKMFTDTVVKLNLQKLGVVAMAALHGHE

>NtPYL4

MSRSQVPHGLKEEEFIELEPLIHNYHTFDHLPNTCTSLITQRIEAPANVVWPFVRRFDNPQKYKHFIKSCKMTGDGGVGSMREVSVVSGIPASTSTERLEILDDEKHILSFRVVGGEHRLNNYRSVTSVNEFKKNGKAYTIVLESYIVDIPEGNTWEDTKMFTDTVVKLNLQKLGVVAMAALHGHE

>NtPYL5

MSRSQVPHGLKEEEFIELEPLIHNYHTFDHLPNTCTSLITQRIEAPANVVWPFVRRFDNPQKYKHFIKSCKMTGDGGVGSMREVSVVSGIPASTSTERLEILDDEKHILSFRVVGGEHRLNNYRSVTSVNEFKKNGKAYTIVLESYIVDIPEGNTWEDTKMFTDTVVKLNLQKLGVVAMAALHGHE

>NtPYL6

MPSSLQLRRINPSTATSTATLAGNYHKQQSKSQPTWIIPVSVSVPDYVLHYHTHVVGPNQCCSAVVQAISAPIDTVWSLVRRFDNPQAYKHFLKSCHVIDGDGNVGSLREVRVVSGLPAASSTERLEILDDEKHVLSFSVVGGDHRLNNYRSVTTLHTADDDENMTMVVESYEVDVPQGNTKEETCVFVDTIVRCNLQSLAQIAENLAKRKSKLTTTRD

>NtPYL7

MLPNSQNSSYLLQRINSNTSANCKQPHQLQRHSPIPCTTQVPDSVVKYHTHLVGPNQCCSAVIQRISAPVSTVWSVVRRFDNPQAYKHFVKSCHLIDGDGNVGTLREVRVISGLPAVNSTERLEILDEERHVISFSVVGGDHRLVNYRSVTTLHPEPSGNGTTTIVVESFVVDVPMGNTRDETCVFVDTIVKCNLQSLAQIAENLSRRKAP

>NtPYL8

MTSTLQLHRINPNTAATLAGNFHKQPEQPPTWITPLTNTSLPDDLFHYHTHAVGPNQCCSAAVQSISASIDTVWSLVRRFDNPQAYKHFLKSCHVIVGDGEVGSLREVHVISGLPAASSIERLEILDDENHVMSISIVGGDHRLNNYRSVTTLHRTGDDDGTTIVVESYVVDVPQGNTKEETCVFVDTIVRCNLQSLSQIAENLEKIKQ

>NtPYL9

MTSTLQLHRINNNTAATLAGNFHKQPEQPPTWITPLTNTSLPNNLFHYHTHAVGPNQCCSAAVQSISAPIDTVWSLVRRFDNPQAYKHFLKSCHVIVGDGEVGSLREVHVISGLPAASSIERLEILDDENHVMSISIVGGDHRLNNYRSVTTLHRTADDDGTTIVVESYVVDVPQGNTKEETCVFVDTIVRCNLQSLAQIAENLEKIKE

>NtPYL10

MPCSLQLQRISPTTATPAATLAVNLHKQPQPTWVIPVPISNIPKNLLHYHTHVVDPNQCCSAVVLSISAPIDAVWSLVRRFDNPQAYKHFLKSCHVIGGDGNVGTLREVRVISGLPAASSTERLEILDDEKHVISFSVVGGDHRLNNYRSVTTLHPSSTATDDIGTVVVESYVVDIPQGNTKEETCVFVDTIVRCNLQSLAQIAGNFATSKSKSK

>NtPYL11

MPCSLQLQRINPTTATPAATLAVNFHKQPQPTWVIPAPISNIPNNLLHYHTHVVDPNQCCSAVVKFISAPIDAVWSLVRRFDNPQAYKHFLKSCHVIGGDGNVGTLREVRVISGLPAASSMERLEILDDEKHVISFSVVGGYHRLNNYRSVTTLHPSSTAVDDNGTVVVESYVVDIPQGNTKEETCIFVDTIVSCNLQSLAQIAENMATSRSKSK

>NtPYL12

MPCSLQLQRINPTTATPAATLAVNFHKQPQPTWVIPAPISNIPNNLLHYHTHVVDPNQCCSAVVKFISAPIDAVWSLVRRFDNPQAYKHFLKSCHVIGGDGNVGTLREVRVISGLPAASSMERLEILDDEKHVISFSVVGGYHRLNNYRSVTTLHPSSTAVDDNGTVVVESYVVDIPQGNTKEETCIFVDTIVSCNLQSLAQIAENMATSRSKSK

>NtPYL13

MIQRVSAPVSAVWSVVRRFDNPQAYKHFVKSCHVIVGDGDVGTLREVRVISGPPAASSTERLEILDEERHVISFSVVGGDHRLENYRSVTTLHSDPSSCNGSTSTSTIVVESYVVDIPHGNTKEETCVFVDTIVKCNLQSLAQIAENLSRRSMA

>NtPYL14

MPPNPPKSSLLFQRINPTTTPTTSAGATTCNKLQRHTPIPCTAQVPDSVVKYHTQSVSPNQCCSAVIQRVSAPVSAVWSVIRRFDKPQAYKHFVKSCHVIVGDGDVGTLREVHVISGLPAARSTERLEILDEERHVISFSVVGGDHRLVNYRSVTTLHSDPSCNGSTSTSTIVVESYVVDIPHGNTKEETCVFVDTIVKCNLQSLAQIAVNLSRRNMA

>NtPYL15

MPPSSPDSSVLLQRVSSNTTPDFACKQSQQLPRRTMPIPCTTQVPDSVVRFHTHPVGPDQCCSALIQRISAPVSTVWSVVRRFDNPQAYKHFVKSCHVIVGDGDVGTLREVRVISGLPAASSTERLEILDDERHVISFSVVGGDHRLANYRSVTTLHSEPSGDGTTIVVESFVVDIPPGNTRDETCVFVDTIVKCNLTSLSQIAVNLNRRKDS

>NtPYL16

MPPSSPDSSVLLQRISSNTTPDFACKQSQQLQRRTMPIPCTTQVPDSVVRFHTHPVGPNQCCSAVIQRISAPVSTVWSVVRRFDNPQAYKHFVKSCHVIVGDGDVGTLREVRVISGLPAASSTERLEILDDERHVISFSVVGGDHRLANYRSVTTLHPEPSGDGTTIVVESYVVDVPPGNTRDETCVFVDTIVKCNLTSLSQIAVNVNRRKDS

>NtPYL17

MLPNSQNSSYLLQRINSNTTANCKQPHQLQRHTPIPCTTQVPDSVVKYHTHLVGPNQCCSAVIQRISAPVSTVWSVVRRFDNPQAYKHFVKSCHLIDGDGNVGTLREVRVISGLPAVNSTEKLEILDEERHVISFSVVGGDHRLANYRSVTTLHPEPSGNGITTIVVESYVVDVPMGNTRDETCVFVDTIVKCNLQWLAQIAENLGRRKAP

>NtPYL18

MPSSLQLHRINTSTATSTATLAGNYRKQQSQSQPTWITPVSVSVPDYVMHYHTHVVGPNQCCSAVVQAVSAPTDIVWSLVRRFDNPQAYKHFLKSCHVIDGDGNVGSLREVRVVSGLPAASSTERLEILDDEKHVLSFSVVGGDHRLNNYRSVTTLHTADDDENMTMVVESYEVDVPQGNTKEETCIFVDTIVRCNLQSLAQIAENLAKRKSKLTTHD

>NtPYL19

MEAQFIARYHSHQPSDHQCSSSIVKHIKAPVDIVWSLVRRFDQPQKYKPFVSRCTVKGDLRIGSVREVNVKSGLPATTSTERLELLDDEEHILGIRIVGGDHRLKNYSSVITVHPEILDGTPGTLVIESFMVDVPEGNTQEETCYFVKALINCNLKSLADVSDRMSMPVEVFPSA

>NtPYL20

MEAQFITRYHSHQPSDHQCSSSIVKHIKAPVDIVWSLVRRFDQPQKYKPFVSRCTVKGDLRIGSVREVNVKSGLPATTSTERLELLDDEEHILGIRIVGGDHRLKNYSSVITVHPEILDGAPGTLVIESFMVDVPEGNTHEETCYFVKALINCNLKSLADVSERMAMHGGGLPISVNWPSSNQIKT

>NtPYL21

MSPNGFSGVEKEYIRKHHHHHDLAENQCSSFLVKHIRAPVHLVWSLVRRFDQPQKYKSFVSRCIVQGNLEIGSLREVDVKSGLPATTSTERLELLDDEEHILSVRIIGGDHRLRNYSSIISVHPEVIDGRPGTLVIESFVVDVPEGNTKDETCYFVEALIKCNLKSLADVSERLAVQDRTEPIEQV

>NtPYL22

MVAGSSSFYQVWSLVRRFDQPQKYKPFVSRCIVQGNLEIGSLREVDVKSGLPATTSTERLELLDDEEHILIVRIIGGDHRLRNYSSIISVHPEVIDGRPGTLVIESFVVDVPEGNTKDETCYFVEALIKCNLKSLADVSERLAVQDRTEPIEQV

>NtPYL23

MNAIGGLSGVEKEYIKKHHSHDPAENQCTSLLIKHIRAPLPLVWSLVRRFDQPEKYKPFVSRCISQGNLEIGSLREVDVRSGLPATTSTERLELLNDEDHILCVRIVGGDHRLRNYSSIISLHPEVIDGRPGTLVIESFVVDVPEGNTKDETCYFVEALIKCNLKSLADVSERLAVQDMTEPINPM

>NtPYL24

MVSIMNVRSSSEDDYIRRHHRHDIRENQCSSSLVKHIRAPVHLVWSLVRRFDQPQRYKPFVSRCIVQGDLEIGSVREVNVKSGLPATTSKERLELLNDEEHIFGVKIVGGDHRLRNYSSIITVHPEVIDGRPGTMVIESFVVDVPDGNTKDETCYFVEALIRCNLKSLADVSERLAVQGHMEPIDRT

>NtPYL25

MVSIMNVRISSEDDYIRRHHRHDVRDNQCSSSLVKHIRAPVHLVWSLVRRFDQPQRYKPFVSRCIVQGDLEIGSVREVNVKSGLPATTSKERLELLNDEEHIFGVKIVGGDHRLRNYSSIITVHPEVIDGRPGTMVIESFVVDVPDGNTKDETCYFVEALIRCNLKSLADVSERLAVQGHMEPIDRM

>NtPYL26

MEEEYIRRHHRHVVKDDQCSSSLVKRIRAPVNLVWSLVRRFDQPQRYKPFVSRCVVQGDLAIGSVREVNVRSGLPATTSKERLELLDDEEHIFGVKIVGGDHRLRNYSSIITVHPEVIDGRPGTIVIESFVVDVPDGNTKDETCFFVEALIRCNLKSLADVSERLAVQGHTEPIDRM

>NtPYL27

MEEEYIRRHHRHVIKDDQCSSSLVKRIRAPVNLVWSLVRRFDQPQRYKPFVSRCVVQGDLEIGSVREVNVRSGLPATTSKERLELLDDEEHIFGVKIVGGDHRLRNYSSIITVHPEVIDGRPGTIVIESFVVDVPDGNTKDETCFFVEALIRCNLKSLADVSERLAVQGHTEPIDRM

>NtPYL28

MVKRYHTHELSSKQCSSVLVQTIDAPLPLVWSFVRKFDKPQCYKNFINSCTLLSGDGGVGSIREVTLVSGFPGKKSIESFDFLDDEMHVCVFSVVEADHSFLNFKSTITLHEDKEEEDDDDDDGKVLRTSTGKSTNKTVVIESYVVDIPENSCKDDTCEVTDNILRWQLRSLAWVAENMDTSGVSSLDLKKTEISC

>NtPYL29

MVKLYHTHELSPKQCSSMLVQTIDAPLPLVWSLVKKFDKPQCYKNFISSCTLLSGDGGVGSIREVTLVSGFPGKKSIERFDFLDDEMHVCVFSVVNADHSFLNFKSTITLHEEEEEEEDDDGGKVLSSTGKSTVAIESYVVDFPENSCKDDTCEVTDNILRWQLRSLAWVAENMAISGVSSLDLKKT

**Additional File 3. the 2000 bp sequences upstream of *HaPYL* genes**

>transcript:mRNA:HanXRQr2_Chr02g0070891

TAAGCATTCACATCTCTTCCATCTTTTTAACTCTGCAATTTCATTAAAAATAAATATATTCTCTCTCTTTCCAATTAAATCATCTATTTTTAATAATTTTGTTATTCCTTTTCTCTCTTTTTCACTCACAAACACTTTTAAAATATTTTAAATTAAAAATAAATATATTCTCTCTCTTTCCAATTAAATAATCTATTTTTAATAATTTTGTTATTCCTTTTCTCTCTTTTTCACTCACAAACGCTTTTAAAATATTTTAAAAAATTATAAAGGATGAACAGTGTCCCCTCAACTTTACAGATAAATAGTAACATTTTCTCTCTATTTACTCTGAACCACTTCCAATTACCTACAACTCCATTTTATAATATAAAAATTCCAAAATCTAAGAATTTGGGAGACGATTGTAAAAGTTGTTAATAGACAACACACAATAAGCATAATCTTTCGCACATCTTATTGAATCTTGTGGTATCAAGTTGAAAAATAGTATGTATTCGACCAAAAGTCAAAACAAATTTAGAAACAATAAATAAATTACAAGGACCACAAATTAAA

AAACCAACTAGTCCAAGGACTAATTAGACACTACACCTACTTTATATTATTTTATTTATACAATAATTATTATAAATTAAAAATTTATATTTCATACATATAAGTTTTATTATAAATTAATAATAACTTTATAACAATTATACACTCACATGCATATAGTTCACAACACACAAAATATAAATACTATTATCAAACACATATACTAAAAAGAAAATTTATTTTTTAACATTATGAACATACTAAAATAAATCATTAATAAACAAGGGTTAATGAAAAATAAAAGTATAAATTAAAAAAAAATCAGGTATATAGTTCGGGTAAACAGGTATGTGATTTTTGGTAATCGAGGGTATCACCTAATCCCATACCCGACCCATACCCGCGAAAATTTTTAAGACTAATCTCATACCCGGCCCAATACTCGTCGGGTATCAGGTATACCCGTCTCATTTGTATCGGGTTTCAGATATACCCGTCGGG

CTCGGGTATTTTTGTCATCCCTATATGCAATCGACCAAGAGTCAAAACAATTTAAAAACAATAAATTACAAGGACCATAAATTAAAAAACCAACTAGTCCAAGGACCAATTAGACACTACACCTACTTTTAGATTTTAATAAAATATCCCATAACAAAAACACTGAAGAGAGAGAAATAGAGAGTGCAGTGGTGGATGAGAAGCAAACACAAACCACCATTTGGCGTCGTCACTTTCTCTCTCTACAATTTTTCTCTCTCTAAAACGTCAACGGCTCAACCGAATTCTGTTACGTTTTCACCGGTGTATCGTTCAATTTCCGGTAGCATCGTTGTAACGGACCGTCAGATTTCAATATTTGATTCTCATCGTTGATTACTATGACGATCTCCGGCACCGGCGACGGCGACAGCGGCGGTACGAGCTTGACAGAGCGTGATTACATCAAGCGGCATCATAAGCATGAGGTTAGAGCGTGCC

AGTGCACATCGACGGTTGTTAAACGGATCAAAGCGCCTGTTCATCTGGTATGGTCCCTGGTGAGGAGGTTTGATGAACCACAAAAGTACAAACCCTTTGTGAGCGGGTGCAATGTGCAAGGTGACCTCGAAATTGGAAGTGTTAGACAAGTAAACGTTTGTTCCGGACTTCCTGCCACCACAAGTATAGAACGTTTGGAACTTTTAAACGAAGAAGAACACATCCTAGGCATGCGAATCGTCGGTGGTGATCACAGACTCCAGAATTATTCATCTGTCATTACCCTGCATCCGGATATTATCGAAGGCCGTCCGGGAACTTTGGTTATTGAATCATTCGTAGTGGATGTGCCAGATGGCAACACCATAGATGAAACATGCTACTTTGTTGAGGCCCTTATAAAATGCAACCTTAAGTCACTGGCTAACGTGTCAGAGCGTTTGACCGTGCAAGACCATACAGATCCTATTGTCCACGTGTGA

>transcript:mRNA:HanXRQr2_Chr03g0121241

TTCCTCTTCTTTAACACTGTCCATGTTAATAGCACCTAGATATAGCTTCCTTGCCATCTCTGGCGACAGTGGGTGAAACAGCAGGTTCAACAATCATCAAAGACTATGTGCTTACCCGATTTTTCTAACCAAAATTTGAAACCGAGTAGTTGGTTGAGGCGGTAGTTCCCGGAGGTGGTTTTATCGATCACCACTGACTTTAGTATCTGCTAGATATTTTTTTTCCGGTGATGTGGTTAATCAAAGTCTAGATCTTACTTTTCCAGACAGGTAGTGATGGTAGTATCCGTCAATATTGTGGACTAGTGGTGGTTTGGTTATGAAAAAAGCTATAGATGAAAGTACAATTCACTCAAAATGTTAATGTTAATTGTTATAGTATAAATATAAATACTATGTTTAGATTCCTTATAACAATTCCCTGTACTTATAAATACAACTAATTCCAAAAGGAAATTACATCAAAATAGAAAGAAAACGAGCAACAAGTTATGCTTGTTAACCCTTTTTAGATAGTAAAACTAAGTCTTGTTAAAAAGGTACTTTCATAAATTTTAACGTCATAACATTACTATGCATGACTCTTTTGACTCGACTAAGTAGCTCCATAGCTTCTGACGCAAGGAACCCAAAAGTATCGAAAACAAATGGTATAAACACGTGTTGGTTATCAAAGCATGCTTTCTCGTATTTGGTCACTTTTCCCGAAGCAGTTTTTTTAAATAGACAAACCCACCGTGAAAACACTACTTCTCAAAC

CCACAATAGGGAATACTTTTGTTAAGATCTACACACGCATGTTTCCCTCCCATCTATCCAAAGACCAAACCGTTAGTTGGTATGAGTGTTGATCTTTAATGGGCCGGTCAAGAAATTCACAGGTGTCTCTTTCTTAGCAAAACTCCCTGCAAGTCTAAATATTTCAAAAAGAACGTCCCTAACCAAATCGTGCCGGTATATGAACCCCGAGTGCTCTATACAATATAGTGCTTGCTCCCCAAAGAAGTCTAAACACGTCTTACGACAAGCAGAGCATAACTCATCAGCCCATAATACGAATATCATGAGACGATACTTAAAGATAGTACGATACTCCATCAATGACATATGTTAGCCTAACCCATCTATAAAGATAAACAGAAGAAAATTTAAGCATATGATGCTCGTAAACACTCAAAAACCGTTTGTATGTAACTTAATAAAAGTGAACAAAATATATAATCATAAAATAAAACCAAAAACTAAAATACTCTCCGAATTTAATAAAAGACACTCCTTTTCATTGATCACAAGAACTTCATTACACCCCCCTTTTTCCTGCCCACACGACCATTTCCACGTGATTTCTTTCAAACATAACTCATATCTTCTCTCTTTCATAATCAACTAACGACCTCCACTAATATGCAACACTTTCAGTCATCATCTACCCACTTGAAATGGAACCAAAATCAATCCCACAAGGCCTCACAGCAGACGAATACTCCGAGCTTCAACTCTTAATCAATGCATACCACCTGTTCGACAAAATTCCAAACACATGCACATCCCTAATAACCCAACGCATAGACGCGCCCACAAACATAGTGTGGCCTCTAGTACGCCGTTTCGACAACCCGCAACGATACAAGCATTTTATCAAGAGTTGCAGCATGTTTGGGGATGGTGGTGTGGGAAGCATACGTGAGGTTACGGTCATATCGGGCCTCCCGGCCTCCA

CGAGTACTGAACGGCTGGAGTTGTTGGATGATGAGAAGCATATTTTGAGCTTTAGAGTGGTGGGTGGTGAGCATAGATTGAATAACTATCTTTCGGTTACGTCGGTTAATGAGTTTGAGAAAGATGGGAAGGTTTATACGGTGGTGTTGGAGTCTTATATTGTTGATATACCTACGGGGAATACGGTGGAGGATACTAAGATGTTTACGGATACGGTTGTGAAGTTGAATTTGCAAAAGTTGGGTCTTGTGGCAATGGCTTGTTTGCATGGGAGTGAATGA

>transcript:mRNA:HanXRQr2_Chr04g0182281

TACACAAGCCAACTGGTTTTGGTCTAGCCCATTCCCTAAACAGAGATTATTCTTTTTGCCGATTCTCACTTTTGGGCCATGCTACCACCTTTCAAAGCCCATTATCATTTAGCTGGCCTGTAACGGTTCGAAAATTAGAAGCATACATAACTACATTCATAAGTTCTTATATATATACATGCAAGTGGTCTCAAAACATGTAAAAGCGGTGTTTCTCTTCATGTGATTGAGGATTCAAGTCTCACAGTATTAATTCTTGTAGAATTAGGTTTAGAATTAGAAGGTGTGTGTTAGTCGTTCTTATTATGTTATTTATTAAAGTATATTCTAATAGGTGAGTATGCCTACCGATTATTAAATTTGCATTAGACCTTTTCCAATGTTGGGAGGTGGAAAAAAAAACCGTGGCCGACATGAAAGAGGAAAAGAATAGGGAAGACGGATGTACGCCATGGCAAGGAGAGAGGGTGAGTAAAATCCTACACCTCAACTCCTTGCAGCTCTTTTAATACACGGGACTATAACCTTAGTGTATTAATATAGTATATATTAAGAATCCTACACATCTAACTCCTTATCACTTTTGACCTTAACACATTTAACATGTCTCTAAATCACTTTAATTTAATATTTTAATTCCTCTTCAAAATTACATATTAATACAGTAAAACCTCTATAAATTAATAACATCGATAAAGTTAATAATTTGTCCAGTCCCAACTTGGGACTAGAACAAAATTGAACCCCAATCGATAAAATAATAAGATAATAATCTTTTTGAAATCCCAGTGTATATATATTGGTCTTAACGAAACTATAAGTTAATAATCACTAAAAGATTTCAAATTCTAAGACTCCCTAGTACAAAATGAATCTATAGCCACTTGTCTTTTGTTGAATTTCAAATCTAGATTGAGTGCATTGAAACTTGTTTAATAATTTATTTTTTGGTATCATAAATTAACTTTTGTATTTAAAAACAATGAACTTGACAAATTCAATTACTGAGTGAATGTACATTAGACTTGGAAATTTAAAGTTCATGAGTAAAACTACTTTAAATTAATAAATAATAAATTTATCGATATAATAATAATAATGCAATAAAATTTCTTTAACTTGTACTCGTTTCCAAATATTTCTATTGATAATGATAATTGGAGTGACGGATACAATAGCCCAATTGATGATAGTGGCACCATTTGGATGGAGCCACTTTATAATTCTTTCTTTGTCGGTAGTGGATTTAGTGATTCCAATAATTCATAACCACACCACACACACACTATTAGTATTCACTATTTTCATCACAAATTCCCCATCTTCACCTTTAAACAACCAAAAAAAAATTCTAGTTGTTAATCTTCGTAACTTTTTGAACCATACAGCCAGATGTTTCCTTCATATTACACGATTTAAATCGAGATCGAAGAAACTGATGAAGAATGGATGCAGCTCACTATATAAGAAGACACCACAGGCATACTCTTGGACATAAACAGTGCGCTTCTGTCGTCGTTAGACACATCAAAGCTCCCGTTGATATCGTGTGGTCATTAGTAAGGAGATTTGATGAACCTCAGAAGTACAAGCCCTTTGTTAGTAGGTGCACTATGCATGGGGAGATTAATATTGGAAGTGTTAGAGAGGTGAATGTCAAGTCAGGGCTTCCAGCCACTACCAGCACAGAGAGGTTGGAACTTCTTGATGACAAGGAGCACATAC

TTGGCATCAAAATTGTTGGTGGTGACCATAGGCTAAAGAACTATTCGTCAATCCTAACCGTTCATCCAGAGGTAAGCGAAGGATCTGGGACGTTAGTTGTGGAGTCATTTGTGGTGGACATACCCGATGGCAACACTATGGAAGAAACATGCTACTTTGTTAGAGCCTTAATCAATTGCAACCTTAAATGTCTTTCAGATGTCTCGGAAAGAATGGCCATTTGTCCGGATTCAGGCATGAAAAGATCAGAAATGGCTCATGGTGTTCCGGTTTAG

>transcript:mRNA:HanXRQr2_Chr05g0211711

GGTTTTGGATGGTGGATTAAAGATTAGTTACATGACACTAACATGGAGGGTCTTGGTGGTCATGAGGGTCATGACCATACCCTATAGCCTTATGAGATTATATATTATAAATTTAGATAGCTTAGTTTAAAAAGTTTATTAAAGTTATATGCATTAGATTATTAATTTAGATAGCTTAGTTTAAAAAGTTAAATAAAGTTATATGCATTATAAATATGGAAACGTTTCTTTTTCTATTAACCAGTTATGTATTGAAAGTACTACCGATGATCCTAATTAGGCATCGACTAGAATCCTATATATCCTTGACACACTTCAAGTTAAAACTATTCTTTTAAGATAAGTAAGTTATAGGGTTTCCTTCTAGAAACATCCAAAAACATTATAAAAGTAAGGATTTTTTTTCTATACGAT

TTTATTGTTTATTTACATGCTAGAATAGAAGGTACATGAGATTGTTAGAGATGTTTTGCATTTTTTTAGTAGAAAACTTAAGGTTGTTATTAACATGTTTCTAGCACTATGTTTTTAGATCCCAAATTAACATTTGCAAAACTATAGTATATAACGTGAACATTTTGTTAGAATGAGTAATGATAAGTGGACTAGGGGTGGTTCACTAGTGATAGAATTTATCACTCACAAGCACCAATCAAGTTTCGCCATGTCATTGACCATTTTTCCATCACTCACAACCATTTTTAGTGGGGGTGGTCATCACTCACCACCACACCCAACAATTTCCCTCCAACCAACAAATACCCTCACAAAAATAAACCATAACGCGTTGTCACCATCACGAAAATGATTAACGCGTGGAAGATTAACGCGTTGAAGTGGTAGCGGCGGCCGGCCATCACGCGTGGAAGATTGTTTCCGTTATAGTATAACGGA

GTGCCCCGTGCTCTCTAATGTGAGATAATTTTCTTTTTAGGCCCCATATTTTTAGTTTTGAAAGTAATATTTATAAAAATATAGTGTGTAACAGGTACATTTTAGATAGAAATTACACATGAGAGTACTTGTAATACTTTAAATACTGTAACATTTTTTTTTTCTAAAAAACTAATAATTTATTAGTACGTAACTTAGAATGAGAAAAACGTTATATAACTTCTTTCTAGAAATATGCATAAAAAAATTATAAAGTAATAATTAATAAAGACAAAAGGCATCTGAAATTAAAGTTTCTATGCAACTTGCTTCTCCTACTTGCATGACACCCAACCAAATTCAAACTAATTGCATGCAACTTGCAAACCCCTTTCTCAATCTTCACATATATATATATATTTCATTTGTATACATATATACACAAATAATATCATATCAATTATCATATCACATGCTAAGTACGATCCTGTCAATTCATTCAATAACAAAATCAACCAATCATGTGTTCCTCCATTCAAATCCAAAGAATACAAGAAAACCCTAAATCCACCGTGAACCACCACACCACCACCACCGTACGGAGGGTTCCACCGTCAGTTTACCTACCAGAACACATCCACCACCACCACATCCATACAATGACTCTCAACCAGTGTTCGTCGACAATAGTCCAGACGATCTCTGCTCCAGTAGAGACAGTCTGGTCTGTGGTTCGTCGCTTTGACGAACCACATGCATACAAGCACTTTCTCAAGAGCTGTGACGTCATCGTCGGTGACGGTGACGTGGGTTCTGTGAGGGAGGTTCGGGTGGTATCCGGGTTACCCGCGGGTTCCAGCTTGGAGAGGCTTGAGATCTGTGATGACGAACGGCATGTTATGAGCTTTAGCGTTGTTGGTGGGGACCACCGGCTGAAGAACTACCGGTCGGTTACGACGCTTCACTCTTCGCCGACATGCCGCGTCACCACCGTAGTTGTGGAGTCTTATGTTGTGGACGTGCCACCGGAGAATACTAAGGAGGAAACGTGTGTGTTCGTGGAGACAATCGTACGATGTAACTTGATGTCCCTTAAACATATTGCTCAAAGTTTAGCCCAGAAATAA

>transcript:mRNA:HanXRQr2_Chr06g0246931

TTTACTTTTTGAGTAAACTGCCAAAATGGTCCCTGAGGTTTGGTCACTTTTGCCACTTTAGTTCAAAACTTAAACCTTTTGTATCTAGATCCTTTTGGTTTCAATTTTATTGCCATTTTCATCCAAAACAAAATATGGTCAGATTTTTCAGTTAACATCATATTTTTTTTTGTTATCTTTTTTCTCCCTTTTAATGAATGGCAAACTGGTTAAATTTTGTTAATTTGTTATAATAAAACATTAAAAGACTATTTTGCCCTTCATTAAAAGGGGGGGAAGACAAAAAAACTGGATGTTAACTGAAAAATCTAACCAGATTTTGATTTTGGATGAAAATGGCAAAAAATTGAAACCACGGGGACCTAGATTCAAAAGGTTTAACCAAACCTCAGGGACCATTTTGGCAGTTTACGCTTATTTTTTTATATTCCAGCATAATTTTGTAAAAAAGCGTATTATCTTGGCATGATAAGCTATAGCGAGTGTCCGCACCGTGACAAACTGAACCGGTACATGTGAATTTATCTGTTTAGGCATATCATAATTTTATTTTTTGGATTTCCTTTTTCAGTTTTTTTTAAAGGTGTGAATTATTCGTGAATGTCAGGAGGTCTAGCATACGTTGTTTTAACCGGGTCCACGTTAGAGAGCCCCCTCGCACAATAGATCCCCAATTTAAACTCCTATCACCCATACTCGAACTTGAGACCTCGAGGAGAAAACTCATCCAAGCCCATCATAGGTGGAACTTAAATACCCGTGGCCATAACTAAAGCACTAGTGATGATGGTTAAACCCATACCGAACTTTAATGAAAATAAATAGGTCAAACCTTAGATATACCAAGTCTACTCTAACCCTAAGCATCTAACAAAATTGGGTCGGCTTGGGTGGTCAGAGAAGGAAGTGTTCCACGGCATTGTCATTCTTACCTGTTGGAGTATTTGGAAAGCTAGGAATAGTTTGGTTTTTTCGAGGAAGCCTTTTAAAATTGACGATGTTTTCAGTGAAATAAGGTCTTTGGGTTTTGTTTTGTAGATCGAAAGATAGATGTATAGCTTGAAAGGATTGGTGTAAATTTGAAAATATATAATCGGGGTTATTGGTTTGGGATTGGCCGCTCGGTTTCGAGCTTGCTTTTGTTGTTCTAATGAAGTTGTTTAAAAAAAAAAACAAAACAAAACATGTTCACAAATTTAACCTGACCCCGTTTTAAGTAAAGACAAACCCATAAAATTCATCTCATATTTGGGTGAGTAATGGAGACAATCACCCAAGGATAATGCCAATTGGGTGGAATCACTTTACATGATTTCGTTTGTTTGTTAGCGATTTTTATAAAATTATACGCACATGCACTATTCCCACCACTAATTCCCCATCCATCCTTCTTCTTCTTCTTCAAAAGTCAATAACTTTTCGATTCATTTCATTTCTAGGGTTCCAGTTTCTCCTCTAATCGCACAACAAATTGATCATCAATGGATGCAGCTCAGTATATACGAAGACACCACAGGCATAATCCCGGCCGTCAACAGTGCGCTTCTGCCGTCGTTAAACACATCAAAGCTCCCGTTGATATCGTTTGGTCATTAGTTAGGAGGTTTGATCAACCTCAAAAGTACAAGCCATTTGTTAGCAGATGCACGATGCGTGGGGGTCTTAACATCGGGAGTGTTCGAGAGGTGGATGTCAAGTCAGGGCTTCCAGCCACTACCAGCACAGAGAGGCTGGAGCTTCTTGATGACACTGAGCATATTCTCGGCATCAAAATTGTTGGCGGTGACCATAGGCTAAAGAACTATTCGTCGATCCTAACTGTCCATCCAGAGGTAATTGAAGGAAGATCCGGGACGTTAGTCATAGAGTCATTTGTGGTGGACATACCCAATGGCAATACTCAAGATGAAACATGTTACTTTGTGAAAGCCCTAATTAACTGCAACCTTAAATCTCTTTCTGATATCTCCGAAAGAATGGCTGTTCAAACTCAGATGTGA

>transcript:mRNA:HanXRQr2_Chr06g0242911

AAATTTAAACTCTAAACCCTAAAAGTTCAACCCTATATGTATTTGAATAGGTTCGACGAGCCAGATCTAATGTCTTTTGAACGAGGTCAATCGGAGTTTGTTTGACCTGGTTTCCTACACTTCCTAGGTTAAGATTGTTTTATATTTGATCATTCCATATCATAGTGTAATGTAGTGTAGTGTAGTGTAGTATACCATAGTCTTATACGTCACATATAGACCTATACGATTATACGAGACTATACATGCCCAACCGTTAGACTTGATATGATGGTAACTATATTTTTTGAAAAATGAAAAAGCTATATGACACGTATAACATTATACATGTCATAGAAGGTAAGAGTGTCCAAATAAGAGCATTATGCTTTACGTGTCCACATTTCGGACCACCAGTGTGGTAGCACTTTGACTATGGTGGTCCGGTTAGTCCGCTGAACTGGTTCGGTTGAAATGGTGGGGACCACACACCCACAAGCCTTTTTTTAATGTAAAATTGTGTAATGTGAATGTTTGAAATGGTTTAGGGTTGAAGAAAAAAAATAAGATCTA

AAACGGTTAAGGTTTAAAATATTGTGACAACCCTGAATTTTAAACTTTAATCAGTGTTGTGAATAGTAGCGGGGGATGTTCAAATAAGTAATAACCAAAGCCATAAATAGCAAGGTTGGAGAACTCGCTAGTCGGCCGGTGAAGTTGGGACGAACGACTACTCAAGATTGCTCGGGATTAATCGGACCGCGTCTTTTATATGTAATCTTGAGTTTTATGTATACATATACACATTTTTATACTAGATTAATTATTGGAATTCACATTATTTGGTTAGAAACTGGTCGGAATTTAGAGGTTTTGGCCGGAATATACAGGTTTTTTGTCAGGATCGCCAATTTTTGGCTGACCAACTAGGTCCAACTTGGTCTGATTAGGCCCGACTAGGCCCAACTTGGTCCGACTAGACCCGACTAGCGTTTAGTGAACTGAGTAATGAAAAATTACTTAGTTACTAACCGACTAGCGATTAATCACCGACCAATCACGATTTTTACAACATCAATAAATAGTCTTTAACTCATGGTCCAAAACATTGAAAAAAAATACATTTTCCTACTTTACCCTCACGCTACCTCAAAGTCATCTTTTATAAACTAAACAAATCTAGTCCTTATCTATCCCCCACACCCTCTTTCTCTCTCCTCCTCACTCACCCGGATCACCCAACGTCAACAAACATGGAGCAAGCCGGCACCTCCACACCGGAACACCAAAACCCACCACCACAAACCACCACAACTACCCACCACCTCTCCCTCCCACCAGGCTTAACCGAACACGAGTTCAACCAACTCAAAACCTTCGTATCCAACTTCCACACCTACCATCTCTCACCCTCCCAATGTTCTTCCCTACTCGCCCAACACATCCACGCGCCGGTCGACGTCGTCTGGTCCGTCGTCCGCCGGTTCGACAAACCCCAAACTTACAAACACTTCATCAAAAGCTGCACCGTCGGTGAAAACTTCAAAATGGAAGTGGGGTGCACGCGCGATGTCAACGTCATCTCCGGCTTACCGGCGGCCACTAGCACCGAGCGGTTAGACTTGTTGGATGATGACAACCACGTGATGGCGTTCACTATTATTGGTGGTGAACACCGGTTGAGAAACTATCATGCTGTCACGACCGTTCACGAGGTTGTTACAGAGAATGATGATTCGGTTACGGTTGTTTTGGAGTCTTACGTTGTTGATGTGCCTGAGGGGAATACTGAGGAGGATACGAGGTTGTTTGCTGATACTGTTGTGAAGTTGAATCTGCAGAAACTGGCGGCGGTGACGGAAGCGATGGCGGTTGACGGTGGCGCGTCTATTAAGAATCTGAATCTCAGGATACACCCATTTTCGAAACGGGAAAACCCCCACTTAAGGCCGAAGCACGGGAACACTCGTTTGAAGTCATGCCAGTGCGGTGAGGTAAAACCCAGATGCCACCGAAACTAA

>transcript:mRNA:HanXRQr2_Chr06g0241551

AAATTAAAAATAAAAATAAAAACAAAAGTCCCGACTTCCTTATTCTAAATAAAAATAGTGATAACTATTTTGAGACTTTTTAAATACTAATAAAAACAAAAGTCCTCACTTCCTTATTCTAAATAAAAATAATGATAACTATTTTGAGAATTTTTAAATACTAATAAAAACAAAAGTATATAGGTTGCGATTCCCGCTATAGCTGGGTGGTAGAGACTCTTGCCTTTTGAAGAAAATACAAAGGTTCAAATCCTGTGACATGAGGTGTAATTTAGTATTTATTGATGTACTTTCAGTTGATATTTACTAAACAAACCTTGTAATTCATTTTACTACGTGAATATCGTATACATGTATTTTTTCGTAAATCAAATATTCAATTGTATTAACCGTCGTATATATACACAAACAAACTCATGTACATGTACAACACAATAATAAGATGCTACATTAGTCAAAACTACTAAATGACTAAGGATCTAAATCAACCCATGAAAAAACCTTAATTATATGAACTATCAACTTGTAAAAATGAACATTTGGTAATGAATTTGAAGATAAACAAGCATGTAGAGTTGAACCCATAAATAACTAATATTATGAATGGGGTCATTTTGAAGAGTTTAAAGACTTTTTTTGAAAGACAAATTGAAATCACTGACAGACAACTGGGGTATCGTCATGCCACCAGCGAAACTACCCTATCATATCATCTCCACTCGACTATAATGCCATGATCTGAAGACTACCTTGAAGGATTATCGTACATTATCATATGTTATAGTGAAAGATTTCATGGGATGGACTAACATATGATATCTCTCATGGAATTCGAAAAGGGGTTCTTACATTTCTAACCATGACAATTTGTAGTCAATTTAAAGTCGGGGTCTAGTTAGGAGTAGCTTCACTATCCCTAGAAGTAAGTATAAGGTTTGTCTCCATCTCATCTTCCTCAAACCCTACCAATAGCATTATGCTACAAGTTGACTTATATTAGGCTTGTTTGTTTTCGAAACAAGTGAACAAAATCAATCAATTTGCGACTCCAAACATCATAAAGAGCATATCTCATTAACAAAGTAAAACCATACAATTATGATTTCCTTTTTTCTCCCATTATATGTATTAGCATAAGTGCCATAAGCCCATAAGCATCTTTGTGTGATG

CCAGAGATAAGGACTAAAAATACATTTCCGAATAGTCTAGGGATCAAACAGAAACTTTGACTATTTTAACATCACATGAAACCGGTGAAGAAACAAGATGTGGATGAGAAGAACCAAAAAGCAGTTAACGCTCAAGAAACCCCCAATTGGTCACCTGATTATTCCTTCTCGAAACTCTCAACCGACTTTGTGCCTTTATTAACCGCACCGATCAAATTCCGGTCACAGTTCGCCGTTGCAGTTACTCATAATAATCACCGATGGCGCTCACCGGAGACGACGACGCAACAGATTCGTCGGAGAACAGTTACATTAGAAAACACCATAAGCATGAGGTTAGAGACAATCAGTGCAGTTCATCTCTTGTTAAACACATTAAAGCGCCTGTACACCTTGTATGGTCTTTAGTAAGGAGGTTTGATCAACCACAAAAGTACAAACCCTTTGTCAGTGGGTGTAATGTGCAAGGTGACCTTGAAATCGGGAGCGTGAGAGAAGTGTGTGTTCGATCTGGGCTTCCCGCTACCACAAGTATGGAGCGGTTAGAGCTTCTTGACGAAGAAGAACATATATTGGGCATAAAAATCGTAGGTGGTGACCACAGGCTGCGGAACTACTCGTCGATTATCACGCTTCATCCGGAGATTTTAGACGGGAGGCCAGGTACTTTGGTGATTGAATCGTTTGTTGTAGATGTGCCGGATGGTAACACGAAAGATGAGACATGCTACTTTGTGGAGGCCCTCATTAAGTGCAACCTTAAGTCATTGGCTGATGTGTCTGAGCGTTTATCGGTGCAAGACCGAACAGAGTCCATAGTTGGAGCGTGA

>transcript:mRNA:HanXRQr2_Chr07g0285411

CCAAAAAAGTGTAGGAGTTGGATATCTTGGTGAATGGTTGAGCGCTAAAAAGCATGTAAATAATAGCTTTTAGTTTTTGCATTAAAAATAGTTTCCTATCTTTAATCAAGAATGGACCAAGTCACCAAATGACCAAAGATTGTATTAATGTTAGCTTTCTGTTTATAGTATGAAGTTACATCATATCCTAAAATAGAAAAAAAGTATAACTTCTAAGGAAGAGTTTGGATGACTATAATTGATTAAAATAGTTGTATGATGGGGTCATGCTGCACAAATTTGAAAATTTAAGTTGACTCACAACAAATTATATAACAAATCGACCATCCCTTAATTGGTAAGAAATCTCAATCATAGCCCTCACCATTTGGTCGTAAACAAAAGACAAACTACACTCTCACCCCTTTGTGTTCATCTTATTAACCTTGTGTCCTTGTAGTAAGATCCTACTTGTGTTTTAGAACCATACAAATCTACATTGATGTGTTCTTTAATAAATTTAATATATTATTTAAGGTTTTCATGTTCTTTAACAAATTATTTAAGGTTTTCCTTGTCGATATACTTCTCATAATCATTCTTATTTGTACGACGAGTGGTAATAAACAATAAAGTTTCCTTTTTTCTAAAAAAAAATAAAATTCGTACTCCTTAAAGTCACTAACATGTATAACGGGTTTTCATTATGAACTATAGGTTAGCGTATTTATAGCTTTGAATCCAATAGTTTAAGTCAAATAAGATGGATTGAGTCGCCATCTTATTATCCCATAATCAATCTTGTGATCTTGATCACAATGTTATGATTATAGTTATCATATGCTCTATATATGTATTTATTATTTTTTCCCAACCTTACAAACACAATATTTGTGTTGTGATGAATGTTTATATTTAGAAAAAAGGGTATTTGAACCATTTTTCAAAGAAACCGAGTCAGTTGATAAGTGTTAAAAAGATATATAACTTAATGTTCATCTTTAAGAAAATAAAAAAGAAAACTATATTTCTTATAAAAGATTCAAAATTACTTCCATCCCAACACCCTCGTAAACCCAAATGCACACCTTCAAACCAATCTCAACTCCAAACAAAGGAGGACTAAACTGAAACAAACAAAAAGAAAAAGGTGCAAATCTAACTTTTATCTTAAACCAAGGACTTGCAACCAAAACTTTGAAAGACTACTACCCTAGAACTTTTATAAAATTCATAAAAGAAACCCCTCCACCTCCATGGTTCATGTGCCATTGTCCACCAAACTCTCTCCTTTCACTTTCAAAACTCATCACAAAAAAAAGATAATAAATAAAAAAAAAATTATTACCTCATCAAATCTCCTACACCCCCAACCCAATGATCCAATAAATCTAAGACCTTCCAAACCCTCAAAGCTAATCACTCTTCTAGCTCTCCAAATGGACAGAAACACCAACTCAAACTCAAACTCAATCCCACAAGGCCTAACCCAAGAAGAATACACTCAACTCCAACCCTTAATAAATGCATACCACATGTTCGACAAAATGCCCAACACATGCACCTCACTAATAACTCAACGCATCGAGGCGCCCGCCCACGTGGTCTGGGCTTTGGTACGGCGGTTCGACAACCCACAAAGGTACAAGCATTTCATCAAGAGTTGCAGCATGATCGGTGACGGAGGTGTTGGGAGTATCCGTGAAGTTAACGTTATCTCGGGCATCCCGGCGTCCACGAGCACCGAAAGATTGGAGCTGGTGGACGAGGAGAGGCATATTTTGAGTTTTAGAGTTGTGGGTGGTGAGCACAGGTTGAATAACTATCTTTCGGTTACTTCGGTTAATGAGTTTAGTAAGGAGGAAAAGGTTTACACAATTGTGTTGGAGTCTTATGTTGTTGATGTTCCTGAAGGGAATACGGTAGATGATACCAAGATGTTTACGGATACGGTTGTCAAGTTGAATTTGCAGAAGTTGGGTGTGGTGGCTATGGGCTGTCTGCATGGTGGTGAATCATAG

>transcript:mRNA:HanXRQr2_Chr07g0313141

ATTGTTGGTCGATCGAAAGGTTTTTACATGTAACAATGGACAGGGACAATAATTTATATATATTTAGTCAAAAAAGAGCCCACTAACATCATAGTCGGTGGTGCCAAAGATTCATGCATGGCTAGCTCATAGATCTACTTTCTCCTGATTCCCGTACCACCATCAAAAACCATAAAACTGCCTCCTTTGATAATATTTTGTGTATTATCCAATAATAATGTATTTCACACACACACACAAAAAAAAAAAGAAGATAAAAACCATTAAATCTAACACTCTTATTTAGCATGAAAAAACTGTTAAGATCAAATTTTTACATGTTCGAAACTTGTTACAATACACAAAAATCTCATAAGCGAACTTTGGTACGCAAAAATTACATAAACCGAACTTTGTTTTGCACTAGGCTTTTATTAAATGATCTTCACATATATTATATAAATGACTTATATTTTTTTCGTTTCTGATTTGTAGTTTTTGTTATACTATATAATTTATTACATTCTTCGTGTAAATTGAACTTATTTTTATATATAGTTCGTTCTTTTAATTTTGTATTTATATATTTATTAAATTTCGTAATTTAATCCGGTTATAAAGCGGTACAGTGAACCAATAAGGAGACTGGTTTGATTACCAATCCAACTTTAAAAACATTATTATGTGGGTTGTGTTCTTATTTTTTTAGGCTTTGTCTTTCGCTGTTTAAATTAGGTGAGTTAACACCCTGTATTGTGCATGATTCAACGTTTTTACTTTTATTTTTGTCCGGTTTAAGAGTCCCGCCGCAACGTGCAAGTCCTAATAACTTGTTTAAACAAGTTTAGTGATTTTAATATTTCAAAACTGAATAAGTTATTTTGTGCATATAGTATAGAGACCTTCTTACGTTAAGTTAGGTTAAACATTAAACTACCTTTTTTCCTCTAAAGTCTAAAGTATGCTAGTTTATTTAACTAAAAATTTAATTTAAATAGTTGTACTATGAAATATCAATTATTAATATACGTTTTTTCATGTATATCATATACTTTTTATATGAGTTCTGACCGGCACCTACTCCTGTCAAAGCAAAACTTAATGTTCCACAGAAAAAATAAAATAATTCCATAAACCATTATTCCGAAAAAGAAAATACATATAAAAAGAAAAAAATATTATGCAACTTGCTTGCTCCCACTTGCATGACCGGCGTAATCCCTGTGTTTCTTCCTCTTTAAACTTTTCCACCATCACAACTTGCAAGTTGCAACATTGCCTCCCATTCCTGACGTTATCTCCATCTATATATACACATATATATCTATTATTCACCATCTACACGCTACAAAAAAAGGTTTAAACTCAAGACTGAATAATTATTATCAAATATGCCTTCTCCCATTCAGATTCAACGAATCCACCCTACAACCACCACACCCACCACCACCACCACCGTCAACCACCACAAACAACCACTCCCCACAACCACAATATGGAGAGTACCATCATCTATATGCATCCCAGAAGACCTTATGCACCACCACACTCATGTCGTTGGTCCAAACAGATGCTCATCCGTAGTTGTCCAGACAATATCCGCACCAGTGGATATTGTCTGGTCTGTCGTCCGTAGGTTTGACAACCCGCAGGCGTACAAACACTTTCTCAAAAGTTGTCACGTGATTCTTGGTGATGGTGATGTCGGCAGTTTAAGAAAGGTACATGTTGTGTCTGGATTGCCAGCTGGATCCAGTACGGAGCGGTTGGAAATACTTGATGATGAGCGGCATGTTATGAGTTTTAGCGTTGTCGGTGGTGACCATCGGTTGAATAATTACCGATCTGTCACCACGCTGCACCCGTCGCAGTGCGACGGATGCACTGTTGTGGTGGAGTCGTATGTGGTGGATGTGCCTGCTGAGAATACTAAGGAGGAGACGTGTGTGTTTGTTGATACTATTGTTAGATGTAACATGATTTCGTTGAAGCAGATTGCGGAAAATCTGGTGAAGAAGTAG

>transcript:mRNA:HanXRQr2_Chr07g0316871

TATTTGTTGTTATGTTCATATTACAATTTAGAATCACAGTAATGCGAATGCTGACATAAGATATTTCATCAACTTAGTAAATAAACCACTACTAGAAAACTGGATAGTTTTACACACATTATTTCAACAAAGTAAATAAATTATTAAAAAAAAAAAGCTTAATTATATACATATGAAGAAAATGTTTCTTCATTTATTTAAATAATATAGCATCCAAAAGAAATAGTTTGTTTTTTTTTTGAAAGAAGGCGGATTTATTAATAGAAAAACAACAGTTTGTTAAGAGTATTAAATCCAATTTGGTACCAAAAAAGAAATAGTTTCTTGAGAGTATTAAATCCAATTGTGGTACCAAAGAAATCAGACCTAGAAAAGTAAGGAGCACTATAAGTGATTTATAGTGGATCATTGAAAACTTTAGGCAGCATAGCATAATGAATTCTCATTGTAGGCTTTAGGCTCCTTAATAAAAAAACCATACACCTTTATAAACCTAAATTTGGGATTTTTTTATATCTCTTGGGGTACATGAGTTAATCGATTGTAGCTCGAGTAAACTCAAGTTTCTTTTGTTTAATTAGTAGAGATTGAGCTTGAATTACTTAAAATGAACATTAATTCTTCAATTTTGCAATTTTAATGAAAAGATCACACATAAACATGAATCATGATAAATACGTTATTTCATGACATGGCAAAATTAAAACACATAGGGTAATATAACGAAATGATACATTTCATAACCATATTATCATCCTTTTTTATGATTTATATTAAGTTTACAATTATACGTGTAGTTGGTTAAATTCCGACACACTTACTTCTTTGTTTCCGACTCATGACTTAAGTGGTTTACTGGGATGATACTTATCTTTCACAAAAAGACATGAGTTTAAATCCTTGTAAGTTAAAGGTTAACTAGTATTTAGATATAAAAAAAAAGGCTTGGTTGAGTAGTAGCTTCGTGACTTTCCAAATGAGAGGTCTGGTTGAAGTCTCATCTAAGAAAATTTGTTTTCCTTCCTGAAGGGTGAGTCGGGTCACCATATTTTATTATTTAGACGAAAGGGTGATCCATTTACCAGGGTGATCTTGCGCGTGTTTGCTGTTAAAATAATGCTACGATGTAGTCATTAATTATTATATCAATAAATGAGCATATGTTATGAGTTTAGGTTTTACCCCCACCCAAACACAACGAGACAAAACGAAAGACAAACCGACAAAAAACAAAAAGAATGGCAAAATGGAAATTAGTAAGGTATGGGAGGCTATTTACAGATCGATTGGAGATAACACGGTGAGGAAACGACGGAGTACGGACCTGATGACCCGATGAAGAAGCCCCCCGCCCAGCTCTCCTCTGAGTCACACAAACGATTCTTTTGTTTCAGACATCATCAGTGATCGGAAAACACAAGACGATGAGCAGCTGCCGTGATAATAATAATAATATTAGCAGTTTGGAGTCGGAATACATTAAAAAACATCACAGGCACGAGAATATTGCCGATAATCAATGCACCTCCGTTCTGCTCAAACACATCAAGGCCCCTGTTCATCTCGTTTGGTCATTGGTAAGGAGGTTTGATGAGCCGCAAAAGTATAAGCCATTTGTGAGCAGGTGTGTTGCTCAGGGCAATCTTGAGATTGGTAGTCTTAGAGAGGTTGATGTTAAGTCTGGTCTTCCTGCTACTACCAGTACCGAGAGGTTGGAACTCCTTGATGATGATCAGCATATCTTTAGCATTCGGATTATTGGTGGTGATCACAGACTCAGGAACTACTCATCGATTATCTCAGTCCACCCAGAAATTATTGAAGGGCGTCCAGGGACACTAGTGGTGGAATCATTTGTGGTTGATGTGCCAGAAGGGAATACCAAGGATGAAACATGCTACTTTGTTCAAGCATTGATCAAGTGCAATCTAAAATCTCTTGCTGACGTGTCTGAACGGCTTGCAGTCCAAGACAGGACTGAGCCCATTGACCGAATGTAG

>transcript:mRNA:HanXRQr2_Chr08g0342731

GAATTATTCGGCTATCGCCCCGCCCAATCGCCCCGCAACGCGGGCGGGGCATCTACTAGTTGTATAACATTTTCACCATCCCTTAACTGGTAAGAAATCACAATCACAACTCTAGACCTCACAATTTGGACATAAATTATTAAGAAAAGGACAAAGTGCACTTTCACCCCCTTTGGTTTCTTCTAAATTAACACTGACCCCTTTAGCTAGATCCTACTTCTCATTATTTGAAAACTATGCAAATTTGTTTTGATAAGCGGCATTCTTGGCGATATCCTACTAGGAATTATCCGTATCAATTAACTAATATTTGGGAAAAATTATATAAAAAATCATAATAGGTTTAAAGTTTGGATATGACCGTACTTGATAGTATTATAAAACTAAATTAAGTCAAACAAGATTAATTGGTTTGAAATCTTATGAATGTGTGGTTGGTCTTGTGATCCTTGTTATGGTCTTAGGTGATAGTGATTCTATGCTAGTGGCATGCTTTGTTCAATTAAGATTTTATGATCAAGAGAGGATCCTAAAATCCATGACATAAATCCTTGAAATGACACAATGAGCCCCTTTGGTCTAGCAGTACTAAAAGGATGTCAAGCATACGTAAGGTGATGGTTTGAACTTTGAAGTTTCGTTTCAAGTACAAGTGAATATGTAAGTCTTCGTCATTTTACAAAAATGTAATCTTTAGTAAAACAAAGATGAAAAAGAACATTAATTAGCAAATAACGAAATTTGGTTAAAATCCTACAA

GAGAATTACACCGTTACGTTTTCTAATAACAACTTAAAATGAGAAGGCTATGTTTAAAAAATTGTTCATGGTTTTAAGAAACTATATTTTCATGATTATAATAAACATGAAACAAATTTTATGAACATTAATTCTCTTCATAAACATGGTATCATGCCTCCCTTATCTTAAAACCATAAATTTTACTACAAATGTTTGTATCCAAGATAAACATGTTAGAGGTCTTTAGATACACACTTTCAAAATGCCTAACTTCAATTACCACAATTAACTAATCATCAACTAATCAAAGTGAAAAAAGTATGTATTAAATTTATCTTTCAAAACCATTAACACTCTCTTGAACCCAAATTGAATACTTCTTTTTTGCAATCTCCAAACACATGACTTGATCAATGTATAAAAAAATAAGAGGGTCATATCTAATTCTTGCTTTAATCTAAAGGGCAAGCAAGTCAACTTTTATACACCTTATATAAACAAAACCCTCACCTCCATTGTTCATGTGCCATTGTCCACCTTCACCAACCTCTCTCCTCACTTTCAAATCTCATTAACTACCTTTTGTACATCTTCATACTCATTTCCATGTGATCTTATTGTCAACTCATCTCCACCCCCCCCCCCCAAAACACACACACACACACAAAACCCTTAAATCCATTCCTCCACTTCTCAAAATGAACACAAACCCAATCCCACAAGGCCTAACCCAAGATGAATACTCCAACCTCCAACCCATCATCACCACCCACCACCTCTTCACCAAAACACCAAACACATGCACCTCACTCATCACCCAACGCATCAACGCCCCAGCCCACATAGTCTGGTCCTTAGTACGCCGGTTCGACAACCCACAAAGATACAAACACTTCATCAAAAGCTGCACCATGACTGGTGACGGTGGTGTGGGCAGTATCCGGGAAGTTACGGTCATCTCCGGCCTCCCGGCGTCTACTAGCACCGAAAGACTAGAACTAGTAGACGATGAGAAGCATATCTTGAGCTTTAGAGTTGTGGGTGGTGAACACCGGTTGAATAACTATTGTTCGGTCACTTCGGTTAATGAGTTTAGTGAAGGTGATAAGGTTTACACCATTGTGTTGGAGTCTTATGTTGTTGATGTTCCTCAAGGGAATACGGTGGAGGATACTAAGATGTTTACGGATACGGTTGTGAAGTTGAATCTTCACAAGTTGGGTGCGGTGGCTGTTGGTTGCTTGCATGGTGATGGGTGA

>transcript:mRNA:HanXRQr2_Chr08g0334961

CTATTTTCAACATTTGTATAAACCACTTGTTAGAATCATATGTTATAATCGGTCCTTGTCCTATTCTTCAACTTATCTACATTTCATTGTTTACATGTCTAATATTGTTTTGCTATGTTACGATATGATTTTCTCTTAATTAATGAATTAATTGCATACGGTCTAAACTGCATTTATTAAAGAACATAAAACATAACATTATTTAATTATAATTTCTCTTTTATTGTGTTGATCGTAATACCGATAATTTCCCGAAATTAATTCTCACATTGTGCAGCCTTATATATTTTTCCTCCATTTTAGTAAAAATGATTTTTTTTTATATTCTATATTACTAGGTTATTTAAATTAACGTAAATAAATCTAGATACAAAACATATCCAGTCTAAATACTACTATTAGTTAATATTGGGAGTTAAATAACGTGGATAGTGAATAGAGATAGGAAAAGCAATGTTAGTTAATCAGGATGGTTACAAAACCCACTTCTTTTTGTGTAATGTTTTTCTTTTGCTAGCTTTTAGCTAGTCGATTATTAAAAGAAAGTTCGCTGTTAAAGTTTGAATAAGGTGTTTTATAGTACATGTTGAAGTTAATGTTTTTAAAGGGATATTAAATTTAAATAACCCAAACATTCATAAAAAATGTAAGAAAGTATTTCTGGAATATAATATAATACCTATATAAAAGATAAATAATAGTTAAATGATGGTTAAAACGATGGTTAGATTCAGTTAATTATACACATAAAAATAACCGACCATAAAACCTGATTATAAAAAACTATAATCGGTTAACCAAACCATCATTTTTTCGAGTTGATTCAATTATTTAGATTTGATTGCTTATTCACTTATTTTTTTTAAACAACAATTCAATCTACTGCTAATTCAACATTATTGTTCGTCAAGTAACTTGACCCTCGACCTTTCAAAAAGAGCCATCATTTTACACACCTAAACACCACTAGGTTAAAGACACTTTAATATTCGATTGTTTCTTAGCTTCCTATTTACCATAACCTAAATCAAAAAGGAACCTAAACTAATACCATCAGTTAACTAATCTTCTATAAATATTACTTAATAATTTAAAATTTTGTAACTTTGAATGAGAAAATTTATAATCTCCTTCTAGAAATGTAGAAAAATATTAGAAATTAAAGTAATAAATAAATCCAGAAGGCATCTGAATATAAAGTTTATATGCAAGTTGGTCCTCCTACTTGCGTCCTCCCAAATAAATTCAAACTAATTGCATGCAACTTGCAACCCCCATTCTCAATTTTCACGCATATATCACGTCTATATATACAAATATTTATTATTTTGTATCATAACATATCATCATATATCAAACTTCACCTCAAACAAAAACAAGAAATAATGTGCACCTCCATTCAAATCCAACCCCACCCCAAATCCACTATGAACCACCAGTCTAGCACCATCGGTACTGGTATCGTATGCAAACAACTTCCACAATTAATTAACATCCCGGAAGACCTACACCGCCACCACACGCACCGCGTGTCCCACAACCAATGCTTCTCCACAATAATTCAACCCATCGCCGCCCCCCTTGAGACCGTGTGGTCCCTAGTCCGTCGTTTCGACAACCCGCAAGCCTACAAACACTTTCTAAAAAGTTGTGATCTCATTGTTGGAGACGGTGACGTGGGGTCCGTGAGGGAGGTTCAAGTAGTGTCTGGGTTACCCGCCGCATCCAGCATGGAGCGGCTTGACATCCTTGACGACGAACGCCATGTCATCAGCTTCAGTGTTGTTGGTGGGGACCACCGGTTGAAAAACTACCGGTCCGTTACCACCCTCCACTCGTCACCCACGTGTGGCGGGGGTACGGTGGTTGTGGAGTCGTATGTTGTGGACGTGCCGCCGGAGAACACCAAGGAAGAAACGTGTGTGTTTGTGGAGACTATTGTACGATGCAACTTGTTGTCACTGAAACAACTCGCCCAGGATTTGGTTCATGATAAATAA

>transcript:mRNA:HanXRQr2_Chr10g0429361

CATTTCACTTTGACCCCTTTGTATTTACACTTGTTATTATTCACTACCATATATCTACATACAAACAATTTTTCAAGATCTTATACTCACTCAATATTTGAGTTTTATGACCGTGTGGTTAGGTGATTTTAGTTTGTTGAAAGCATTGCCGGCAATGAAGCATGGTTACAATTTGAGTTTATATAACATATATATTGATTTCTTGAGAAAATATTCTGTAACTATTTGAAGTTCACTAAGAAGGCTCGTATAAACCGAGAGCAATGAGTGAGTGGGGCTTTTATAGGGCTGGCTCTGAGGGTGTGCTGGGTGGGCAATCATATTGTCTTAAAACCCAATAATCATTTAAAACACTTAAAAATTAACCTATTTATCAAATAAGTCTTCATAGCAGCAGTTCTAAAGTTGATTAACTGAAAAACTGAAGGGATGTGGACGGTGAGACGATCACTAGTCGACTCACTCTATCCGTGGATAACCCACTGTTGGGCATAAGACAATCGTCCACTATCGATGCATTTTGTTTTTATATTTAGGTTCCTATTATAAGGGCCAGTTTTTTTCTAGTCACACACCGAAATTTAAGAGCTAACCCTATGCTTCTAGATGGTCCGATGAAGGCGACATTGGCTGTTTGGTATGTAGTCTAATGAAGGTTAATGGACAAAAAGACGCAAAGGACAGTGGTCGCTATTAGGAAGGACGATGGTCACTATTGATAAAGGACAATGGTTATAATGACCATAGATGTATATGAATGCTATAGCAAAATAATCCAAGAAGAGTTATGAAGTTAAGTCAAAATGAATTGAACTTTTAAAACAATACATTAATAAATATAGGGTGGGATCTGGTACAAATAAAATCAAACTACTAAATGTACAAATAACTAGAGTATTTACACTACAAAAATGAAGTGGAGTAAGTCATTTTTGTTCCAAAAATTATGATAATTAATTTATTTATTTGTATGTATAGGTTATTGAATTATTTATATTTGATCCTAACATTAATAAATGTTAAGTGATCGGTTCAAAATCGAATTTCGAAAAGTATTAGGGTCATCATGCTAATACATACAAACTCAAGGGTCAAATACAAGTATCCCATTTGCACGTTAACATACAAACAAATAAAAGGCAAGTTGCTAAATAGTGAAAGCAACATGCCATTTCAATAAGGTCAAAAGAGTAATTTCACACGAAATCAAATGCAATATGGCCTTAATTTATGGCTATTAGGGTTCAACAAGTAGCCATATTTTACTCATTCACATGAACCAATACCCCTACACCCTACCATTTATAATCCAGTATCAAGATTTTGTAAACAAATCAACATACAAGTTCGATCTATAACTCTATTCATCTCAATAGATCAAAATGCTTTCAAACCCACAAAACTCACCATCTTTCTTACTAGACAGAATCAACACAACCACCGTAACCGGCGGCTCTGGCGCCTGTGGAAACACCATCTTACAACCCACCACCACTATTCCGATACCAGACTCCGTCGCACGCCACCACATCCACCCGGTAAGTCCCAACCAGTGCTGCTCCGCCGTGATCCAACACATCAACGCCCCAATCTCCGACGTATGGTCCGTCATCCGCCGCTTCGACAACCCACAAGCCTACAAACACTTTGTCAAAAGCTGCCATGTCATCAACGGAGATGGTAACGTCGGAACCCTAAGAGAAGTTCATGTCATCTCCGGCCTGCCCGCTGCCCGTTCTACTGAACGTCTTGAGATTTTAGATGATGAACAACATGTTATAAGTTTTAGTGTTGTTGATGGTGATCACCGGCTTGCTAACTACCGGTCTGTTACTACTCTTCACCCTACACCGGCCGGTGACGGAACGGTGGTTGTTGAGTCTTATGTTGTCGATATACCACCGGGGAATACTAAGGAAGAAGCGTGTGTGTTTGTAGACACTATTGTGAAATGTAACTTGCAGTCGTTGGCTCAGATCTCGGAGAATAAACTCCGGTGA

>transcript:mRNA:HanXRQr2_Chr12g0530941

TATGTAGTTGAGGAGGGTGAAAGTCAAGGTATAAATCTTGATCATGCACCTTTCAGTTTTGCATAAGTGCTACTGAAACATTTGTGATGAATCAACGTGGATTTTAAGTTTTGATACTTTATTTAAATAAAAAGTTTAAACCAGAAATTGTAAAATTACTTATTAGTAACTTTTAAAGTGTTCTGCTCGCCTAATTTTGGATATATGTAATTAAGAAACACAATGACGTTCGAAGCGCCGCCGCAACGCGCGGCTTCATTTACTAGTTTATAACAATTGTCAACTTGCGCGAAAACTGACTTTGACTTTTTTATATGTTTCTATTACACATCTTATGCGTTTTTATCCCTATAGTCTTGTTTTTTTTCATATAACTTCCGTTCATTTGTTTTTTATAAAGACTAAATACATGGTTGTGTTAAATTTTTTTCTTCGTTTCAATTTTGAAATGTTTATATTTAGAGTCGTTTACAACTTTCCATCAATAATATATATTAAAGAAATGGTAGGGATATGGTAAAAACTGTTTAAAATGTGAGAAGGGTAAGAAGTGTTTTAAACCATTGGATATTTGATCTAATGGTTAAGATCAATAGGGTATAAAAATGTAAATTGTGTTTTAATTAGAGGGACCTTATGTAAAATTGAAGGGCAATAGTGTCTTTTTCAATGTTTGAAATATGGTAACCGTAACCTACACAACCAAAAACACATACATTCACTCATTTTTCCTTAAATATCAGAATTAAGATGAGGGACACTTGTCATTCCCAAATTATTTCTTACACTTCTTACAAAAGATGGACTTTGTACAGGATCCTCTACCATTAAACAAATAACTTGAATTTTCTCTTTTAGTTGTTTTATCATATATTAGAGAAATAATTTAGATTTTCTCTTTTAGTTGCTTTATCAAGTGTCGTTACGTTACTGTATCAAAACAAAACAAATAAACACCAACCTAATCCTCGCCGTATAGCGTGGGGGACTTTTGCTAGTTATTACATATAAATAGAGGTTACCAATCACGTTATTTTCAAGTTTCACATTTTTTACCACACAAGTTTTTTTAATCAACTGTTTAGTTATTCGAAAATAATTGAAAGAGATAAGTGACCCCTTTATAAACAGAAGGGACAGGTACCCATGCGCAGCGTCACATTATACCCTGGCTGGCCCCACAATATAGGTAATATTGGTAAATACCAGATTTTAACCAACACTTTCTATTTGTGTATATCTATTAAACTTTCAACTTCAATCTTATCTAATTATTATATCACATAGCCTCCTCCTCCACTTACTCTCTCTCTAGATCCA

ACCCATTCCTTTATTTCTGTCCGAGTTGATCCTGATTCGACATTAAAAGTATATTGATTTTTTTCCGATAACGGAAAAAAATGGAAACAACCCACCACCTCCACCCTCCACCAGGCGTGACCGAACAAGAGTTCGACACATTAAAGAGTTCCATAAAAGAATTCCACACCTACATCCACACGCGAGCCCAATGTTCATCTCTACTAGCGCAACACATACACACCTCATCCGACATAGTCTGGTCAATAATACGCCAATTCGACAAGCCACAAGTCTACAAACACTTCATCAAAAGCTGTAGCGTGAAAGAAGGCTCTGTTATAGCCGAAGGATGCACGCGTGATGTTATTGTCATATCTGGGCTGCCAGCGGCTACTAGCACGGAGCGGTTAGACCGGCTGGACGATGAGAACCACGTGATGGAGTTTTCCATCATTGGTGGTGAACATAGGTTGAGAAATTACCGTGCTGTCACCACAGTGCACCAAATTAAGCCCAACGAGACGATTGTTTTGGAATCGTATATGGTGGATGTGCCCGAGGGGAACTCGGAGGAAGACACTCGGTTTTTTGCGGATACTGTGGTAAGGTTGAATCTGCAGAAGCTGGCGAATGTCACCGAGGCTATTGCCCGCGGTGCGGAGACTGCTGCTGCGGCTGAGTCGCTTAGTATCAGGTGA

>transcript:mRNA:HanXRQr2_Chr13g0596431

TATATCCCACCAATAGCAAAGCTAAGGTAGGGTCTGAGGAGAGTGAGATGTAGACAACCTTACCTCTACCCCGTAGGAATAGAGAGGCTGATTCCAATGAGACCCCCGGCTCAATTGTAGTTTTGCATCAAGCCTTGGACATGAGGCACATAAAACTCAGCAATTGAGACAAACACCGATTAGTGCATGTACCCCCTTGTCTTTCGGCTATCAACGTCACCACATGATGCATGATTAACCATCCCCCTCTTTTAACGTTATTTTCACAAAATTAGTAAAATAACGTTAAAATTAGTGCATTTTCACTTTTGCCCCCCGAGCGCCCACACATATATACATTATATGAGCAGGGCGTTATAAATTACTATATAAGACAGTAAAATATCATGCTTTCTTGATATTATAATTTCAAAAGGCGTAACTTTTTACTATATAAGACAGTAAAAGATCATACTTTCTTAATATTATAATTTCAAAAGCATAACTTTAGAATTCCCTTTATTTCTTCAAATGTATTAGAGCTGCCTATATCTGTTAATATGGAGCCAACCACTACAAAGTACAATAGTTCAATTGGTTTGGGCAAGGTCTGTCTAGATTCCACTCTCCCAGATCCTATAAGTAGGTTTGCTATTTGTGGGATTTACTTGGTATGGTTGTTGTTGTTGTTGGTTTGGGCACTAACATAAAACCCAATTTTAGCTATTTTTAGGAGAGGGAATGGTGCGAAGGTGAGAGAGGGGAAGAATTAATTTAATTTAATTTTTTTTTTGTGTGAAAAAACGCTAAAAACGGGTTATGTGATAGGGGAGAGATAGAATGCGTGTTTTTGGAGCCATTTTTGGTTGTGAGAGAGGCAAAGCCTGGAGAGTTTGGGCCGGTACTTATGTGGTCTATCTTTGTTAGATCACCGTTTGTGAGTGCTCTTAGTTTACATTTATAATATAAACTTTTAAGTTAAATTAGATCGTATGCATTTTTTAATCACGGTTGAGTTAATAAAAGTAAAGCATTTGAAGTATGGACGGATCTATGCGCTTGCTTTACCATCACCCACATTCACCACTAGCCGTCATTAAAAAAAAAACAACTTAGTGGGTGAAACTGCTTTGACTCAACGTGCCACTGTTCTGACATGAACCCATTTGACCAATTCTTATTTCATGCTTGTTTAACCCTATTTAACCTGAACCCATTTCACCATAAAATAGTTAAAATATTAGTCATGAAAGAAACCGAAAACTATTTTTTTAAAATAGAAAATAAATAAAACAAAAAAAAAACTATCCTTTTCAGAATCACAAGAACTTCATTACAACCTTTTTTTTCTGCCGACACTTCCATTTCCACGTGATCTTCTTCCAACTTCGATTCGTCCTTTTCTCTTCATATCAACCAATGCCCCACACATTGATTCATTCATTCATTTTCCTCACAAAATGGACCAAAACTCAATCCCAGAAGGCCTCACACCAGAAGAATACTCACAACTCCAACTCTTAATCAATGCACACCACATGTTCGACAAAATGCCCAACACATGCACGTCCCTCATAACCCAACACATCGACGCACCCGCACGCGTCGTTTGGCCACTCGTACGCAGCTTCGACAACCCACAACGCTACAAGCATTTCATCAAGAGTTGCAACATGTCTGGGGACGGTGGAGTTGGCAGCATACGCGAAGTGACAGTCATGTCGGGTCTCCCAGCCTCTACTAGCACTGAGCGGCTTGAGGTTCTGGACGACGAGAAGCATATATTGAGTTTTAGAGTTCTGGGTGGGGAGCATAGGTTGAGTAACTATCTTTCTGTTACGTCGGTTAATGAGTTTAAGAAAGGCGAAAAGGTGTACACCGTTGTTTTGGAATCGTATATTGTTGATGTGCCTGTTGGGAACACGGTTGAGGATACGAAGATGTTTACGGATACTGTTGTTAAAATGAATCTACAGAAGTTGGGTCTTGTCGCGTTGTCTTGTTTGCGCGGAAATGAATGA

>transcript:mRNA:HanXRQr2_Chr14g0666021

GTTAAAAACAATATAATATTTAATCGATATAATATATTTAAAGAGCCGATTTTTAAAATAGTTGATTTAATCGATATAAAACAAAAACTTTTTATAGTGTAAAAAACAAAAAAAAAACAAAACTTTTTTAAAAACCAAAAAAGAACGATTTTTAAAATAGTTGATTTTTAAAATAGTTGATTTTTAAAATAGTTTCTTTTAAAAAACAAAAAAAGAAAAAAAACTTTTTTTAAAACCAAAAAAAAAATTCAACAAAAAAACACATCTTCAAAAACTATCCAAAAAACCATACCAAAACCCACCAAAACACCCCTCATTTCAGCCAAAAAAAAAAAAAAAACCAAACATGGACGCCTCGTATAGGGCTATACCCTTCGTATTGGGTTCCTTAAACATCGTGCCTGACACCCTCTGAGACCCCATCGAATTCAGTGCATGTACGCCTCGTATAGAGCTATACGAGGCGTCTAGGTTTCAAAAAGTGTGAAAATAGGCGGACAAGGTGTCCGGATAGTCTGAGCCTTTTTTAATTAATCTACGGCATGTTAATACATTATTCACTAACGAGATGCATAGGACCATGACCACCCCGAGGAATCCATTTCATGTTAACTCAAATCACTCGTTCGTTAGCGCTTGCGATTGGTCATGAGCTGTAACTCTAATGTCCATGGTAACAGTGTTGCTTTTCTCTAGTCAGACAATCAAACCTTCAAACACAAAACAAGCAAGACAATCAAACCTTCAAACACAAAACAAGCAAGTTCTGGATTAAAAAAAAGCGTTAAAATAACAAATATTTCCATTTCCCCACATTTCTTTGGTTAAGAGATCACATCACATGAAAGGTTTAAAATGTTTTTACATATAACATGGACAATATTGTGTGTATATATTATATTCCCGGACAAGGTTTTGGTCAAAAAGCGCCAGATAGTCGGTGGTGCCGAAGATTCATGCACGCTTCATAGAATTACTAACTTTCCCTTATAATCCATCCCTTCCCACCATCTAAAAACATAAAATTACTTAAATTTAAATAACCTTATATTTATTTTTACTTTTTTGTACGGCAAATACTTTAATTTTAAAAGTCCAAATTGTAAACTCTTACTATTTTATATTCATAAAAAATATTATATATCTTATTTTCATACATCCTTTTCCAATTAAATAAACTGCAAATAGGAAAAAAAGATTATGCAAGTTGGTTGCTACCACTTGCATGTCCGGCGTAATTCCTGTGTTTCTTCGTCTTTAAACTTTACAACTTGCTCCCATACCTCCATTCCTGTATCTATATCTATATATCTATATCTATATCTATATATAATCTTCACATCACAATCTACACAAACCATCATCTCAAAAAATACAAGATGCCTTCTTCCGTTCAAGTTCACAGAATCCACCCAACAACCACCACCACAACCACCACCACCACCGTCAACAACCACAAACAACCATCAACAACAACCATATGGAGGGTCCCATCATCTATTAACATCCCAGAAGATCTTAACCACCACCACACCCATGCAGTTGGGCCCCACCAGTGCTCCTCCGCCGTCGTACAAACCATCGCCGCCCCCATCGCCGCCGTGTGGTCCGTTGTCAGACGGTTCGACAAGCCGCAGGTTTACAAACACTTTCTTAAAAGCTGTCACGTGATTCTTGGTGACGGTGATGTGGGTACTCTGAGAGAAGTCCACGTGGTGTCAGGACTGCCAGCTGGATCCAGCACAGAGCGGTTAGAGATTCTGGATGATGATCGACATGTCATGAGTTTTAGTGTTGTCGGTGGTGACCATCGGCTTAATAATTACCGGTCTGTTACGACTCTTCACGCGTCACCGACATGTGACGGTAGCACTGTTGTTGTGGAGTCTTATGTTGTTGATGTGCCGCCGGAGAATACTAAGGAGGAAACGTGTGTGTTCGTGGATACTATTGTACGATGTAACCTGATTTCGTTGAAGCAAATCGCTGAAAGTAAATAA

>transcript:mRNA:HanXRQr2_Chr15g0671331

AACCCGTTTGGCTCAAGGATCCAACCCAGGTTTCCCTGGGTCTCCTATCATTGCCCACCAATGTCTCACTCTGCACCAAGTGGGAGTCGAACTTGCATCTCTCAAGAGAAATGCAAGCCCTCCACCACTTGATCATAACAATAAGTGAAGTCAAATCATAGGATTAATTTTTCCTTCATATTTATTTGTTCTTTAAATTATAATATTTATATTGGTTTTTTTAACGGTGAACATCATGTATAAGGTTATCACATTCATTAAATCGGAGAGCCCCGTATTGCCCCACTCTAGTTTCAAAGTTTGGCTTTTTTGGGCGTTACCTCCGGGAAACCATACCGCGGGCTGCCACTTGACAGTCGTTGTGTCATCATAAGAGCAGAACTCTCTGGCGTAACACACGGTGGGACGAACACCCGATTGGGCTCGGGAATCGAACTGACAACCTCACACAACGGCAAGTCCAAATCCTTTATTGCCTATATCCACCCCAATATGACATAAGTGAGAATTGAACTTGAGTTCTATTGAAAAACCCAAGCCTCAACCTCCTGTAAGATTAAATATATTATGTTTTAATATTTAATATAGTAGCCATTATAATCATTTACATAATATAGATCAAAATATATAACAACCTATTAAATAATTAGTTGGTAATTGTTGATGGACCATATTACTTTTGTTAACTAATTAGGTTTCCCCTTGAGTGCATATATAAGGAGACTTATGTAGAGATTCTAAAGGAGACTTATGTAGAGA

TTCTAAGGTTAGACAAATAACCTAATTCTCATAACATCAAAATCGACCCTCTTCTCCATAACCGATACCATTTCGGTTTTCATCATCACCATCATCAGTTTGCACCCTAAAGAGGAACCAGATCATACTGACAAACATGTTCGAACTCGACGGCATCATCTCTCACTGGATTCTCCTCTGCACTGTCTACTATAACATGTATGTTTTCATGTTTTCCATTATGCTTAAAACAGAACTGATCGAACACCTTCATCTATCTTGTTTGTTTGAATCGAAATTTAATGTTTTAGGGTCACGATATATACAAACTTCAAGGGTCAAATGTAACCATCCAAATTGCACATGAATATTATTATTAATATATAAACAAATTTAGGTCAAGTTGATGAATAGTTAAAGCAAAAGGCCATTTTGACAAGGCCAAAAGTGTAATTTCATATGCAAAAGCCCCATTATTTGTGGCTCTCAACCTTAGGGTTCAACCAAGTAGCCATATTTTACTCATTTTACACCCACCATTTATAACACCTTCATGTTCCAACATTAAGAAACAACAATCAAGATTCTTCAACTCATCAACTTTCATCTATATTCATCTTTTAAATCCAACAGATCAAAAAATGCTTTCTAACCCACAAAAGTCACCATCTTTCTTCCTAGACGGAATCAACACAACCACCACCACCACCACTACCGGAAGCTCCGGTAGTTGTGGCACTAACAAGCCACCACCAACCAACACCACTCCGGTACCGGACTCCGTTGCCCGCTACCACATCCACGCCGTGGGTCCAGACCAGTGCTGCTCCGTCGTCATCCAACACATCACCGCCCCAATCTCCGACGTCTGGTCCATCGTCCGCCGCTTCGACAACCCACAAGCCTACAAACACTTTGTCAAAACCTGCCACGTAATCCTCGGAGACGGCAACGTCGGAACCCTAAGAGAAATCCACGTCATCTCCGGCCTGCCCGCTGCCCGTTCCACTGAACGTTTGGAAATCTTAGACGATGAACAACACGTGATCAGTTTCAGCGTCGTTGACGGTGATCACCGGCTTGCTAACTACCGTTCCGTCACCACTCTCCACCCTACACCCGACGGAACCGGTACGGTGGTTGTTGAGTCCTACGTTGTTGACATACCGCCGGGGAATACTAAGGAAGAAACATGCGTGTTTGTTGACACCATCGTGAAATGCAACCTCCAGTCGTTAGCTCAGAAAATCCGGCGAAACTGA

>transcript:mRNA:HanXRQr2_Chr16g0742371

CATTTCCATGGTGTGCATTGTTTGGTTGGTTAGATTATTATCCTCAACCAAAATCGAGGTCATCGATTA

GTCTATTTTATTAACCAATCTGTTTTTAGTTAATCGGTTCGGTGTATTCAGTTAGATTGGCGGTTTTTGGTTCGATTCGTTTAATTTCGGTTAATAACCAAAACCAAATTTGGTCTAAAACTTTTTATTTGAAATACATGAAATTTAGGTATAAATACATGAAATTGATGTACTGGTACATAAAATGGAGGTATATTTACGTAAAATGGGGGTATGTATATAAAATACATGAAATAAAGGTATAAAAGCTAGAAATATATAAAAATATGAATGGTTCGGTTCAGTTAATTTAGATTAAATGTGGGTACAAAAATTCGATAACCAATTTTGGTTAATTCAAGTTTCGGTTAATCAATTTTCAGTTAATTTAGTTTTTTGTTGATTTCGGTTCGGTTTCTGTTAACGATTCAGTTATTACTCACCCTTAGACACTTTTAATCACGTAACATATCATTTTTTTAATTAAAAACTATATCAATACTATACTCAAATTAAAGAAAATAAAATTCTCGTTTATATGGTGTAATTTTAAAAAATACTAACGTACGAAAAAGTTATGACCGTTTAAAAATCGTAAGGGTGAGTTTGTTTTTTATAAGGGGATAAAGTGTAACAACTAACTAATTATCTATAATTTTGTTAAAAATGATTGATTACAGTGTAATATAGATGAGATAATTAAATTGACAGTATTTGATCACTTGACATTCTGTAGCTATTAAACATTCTACTTGTACTTTTTAATCCACATGTCAATACTGACAAAGACAACTATCGAAACGTCAGGAAAAATGAGTAGGTCGTCACCGTACTTTTGCATTTTTTCTAAAAACATTATAGTTTATAAGATATGACAAAAATACGTAAAAGAATTATAAGTTTGTTGTGGAGGGAGAAAGTGTAACTAATTACTCATAATTATGTTAAAACTTAGGGTTTTAAGTGTAATAAGATGGTGAACTAAAATATTATTATTATTTAAAAGACTAATTACCCTTATTTTAAGAGTATTTATAAATTAAGAGAATTTTTTTTTTATTTTTTAGGTATTTTAATTATCTTTTCTCATAATATATAAATACTTGGCTCTTTGACAACCAATGTGAACTTTTTATGTTTACAACTTTACATATAATAAATTTTCTTTAGAAAGTTGGCATTATAAATGATGAAAAAGAAAAAACAAAGAAACTCAAATGTAAGCGTCCATTAGTGAAAGTTTATGGTCCCTAGTTTGGACCAAGTTGGCCTTATTTTATTAAAATCACATGATCAACTCTCACCTTCCTTCTTCTTCTTCTTCTCACTTTTAAGATCCCACCAAACCATCTCTCTCTCTGTGAAACCACTTGTAGATCCTCATCAGAGACCCATCAACATTGACCATCCAAGTTCCAACCATGCTTTCAAACCCACGAACCACCACTCACCCATTTCCCGACACCATTTCCAGCCTCCACACCCACCCAGTGGGCCACCACCAGTGCTGCTCCGCCGTCATCCAAATCATCAACGCCCCCGTCTCCACCGTCTGGGCCGTCGTCCGCCGCTTCGACAACCCTCAAGCCTACAAAAACTTCGTCAAAAGCTGCCACGTCCTCAACGGCTCCGGCGACGTCGGCACTTTACGTCAAGTCCACGTCATCTCCGGCCTCCCCGCCGCCACCTCCACCGAACGCCTCGACATCCTTGACGACGAACGTCACGTTCTCAGCTTCAGCGTCATCGGTGGCGACCACCGTTTATCGAACTACCGCTCCGTCACCACCCTCCACCCCACTCCGACCGGAACCGGTACGGTTGTTGTTGAGTCTTACGTTGTTGATATTCCTACGGGGAATACAAAAGAAGAAACTGTTGGGTTTGTTGATACGATCGTTAAATGTAACCTTCAGTCCTTGGCTAAAATTGCTGATGACAAGTTGAGTTGA

>transcript:mRNA:HanXRQr2_Chr17g0780891

TTGTACATAGTATTCCTTGCATTTATGTATACCATTTTTTTCCATCTAAATTGAACAATATCTTAAACTTTACAGTCTTGTTAGAGTGGGTCCGTTCGGCTGATGAATAAAATGCCCCTGTTTGGTTAAGAAAAATCTTAACCTACATTGCTTTCGCTTTATAATGTGCAATTGAGATCCTTTACGATAAATTAAATAGAGGTGAAAAAATTAACCGACATAACCGAACAGCTATTAACCGATAACCAATGGTTATGAAAGTTTGTTTAACCGACCAATCGGTTATGGTCACGGTTTTAAAGCTTTGGTTAACCAGTATAACCAAAACCGAACCGGGCTTGTAATGTTAAGTTTGTATAATGGATTTATGTCTTACATTAACCATATAGAAGAGCTTTACTAAGATAAAAGTATGTCAGTAGCAAAATAATCTTGATGTAGATACCCTTTATATTGATGAGAAATTAAGGTGTCATGGTAGAGTTACCTTATTAAATAGAATCTCACATAGGTAGTTTAACCAAAAAGTGCAGTCTCGATTATCATACAAAAAAAGGACAAAGCCAAGTCCAAACCATGTACATCCCTATTTATTTCAAACCTTGATTGGTTAGATCGCCGAAATAACCAAAACCAAACCATGTAAACCGAAGTTCGGTTATGGTTATAGTTAAGGAATTCCATAACCAA

AGCTAGTGGTTATGGTTATAATTAATGTAAAAACCGAACCAAACCTACCCATACACACCACTAAAATTAACCAAGAGACTTAGGGGAGGGGGGGTGGTTCACTAGTGATAGAATCTATCACTCACAAGCACCAATCAAGTTCCGCCATGTCATCGACCATTTTTTCATCACTCACAACCTTTTTTAGTGGGGGTGGTCATCACTCACCACCACACCCAACAATTTCCCTCAACCAACAATTACACTCACAATACCAAACAATAACGCGTGATAAAAATAACGAAAATCGGATTTAGTGGAAATATAACGCGTTGAACTTTTCCCGGCGGTGGCCCATCACGCGTTGAACTTCTTTCCGTGATAGTATAACGTTCCCACCCCGCCACCTCTAAGGGAGCTTGCACAAGTTGTAAGCAATCCCTTTGTTAATGGAGCTTATACAATGTTAAAAGAAAAAAATTATCAGCAAGCAAGCAGGGACTAATCAGACAATTTGGAGTAGTACAGGGGCCATACACATACTACAACTAATTTAATATCACATGAAAATGGTGAAGTGAGAGAAACCAGATGTGGATGAGAAAGAACCAACGAAAACCCCAATTGCTGCTGCTGCTGATGTGATTATTCTCTCTCGAATTCCTCAACGAATCCACCGGACTTTCAACACTAAATCTATCGCACCGATCAAATTCAGTCACAGATTGTTCGACGATCGTTACTCTTATCCATAACTACCGATGGTGATGAACGGACACGATGACGCTACAGATTCGTCGGAGTACAGCCACATTAGGAGACACCATAAGCATGAAGTTAGAGCCAATCAGTGCACTTCATCTCTCGTTAAACATATCAAAGCGCCTGTTCATCTTGTATGGTCTTTGGTGAGGAGGTTTGATCAACCACAAAAGTACAAACCCTTTGTGAGCGGGTGTTACGTGCAAGGTAACCTTGGAATCGGGAGTGTGAGAGAAGTATGTGTTCGATCTGGGCTTCCAGCTACCACGAGTATGGAGAGGTTAGAGCTTCTCGACGAAGAGGAACATATCTTGGGCATAAAAATAGTCGGCGGTGACCACAGGCTTCGGAACTACTCGTCAATTATGACACTTCATCCAGAGATTATCGACGGGAGGCCAGGGACTTTGGTGATTGAATCGTTCGTGGTGGACGTGCCAGATGGCAACACGAAAGATGAGACGTGCTACTTTGTGGAGGCTCTCATTAAATGCAACCTTAAGTCGTTAGCTGATGTATCGGAGCGTTTGGCTGTGCAGGACAGAACAGAACCCATAGTTGGCGTGTGA

**Additional File 4. mRNA sequences of *HaPYL*s for qRT-PCR**

>XM_022113537.2 (HaPYL8a)

TGAAGAAACAAGATGTGGATGAGAAGAACCAAAAAGCAGTTAACGCTCAAGAAACCCCCAATTGGTCACCTGATTATTCCTTCTCGAAACTCTCAACCGACTTTGTGCCTTTATTAACCGCACCGATCAAATTCCGGTCACAGTTCGCCGTTGCAGTTACTCATAATAATCACCGATGGCGCTCACCGGAGACGACGACGCAACAGATTCGTCGGAGAACAGTTACATTAGAAAACACCATAAGCATGAGGTTAGAGACAATCAGTGCAGTTCATCTCTTGTTAAACACATTAAAGCGCCTGTACACCTTGTATGGTCTTTAGTAAGGAGGTTTGATCAACCACAAAAGTACAAACCCTTTGTCAGTGGGTGTAATGTGCAAGGTGACCTTGAAATCGGGAGCGTGAGAGAAGTGTGTGTTCGATCTGGGCTTCCCGCTACCACAAGTATGGAGCGGTTAGAGCTTCTTGACGAAGAAGAACATATATTGGGCATAAAAATCGTAGGTGGTGACCACAGGCTGCGGAACTACTCGTCGATTATCACGCTTCATCCGGAGATTTTAGACGGGAGGCCAGGTACTTTGGTGATTGAATCGTTTGTTGTAGATGTGCCGGATGGTAACACGAAAGATGAGACATGCTACTTTGTGGAGGCCCTCATTAAGTGCAACCTTAAGTCATTGGCTGATGTGTCTGAGCGTTTATCGGTGCAAGACCGAACAGAGTCCATAGTTGGAGCGTGATTTAGTTGGTAGTAGACTGGATAATATTCCATCTTTGTGCATATTAGAGACTTTTATTCCTGAATGGACAAGTACTATGATTGTGTTTCTTACACTATTCTTGGTAGCATTTAACATTTTCTATTA

>XM_022113657.2 (HaPYR1a)

TAAACTAAACAAATCTAGTCCTTATCTATCCCCCACACCCTCTTTCTCTCTCCTCCTCACTCACCCGGATCACCCAACGTCAACAAACATGGAGCAAGCCGGCACCTCCACACCGGAACACCAAAACCCACCACCACAAACCACCACAACTACCCACCACCTCTCCCTCCCACCAGGCTTAACCGAACACGAGTTCAACCAACTCAAAACCTTCGTATCCAACTTCCACACCTACCATCTCTCACCCTCCCAATGTTCTTCCCTACTCGCCCAACACATCCACGCGCCGGTCGACGTCGTCTGGTCCGTCGTCCGCCGGTTCGACAAACCCCAAACTTACAAACACTTCATCAAAAGCTGCACCGTCGGTGAAAACTTCAAAATGGAAGTGGGGTGCACGCGCGATGTCAACGTCATCTCCGGCTTACCGGCGGCCACTAGCACCGAGCGGTTAGACTTGTTGGATGATGACAACCACGTGATGGCGTTCACTATTATTGGTGGTGAACACCGGTTGAGAAACTATCATGCTGTCACGACCGTTCACGAGGTTGTTACAGAGAATGATGATTCGGTTACGGTTGTTTTGGAGTCTTACGTTGTTGATGTGCCTGAGGGGAATACTGAGGAGGATACGAGGTTGTTTGCTGATACTGTTGTGAAGTTGAATCTGCAGAAACTGGCGGCGGTGACGGAAGCGATGGCGGTTGACGGTGGCGCGTCTATTAAGAATCTGAATCTCAGGTGATGCTGGTGTTAATTGGTTATGGTTTTTAATTCCACAGCCCCAAAAAAATAAATAAATAAGAAAGAAAAAGAAAATGGAAAAAAGTCCAGCCACACTTTATCATTTTTTTTTATTTTCATTGAAATCAAAAATTTGTGAGTTTTTTTTTTTTATTTAATGTTTGTATGGAAGTTACAAAATCACCCTTTGTCTGTAATGATGGGCTGCCACTTGCCACTTGCCACTGTGAATTGTATATTGGGGGCATTTTCGTCATTGAAGTGTTTAATTTGTGAATGTGTTTTTTA

>XM_022113987.2 (HaPYL9b)

ATGGAGACAATCACCCAAGGATAATGCCAATTGGGTGGAATCACTTTACATGATTTCGTTTGTTTGTTAGCGATTTTTATAAAATTATACGCACATGCACTATTCCCACCACTAATTCCCCATCCATCCTTCTTCTTCTTCTTCAAAAGTCAATAACTTTTCGATTCATTTCATTTCTAGGGTTCCAGTTTCTCCTCTAATCGCACAACAAATTGATCATCAATGGATGCAGCTCAGTATATACGAAGACACCACAGGCATAATCCCGGCCGTCAACAGTGCGCTTCTGCCGTCGTTAAACACATCAAAGCTCCCGTTGATATCGTTTGGTCATTAGTTAGGAGGTTTGATCAACCTCAAAAGTACAAGCCATTTGTTAGCAGATGCACGATGCGTGGGGGTCTTAACATCGGGAGTGTTCGAGAGGTGGATGTCAAGTCAGGGCTTCCAGCCACTACCAGCACAGAGAGGCTGGAGCTTCTTGATGACACTGAGCATATTCTCGGCATCAAAATTGTTGGCGGTGACCATAGGCTAAAGAACTATTCGTCGATCCTAACTGTCCATCCAGAGGTAATTGAAGGAAGATCCGGGACGTTAGTCATAGAGTCATTTGTGGTGGACATACCCAATGGCAATACTCAAGATGAAACATGTTACTTTGTGAAAGCCCTAATTAACTGCAACCTTAAATCTCTTTCTGATATCTCCGAAAGAATGGCTGTTCAAACTCAGATGTGAAAGGACGAAAGTGCCCTCGCCCCTCATGGTCGTGCGATTATCAATCAAACTTTCTTCTCTTCATTTGGCTTGCTTTTAAGTTGATATGTATAATTTGGTGATAGGCACTAGTAGTAGCAGCTCATTGTTTGCAGGATTGCTCATAACTGCAAACATTTGGTAGCGGTTTTATATATATTATGGTTCTAAATGTCACTATGTGTTAGCTTCATGTGTAATTTTCTTAATGATATATATAGTATTGTCTAAAAATATTAGATATAATGTTTTTTT

>XM_022116937.2 (HaPYL2b)

CATTGTCCACCAAACTCTCTCCTTTCACTTTCAAAACTCATCACAAAAAAAAGATAATAAATAAAAAAAAAATTATTACCTCATCAAATCTCCTACACCCCCAACCCAATGATCCAATAAATCTAAGACCTTCCAAACCCTCAAAGCTAATCACTCTTCTAGCTCTCCAAATGGACAGAAACACCAACTCAAACTCAAACTCAATCCCACAAGGCCTAACCCAAGAAGAATACACTCAACTCCAACCCTTAATAAATGCATACCACATGTTCGACAAAATGCCCAACACATGCACCTCACTAATAACTCAACGCATCGAGGCGCCCGCCCACGTGGTCTGGGCTTTGGTACGGCGGTTCGACAACCCACAAAGGTACAAGCATTTCATCAAGAGTTGCAGCATGATCGGTGACGGAGGTGTTGGGAGTATCCGTGAAGTTAACGTTATCTCGGGCATCCCGGCGTCCACGAGCACCGAAAGATTGGAGCTGGTGGACGAGGAGAGGCATATTTTGAGTTTTAGAGTTGTGGGTGGTGAGCACAGGTTGAATAACTATCTTTCGGTTACTTCGGTTAATGAGTTTAGTAAGGAGGAAAAGGTTTACACAATTGTGTTGGAGTCTTATGTTGTTGATGTTCCTGAAGGGAATACGGTAGATGATACCAAGATGTTTACGGATACGGTTGTCAAGTTGAATTTGCAGAAGTTGGGTGTGGTGGCTATGGGCTGTCTGCATGGTGGTGAATCATAGTGGTACTGGTGGTGGTGGTGGTCAGGAAGAAGATAATGGTTGGTGACGTTGGGAGACTTGTGAGCAAGGCCAAGACTCATGGGAAACATGTGAACATGAAGCTACTTCTTTGATTCGTGGTTTGGGTGTTCCGGTTCCACTCGTTTTGACTTTTTTTAAGTCAAATTGCTAACCACGATTTTGAAGAACGAAAATTCAAAAATGTCCAATAACAATCTTGTTTCACAGTTTGAA

>XM_022118303.2 (HaPYL4c)

TTCCTCTTTAAACTTTTCCACCATCACAACTTGCAAGTTGCAACATTGCCTCCCATTCCTGACGTTATCTCCATCTATATATACACATATATATCTATTATTCACCATCTACACGCTACAAAAAAAGGTTTAAACTCAAGACTGAATAATTATTATCAAATATGCCTTCTCCCATTCAGATTCAACGAATCCACCCTACAACCACCACACCCACCACCACCACCACCGTCAACCACCACAAACAACCACTCCCCACAACCACAATATGGAGAGTACCATCATCTATATGCATCCCAGAAGACCTTATGCACCACCACACTCATGTCGTTGGTCCAAACAGATGCTCATCCGTAGTTGTCCAGACAATATCCGCACCAGTGGATATTGTCTGGTCTGTCGTCCGTAGGTTTGACAACCCGCAGGCGTACAAACACTTTCTCAAAAGTTGTCACGTGATTCTTGGTGATGGTGATGTCGGCAGTTTAAGAAAGGTACATGTTGTGTCTGGATTGCCAGCTGGATCCAGTACGGAGCGGTTGGAAATACTTGATGATGAGCGGCATGTTATGAGTTTTAGCGTTGTCGGTGGTGACCATCGGTTGAATAATTACCGATCTGTCACCACGCTGCACCCGTCGCAGTGCGACGGATGCACTGTTGTGGTGGAGTCGTATGTGGTGGATGTGCCTGCTGAGAATACTAAGGAGGAGACGTGTGTGTTTGTTGATACTATTGTTAGATGTAACATGATTTCGTTGAAGCAGATTGCGGAAAATCTGGTGAAGAAGTAGTGGTACATAATTTACATATGTTGTATTGACCTTTTGTTTATTGATCTGATGGTTAATCTTTGGTTCAGGGTTTAATTTGATTGATCTAGTATGTATGTAGAGATTGAGAAAACAAAATTTGGAAATTATTCTTTTGGTTTTGTTTTTGTTTTTGTTTTTGGTTTTGGTTTGTAGTTTGTGGTTGTTCATGTGAATTTCTTTGACTA

>XM_022118530.2 (HaPYL8b)

AACACGGTGAGGAAACGACGGAGTACGGACCTGATGACCCGATGAAGAAGCCCCCCGCCCAGCTCTCCTCTGAGTCACACAAACGATTCTTTTGTTTCAGACATCATCAGTGATCGGAAAACACAAGACGATGAGCAGCTGCCGTGATAATAATAATAATATTAGCAGTTTGGAGTCGGAATACATTAAAAAACATCACAGGCACGAGAATATTGCCGATAATCAATGCACCTCCGTTCTGCTCAAACACATCAAGGCCCCTGTTCATCTCGTTTGGTCATTGGTAAGGAGGTTTGATGAGCCGCAAAAGTATAAGCCATTTGTGAGCAGGTGTGTTGCTCAGGGCAATCTTGAGATTGGTAGTCTTAGAGAGGTTGATGTTAAGTCTGGTCTTCCTGCTACTACCAGTACCGAGAGGTTGGAACTCCTTGATGATGATCAGCATATCTTTAGCATTCGGATTATTGGTGGTGATCACAGACTCAGGAACTACTCATCGATTATCTCAGTCCACCCAGAAATTATTGAAGGGCGTCCAGGGACACTAGTGGTGGAATCATTTGTGGTTGATGTGCCAGAAGGGAATACCAAGGATGAAACATGCTACTTTGTTCAAGCATTGATCAAGTGCAATCTAAAATCTCTTGCTGACGTGTCTGAACGGCTTGCAGTCCAAGACAGGACTGAGCCCATTGACCGAATGTAGATGTGAGACCACGGATGAAGCTTCTTTTAAGGCTGTTGGCTTTTGGTAACGAACTGGTACCAGCTGTTCCTCCACTTGTGTTTTATGTCATTAGTTTCGGTGTCATGTTGGACTGTCTGTCTCTAGACTCAAAAACCACAGCTGTATAAGATTCAAATGCTAAATGGTAAAGTCAATCGATGCAAAAAAAAAAAAAAAAAAAAAAAAAAAAAAAGATTTTGTTTTCGAGGTTTCAGCTTCGCTGGTCTAGTTGTGAATAAATATGGTGAAAACAAGTATCATATGTATTTACTTATGTATTTACCATATTTTTTTAGTCT

>XM_022120093.2 (HaPYL2d)

CAACCTCTCTCCTCACTTTCAAATCTCATTAACTACCTTTTGTACATCTTCATACTCATTTCCATGTGATCTTATTGTCAACTCATCTCCACCCCCCCCCCCCAAAACACACACACACACACAAAACCCTTAAATCCATTCCTCCACTTCTCAAAATGAACACAAACCCAATCCCACAAGGCCTAACCCAAGATGAATACTCCAACCTCCAACCCATCATCACCACCCACCACCTCTTCACCAAAACACCAAACACATGCACCTCACTCATCACCCAACGCATCAACGCCCCAGCCCACATAGTCTGGTCCTTAGTACGCCGGTTCGACAACCCACAAAGATACAAACACTTCATCAAAAGCTGCACCATGACTGGTGACGGTGGTGTGGGCAGTATCCGGGAAGTTACGGTCATCTCCGGCCTCCCGGCGTCTACTAGCACCGAAAGACTAGAACTAGTAGACGATGAGAAGCATATCTTGAGCTTTAGAGTTGTGGGTGGTGAACACCGGTTGAATAACTATTGTTCGGTCACTTCGGTTAATGAGTTTAGTGAAGGTGATAAGGTTTACACCATTGTGTTGGAGTCTTATGTTGTTGATGTTCCTCAAGGGAATACGGTGGAGGATACTAAGATGTTTACGGATACGGTTGTGAAGTTGAATCTTCACAAGTTGGGTGCGGTGGCTGTTGGTTGCTTGCATGGTGATGGGTGACGGCAGTGGCGGATCTATCCTAAAAAAAACTTATTTTGTATCAGGGGTGTCTTTTGTAGAAAACCAAAAAAAATAAAAATTTTACATGCACTCTGTAGACGGAGTTAAAGAGTATTTTTACATTACGAATACGAGGTTGAGCTGTAGCCCATGGTACCCCTACTATAACACTACCTCCGCCACTGGGTGATGATGATGGGTGGCGGGTGACGGTGGTGGAGGCGGAAGACTTGGTGTGATGTGATTGTGATGGAGTGTTGCTTTTAAGTAGTGAAAATGTAAGTTTTTTTTTACTCAAATAAGTTTTTGAGGTTAGAATTGGAGTGAAAATGTAAGTTTCTTTTTTACTCAAAATAAGTTTTTGAGGTTAGAATTGGGTATTTGCCCAAAGTTGTTTTTAGGTTTTTATTATTTATTGAATTCAATAG

>XM_022121546.2 (HaPYL4d)

ATAACATATCATCATATATCAAACTTCACCTCAAACAAAAACAAGAAATAATGTGCACCTCCATTCAAATCCAACCCCACCCCAAATCCACTATGAACCACCAGTCTAGCACCATCGGTACTGGTATCGTATGCAAACAACTTCCACAATTAATTAACATCCCGGAAGACCTACACCGCCACCACACGCACCGCGTGTCCCACAACCAATGCTTCTCCACAATAATTCAACCCATCGCCGCCCCCCTTGAGACCGTGTGGTCCCTAGTCCGTCGTTTCGACAACCCGCAAGCCTACAAACACTTTCTAAAAAGTTGTGATCTCATTGTTGGAGACGGTGACGTGGGGTCCGTGAGGGAGGTTCAAGTAGTGTCTGGGTTACCCGCCGCATCCAGCATGGAGCGGCTTGACATCCTTGACGACGAACGCCATGTCATCAGCTTCAGTGTTGTTGGTGGGGACCACCGGTTGAAAAACTACCGGTCCGTTACCACCCTCCACTCGTCACCCACGTGTGGCGGGGGTACGGTGGTTGTGGAGTCGTATGTTGTGGACGTGCCGCCGGAGAACACCAAGGAAGAAACGTGTGTGTTTGTGGAGACTATTGTACGATGCAACTTGTTGTCACTGAAACAACTCGCCCAGGATTTGGTTCATGATAAATAAGGGGAAAAAAGGAAAATTGTGTCTTGTGTTTTAACAGTTAATATTTAGGCAGTTACGTAGTTTTTCATGGGATTTTTTTATCGAGTCGTGGTTATTTCATGGTGAAATGTACTAGATCGATTATTCTCATGTTTAATAAAATATCTTCATTTATTTTTCTATCA

>XM_022126755.2 (HaPYL9c)

GAGAGAAATAGAGAGTGCAGTGGTGGATGAGAAGCAAACACAAACCACCATTTGGCGTCGTCACTTTCTCTCTCTACAATTTTTCTCTCTCTAAAACGTCAACGGCTCAACCGAATTCTGTTACGTTTTCACCGGTGTATCGTTCAATTTCCGGTAGCATCGTTGTAACGGACCGTCAGATTTCAATATTTGATTCTCATCGTTGATTACTATGACGATCTCCGGCACCGGCGACGGCGACAGCGGCGGTACGAGCTTGACAGAGCGTGATTACATCAAGCGGCATCATAAGCATGAGGTTAGAGCGTGCCAGTGCACATCGACGGTTGTTAAACGGATCAAAGCGCCTGTTCATCTGGTATGGTCCCTGGTGAGGAGGTTTGATGAACCACAAAAGTACAAACCCTTTGTGAGCGGGTGCAATGTGCAAGGTGACCTCGAAATTGGAAGTGTTAGACAAGTAAACGTTTGTTCCGGACTTCCTGCCACCACAAGTATAGAACGTTTGGAACTTTTAAACGAAGAAGAACACATCCTAGGCATGCGAATCGTCGGTGGTGATCACAGACTCCAGAATTATTCATCTGTCATTACCCTGCATCCGGATATTATCGAAGGCCGTCCGGGAACTTTGGTTATTGAATCATTCGTAGTGGATGTGCCAGATGGCAACACCATAGATGAAACATGCTACTTTGTTGAGGCCCTTATAAAATGCAACCTTAAGTCACTGGCTAACGTGTCAGAGCGTTTGACCGTGCAAGACCATACAGATCCTATTGTCCACGTGTGATATTGTGATTCTTTTTCACATGATTCATTGTACAAATAGCATTAGTGACCTTCCTTGGAGTAAACTTGTTAGATTTCATTCCTCTATAACCACTTCATGTGTGAACATATTGTCAAATTCGTTTGTCCTCTCGTTTTCTTT

>XM_022127849.2 (HaPYL4a)

TCAACTCTCACCTTCCTTCTTCTTCTTCTTCTCACTTTTAAGATCCCACCAAACCATCTCTCTCTCTGTGAAACCACTTGTAGATCCTCATCAGAGACCCATCAACATTGACCATCCAAGTTCCAACCATGCTTTCAAACCCACGAACCACCACTCACCCATTTCCCGACACCATTTCCAGCCTCCACACCCACCCAGTGGGCCACCACCAGTGCTGCTCCGCCGTCATCCAAATCATCAACGCCCCCGTCTCCACCGTCTGGGCCGTCGTCCGCCGCTTCGACAACCCTCAAGCCTACAAAAACTTCGTCAAAAGCTGCCACGTCCTCAACGGCTCCGGCGACGTCGGCACTTTACGTCAAGTCCACGTCATCTCCGGCCTCCCCGCCGCCACCTCCACCGAACGCCTCGACATCCTTGACGACGAACGTCACGTTCTCAGCTTCAGCGTCATCGGTGGCGACCACCGTTTATCGAACTACCGCTCCGTCACCACCCTCCACCCCACTCCGACCGGAACCGGTACGGTTGTTGTTGAGTCTTACGTTGTTGATATTCCTACGGGGAATACAAAAGAAGAAACTGTTGGGTTTGTTGATACGATCGTTAAATGTAACCTTCAGTCCTTGGCTAAAATTGCTGATGACAAGTTGAGTTGATCGTCAGTCAATATAGAAGAATTGTTATAATTCTTCTTCTTCCATGTTAACTGAGGATCAATTCAACTTATATACCTGTTCTAGCTACTTTCATCTTCTAAAATTTATTTGAATTTGATTATGTCTTTTAAATTTTTCAAGATGATTGGAAAGTTGATCGATTGGTTTAAGAATTATGGAATGATGATATATGACATTACCGTCTTGTTCT

>XM_022132184.2 (HaPYL4f)

TACTCATTCACATGAACCAATACCCCTACACCCTACCATTTATAATCCAGTATCAAGATTTTGTAAACAAATCAACATACAAGTTCGATCTATAACTCTATTCATCTCAATAGATCAAAATGCTTTCAAACCCACAAAACTCACCATCTTTCTTACTAGACAGAATCAACACAACCACCGTAACCGGCGGCTCTGGCGCCTGTGGAAACACCATCTTACAACCCACCACCACTATTCCGATACCAGACTCCGTCGCACGCCACCACATCCACCCGGTAAGTCCCAACCAGTGCTGCTCCGCCGTGATCCAACACATCAACGCCCCAATCTCCGACGTATGGTCCGTCATCCGCCGCTTCGACAACCCACAAGCCTACAAACACTTTGTCAAAAGCTGCCATGTCATCAACGGAGATGGTAACGTCGGAACCCTAAGAGAAGTTCATGTCATCTCCGGCCTGCCCGCTGCCCGTTCTACTGAACGTCTTGAGATTTTAGATGATGAACAACATGTTATAAGTTTTAGTGTTGTTGATGGTGATCACCGGCTTGCTAACTACCGGTCTGTTACTACTCTTCACCCTACACCGGCCGGTGACGGAACGGTGGTTGTTGAGTCTTATGTTGTCGATATACCACCGGGGAATACTAAGGAAGAAGCGTGTGTGTTTGTAGACACTATTGTGAAATGTAACTTGCAGTCGTTGGCTCAGATCTCGGAGAATAAACTCCGGTGAACAACAACTGCTGCAACAACCGTACCAGTAAATCCCACAGATTGGAGAACATGAATTGAGAGTACCCGTCTCGGAACAAAGTACATAAGTTTAATAATTGTGAAGATTCAGTCGATTAAAACCTTCTTTATCTGTTTTGTTGGTGTAGATTTTCATCATCAAAAGTTTTGTGAATCCATGTATGTCACTTTTTTGAAGGTTGTATTGCAGAACATGAGAAAATGTGCTGGATCTTGTTGTTCTTGTTCTTGTTATTGTAAATTGCAATATTTGCAGCTAGTTGGTTTCTTTTTTCCTATTTTATTGATTCGAAAAAAATGAGATTATTATAATTTTTGTATAGTGATTTGCAAGATCTACCATTGAGTTCTTTCATCTATGCAATATTAAACTGTGACATTTTTTTTTAA

>XM_022141537.2 (HaPYR1b)

ACTTCAATCTTATCTAATTATTATATCACATAGCCTCCTCCTCCACTTACTCTCTCTCTAGATCCAACCCATTCCTTTATTTCTGTCCGAGTTGATCCTGATTCGACATTAAAAGTATATTGATTTTTTTCCGATAACGGAAAAAAATGGAAACAACCCACCACCTCCACCCTCCACCAGGCGTGACCGAACAAGAGTTCGACACATTAAAGAGTTCCATAAAAGAATTCCACACCTACATCCACACGCGAGCCCAATGTTCATCTCTACTAGCGCAACACATACACACCTCATCCGACATAGTCTGGTCAATAATACGCCAATTCGACAAGCCACAAGTCTACAAACACTTCATCAAAAGCTGTAGCGTGAAAGAAGGCTCTGTTATAGCCGAAGGATGCACGCGTGATGTTATTGTCATATCTGGGCTGCCAGCGGCTACTAGCACGGAGCGGTTAGACCGGCTGGACGATGAGAACCACGTGATGGAGTTTTCCATCATTGGTGGTGAACATAGGTTGAGAAATTACCGTGCTGTCACCACAGTGCACCAAATTAAGCCCAACGAGACGATTGTTTTGGAATCGTATATGGTGGATGTGCCCGAGGGGAACTCGGAGGAAGACACTCGGTTTTTTGCGGATACTGTGGTAAGGTTGAATCTGCAGAAGCTGGCGAATGTCACCGAGGCTATTGCCCGCGGTGCGGAGACTGCTGCTGCGGCTGAGTCGCTTAGTATCAGGTGATATCAGGTGAAGGTGATATCTGCTACCGTGTTTTTACACGGGTTTTACATCTACTTTTTTATTTTAAGAAAAAAAAAAAATTAAAGGCAGAAATGTTTGATATATTCATTTATTTCTATATTTAAGACCATTATTTTTATTGAAAAGGCGACAATACTCTTCTAGGGGTTTTTTGAATTGACGTTACATTATTGATTTA

>XM_022146069.2 (HaPYL2c)

CAAGAACTTCATTACAACCTTTTTTTTCTGCCGACACTTCCATTTCCACGTGATCTTCTTCCAACTTCGATTCGTCCTTTTCTCTTCATATCAACCAATGCCCCACACATTGATTCATTCATTCATTTTCCTCACAAAATGGACCAAAACTCAATCCCAGAAGGCCTCACACCAGAAGAATACTCACAACTCCAACTCTTAATCAATGCACACCACATGTTCGACAAAATGCCCAACACATGCACGTCCCTCATAACCCAACACATCGACGCACCCGCACGCGTCGTTTGGCCACTCGTACGCAGCTTCGACAACCCACAACGCTACAAGCATTTCATCAAGAGTTGCAACATGTCTGGGGACGGTGGAGTTGGCAGCATACGCGAAGTGACAGTCATGTCGGGTCTCCCAGCCTCTACTAGCACTGAGCGGCTTGAGGTTCTGGACGACGAGAAGCATATATTGAGTTTTAGAGTTCTGGGTGGGGAGCATAGGTTGAGTAACTATCTTTCTGTTACGTCGGTTAATGAGTTTAAGAAAGGCGAAAAGGTGTACACCGTTGTTTTGGAATCGTATATTGTTGATGTGCCTGTTGGGAACACGGTTGAGGATACGAAGATGTTTACGGATACTGTTGTTAAAATGAATCTACAGAAGTTGGGTCTTGTCGCGTTGTCTTGTTTGCGCGGAAATGAATGATACTGGTTGTCGTGGTGGTGAACTCGTGGTGGCAGTGGATGGGTAACATTAACATCAAATGTATAAGAAAATAAACTAAGATTTTGATGTGGGTGAATGTTTAATTAGTAGGGTTTTTTTTGTTGAAGATGTGTGACTTAAAATAAATTGTTGCATATCTATGGGATTAATGTAATGAATCTGGTAGTTGTAACCAATGTATTTGGAAAATGATATGTTATAATGTTTGGATTTTCAGA

>XM_022150663.2 (HaPYL4g)

TCCTGTATCTATATCTATATATCTATATCTATATCTATATATAATCTTCACATCACAATCTACACAAACCATCATCTCAAAAAATACAAGATGCCTTCTTCCGTTCAAGTTCACAGAATCCACCCAACAACCACCACCACAACCACCACCACCACCGTCAACAACCACAAACAACCATCAACAACAACCATATGGAGGGTCCCATCATCTATTAACATCCCAGAAGATCTTAACCACCACCACACCCATGCAGTTGGGCCCCACCAGTGCTCCTCCGCCGTCGTACAAACCATCGCCGCCCCCATCGCCGCCGTGTGGTCCGTTGTCAGACGGTTCGACAAGCCGCAGGTTTACAAACACTTTCTTAAAAGCTGTCACGTGATTCTTGGTGACGGTGATGTGGGTACTCTGAGAGAAGTCCACGTGGTGTCAGGACTGCCAGCTGGATCCAGCACAGAGCGGTTAGAGATTCTGGATGATGATCGACATGTCATGAGTTTTAGTGTTGTCGGTGGTGACCATCGGCTTAATAATTACCGGTCTGTTACGACTCTTCACGCGTCACCGACATGTGACGGTAGCACTGTTGTTGTGGAGTCTTATGTTGTTGATGTGCCGCCGGAGAATACTAAGGAGGAAACGTGTGTGTTCGTGGATACTATTGTACGATGTAACCTGATTTCGTTGAAGCAAATCGCTGAAAGTAAATAATTTCGAGTTGAAGTGATCGAGTTTGTGAATTAGTTAATCTGTTGTGGATGTTTTTGTTTCCTTTTTTTCATGGGTTTGATTTAATCTGTGATGTGGGTAGAGATTAGAGAGCAATTTCTCAGGGGTTTTTTTTTTTTATTTTTTGTTTTTTGTTTTTTTGTAGATGTGATTTTGAGTAGTGTTTTTTCATGGATTCATGTTACACTTTAGAAGAAAGGATTTAGAGATCTGCATGGAATTTTTGAGCAAATTTTCATTATGTTA

>XM_022154567.2 (HaPYL4e)

TAACACCTTCATGTTCCAACATTAAGAAACAACAATCAAGATTCTTCAACTCATCAACTTTCATCTATATTCATCTTTTAAATCCAACAGATCAAAAAATGCTTTCTAACCCACAAAAGTCACCATCTTTCTTCCTAGACGGAATCAACACAACCACCACCACCACCACTACCGGAAGCTCCGGTAGTTGTGGCACTAACAAGCCACCACCAACCAACACCACTCCGGTACCGGACTCCGTTGCCCGCTACCACATCCACGCCGTGGGTCCAGACCAGTGCTGCTCCGTCGTCATCCAACACATCACCGCCCCAATCTCCGACGTCTGGTCCATCGTCCGCCGCTTCGACAACCCACAAGCCTACAAACACTTTGTCAAAACCTGCCACGTAATCCTCGGAGACGGCAACGTCGGAACCCTAAGAGAAATCCACGTCATCTCCGGCCTGCCCGCTGCCCGTTCCACTGAACGTTTGGAAATCTTAGACGATGAACAACACGTGATCAGTTTCAGCGTCGTTGACGGTGATCACCGGCTTGCTAACTACCGTTCCGTCACCACTCTCCACCCTACACCCGACGGAACCGGTACGGTGGTTGTTGAGTCCTACGTTGTTGACATACCGCCGGGGAATACTAAGGAAGAAACATGCGTGTTTGTTGACACCATCGTGAAATGCAACCTCCAGTCGTTAGCTCAGAAAATCCGGCGAAACTGATTGCCGGAAAATCAAGATTTATTATGATATTTGAGAATACACATCTCGGGAACAAGTACATAAACATAAAGCCATTTTCGAATCGAAGTAGATGATATAACTGATACTTATGAAGATTCAATCGATCATTTAAAGACCTTTATGTTATTTTGGTGGTGTGAATTTTCATCATCAAAGTTTTTGTGAACCCATGTGATCACTTTTTTGAAGGTTTTATTTATTGATTGATTTGGATTTATGAGAAAATGTCCTCTACTTCTTGCA

>XM_022169452.2 (HaPYL4b)

ATCAATTATCATATCACATGCTAAGTACGATCCTGTCAATTCATTCAATAACAAAATCAACCAATCATGTGTTCCTCCATTCAAATCCAAAGAATACAAGAAAACCCTAAATCCACCGTGAACCACCACACCACCACCACCGTACGGAGGGTTCCACCGTCAGTTTACCTACCAGAACACATCCACCACCACCACATCCATACAATGACTCTCAACCAGTGTTCGTCGACAATAGTCCAGACGATCTCTGCTCCAGTAGAGACAGTCTGGTCTGTGGTTCGTCGCTTTGACGAACCACATGCATACAAGCACTTTCTCAAGAGCTGTGACGTCATCGTCGGTGACGGTGACGTGGGTTCTGTGAGGGAGGTTCGGGTGGTATCCGGGTTACCCGCGGGTTCCAGCTTGGAGAGGCTTGAGATCTGTGATGACGAACGGCATGTTATGAGCTTTAGCGTTGTTGGTGGGGACCACCGGCTGAAGAACTACCGGTCGGTTACGACGCTTCACTCTTCGCCGACATGCCGCGTCACCACCGTAGTTGTGGAGTCTTATGTTGTGGACGTGCCACCGGAGAATACTAAGGAGGAAACGTGTGTGTTCGTGGAGACAATCGTACGATGTAACTTGATGTCCCTTAAACATATTGCTCAAAGTTTAGCCCAGAAATAAAACCAGTTTTAGTTTTTGTTGGTTAAATCTTTAAGTAGAGTTATTCTTATAATTTCTTTTTCATGGGATTTTTATCACGTTGTTATTTCATGGTGTAATATTGTAAAATATTTTAGATTACTCTCATTAAAATCCGGTTTTTTATA

>XM_022170317.2 (HaPYL8c)

GTGAGAGAAACCAGATGTGGATGAGAAAGAACCAACGAAAACCCCAATTGCTGCTGCTGCTGATGTGATTATTCTCTCTCGAATTCCTCAACGAATCCACCGGACTTTCAACACTAAATCTATCGCACCGATCAAATTCAGTCACAGATTGTTCGACGATCGTTACTCTTATCCATAACTACCGATGGTGATGAACGGACACGATGACGCTACAGATTCGTCGGAGTACAGCCACATTAGGAGACACCATAAGCATGAAGTTAGAGCCAATCAGTGCACTTCATCTCTCGTTAAACATATCAAAGCGCCTGTTCATCTTGTATGGTCTTTGGTGAGGAGGTTTGATCAACCACAAAAGTACAAACCCTTTGTGAGCGGGTGTTACGTGCAAGGTAACCTTGGAATCGGGAGTGTGAGAGAAGTATGTGTTCGATCTGGGCTTCCAGCTACCACGAGTATGGAGAGGTTAGAGCTTCTCGACGAAGAGGAACATATCTTGGGCATAAAAATAGTCGGCGGTGACCACAGGCTTCGGAACTACTCGTCAATTATGACACTTCATCCAGAGATTATCGACGGGAGGCCAGGGACTTTGGTGATTGAATCGTTCGTGGTGGACGTGCCAGATGGCAACACGAAAGATGAGACGTGCTACTTTGTGGAGGCTCTCATTAAATGCAACCTTAAGTCGTTAGCTGATGTATCGGAGCGTTTGGCTGTGCAGGACAGAACAGAACCCATAGTTGGCGTGTGATTCTAGTGGAAGTACAAGTTAGTTGTAGACTGGTCAAAGTCCGTGTTTGTGCATATAGCATTAGAGACTTTCTTGCTAAATTGATGGGGTGTGTTTCGTAAATCATGTTTCTGTTTTCGAACATCATGTTTGACACTCATTGTCGTGTTCTTTGTCTTTGTACTTGTTGTTAAGCCGTTTTTTTGTGTGAATACAAATAAATAGAATAGGAAGTTTTGCA

>XM_022173057.2 (HaPYL2a)

CCACGTGATTTCTTTCAAACATAACTCATATCTTCTCTCTTTCATAATCAACTAACGACCTCCACTAATATGCAACACTTTCAGTCATCATCTACCCACTTGAAATGGAACCAAAATCAATCCCACAAGGCCTCACAGCAGACGAATACTCCGAGCTTCAACTCTTAATCAATGCATACCACCTGTTCGACAAAATTCCAAACACATGCACATCCCTAATAACCCAACGCATAGACGCGCCCACAAACATAGTGTGGCCTCTAGTACGCCGTTTCGACAACCCGCAACGATACAAGCATTTTATCAAGAGTTGCAGCATGTTTGGGGATGGTGGTGTGGGAAGCATACGTGAGGTTACGGTCATATCGGGCCTCCCGGCCTCCACGAGTACTGAACGGCTGGAGTTGTTGGATGATGAGAAGCATATTTTGAGCTTTAGAGTGGTGGGTGGTGAGCATAGATTGAATAACTATCTTTCGGTTACGTCGGTTAATGAGTTTGAGAAAGATGGGAAGGTTTATACGGTGGTGTTGGAGTCTTATATTGTTGATATACCTACGGGGAATACGGTGGAGGATACTAAGATGTTTACGGATACGGTTGTGAAGTTGAATTTGCAAAAGTTGGGTCTTGTGGCAATGGCTTGTTTGCATGGGAGTGAATGAGTAGGTTTGGTACGGTGGTTGTGGTGGTGGTGACAGTGGATGTACGGTGGTTAGCAGTGGGGACTTGGGTTGGTAGCATTAACATGTGAAAGTTAAATAAATCAAGATTTTGATGGGGCCGTGTGATCTTTTGTATTGTTAATTTTTCTTTTTATAGTTGACGATGTGTAATTTCTAATAGAGTTAAATAAACTCCATGTGGTTTGGAACATTTTACTAGTTTAGTTTAAAGATTTTATTTTTTGCATGTAGGTCCAAAAAGGTTTTACCGTTGCCATTTTAGTCCACTAAGTTAACTTCATTCATTTTTTTCTGTTAACAAGAAGGGCAATGCAGTCATTTTATATGTAATTTTGTTAACTATAAGGGGAATTCGGCCATATAAAATGACCAAATTGATGTTCTCGTTAACAGAAAAAATGGATGAAATTAACACAGTGGACTAAAATGAAAACGGTGAAACTTTTTTGAACCCACAGACTAAAAATGAAACCTTTGGATTAAACTAGCAAAATGACCAAACCACAGGGAAATTTAACTCTTTCTAATAAGTCATTTGCATTTGGGGTCGA

>XM_022180006.2 (HaPYL9a)

ACCATTTGGATGGAGCCACTTTATAATTCTTTCTTTGTCGGTAGTGGATTTAGTGATTCCAATAATTCATAACCACACCACACACACACTATTAGTATTCACTATTTTCATCACAAATTCCCCATCTTCACCTTTAAACAACCAAAAAAAAATTCTAGTTGTTAATCTTCGTAACTTTTTGAACCATACAGCCAGATGTTTCCTTCATATTACACGATTTAAATCGAGATCGAAGAAACTGATGAAGAATGGATGCAGCTCACTATATAAGAAGACACCACAGGCATACTCTTGGACATAAACAGTGCGCTTCTGTCGTCGTTAGACACATCAAAGCTCCCGTTGATATCGTGTGGTCATTAGTAAGGAGATTTGATGAACCTCAGAAGTACAAGCCCTTTGTTAGTAGGTGCACTATGCATGGGGAGATTAATATTGGAAGTGTTAGAGAGGTGAATGTCAAGTCAGGGCTTCCAGCCACTACCAGCACAGAGAGGTTGGAACTTCTTGATGACAAGGAGCACATACTTGGCATCAAAATTGTTGGTGGTGACCATAGGCTAAAGAACTATTCGTCAATCCTAACCGTTCATCCAGAGGTAAGCGAAGGATCTGGGACGTTAGTTGTGGAGTCATTTGTGGTGGACATACCCGATGGCAACACTATGGAAGAAACATGCTACTTTGTTAGAGCCTTAATCAATTGCAACCTTAAATGTCTTTCAGATGTCTCGGAAAGAATGGCCATTTGTCCGGATTCAGGCATGAAAAGATCAGAAATGGCTCATGGTGTTCCGGTTTAGCGTCTTCTATTTTGTTAATGTTCAAGTTATTGTAGAACATATAGTTGGTTATGAGATTTACTGGGTATGGTTGTTGTTGTTATTGTTGTTGTTTTAGTGGCAGCTCAGTGTTTGCAAGACTGCAAACATTTTGTAGCGGTTTATATTTCCTAAGACATGATTCCAAACAAATGGCATTTGGGTTA

**Additional File 5. List of primers used for qRT-PCR validation**

| Sequence accession | Primer name | Sequence of primer |
| --- | --- | --- |
| XM_022113537.2 | HaPYL8a-F | TTGTAGATGTGCCGGATGGT |
|  | HaPYL8a-R | GACTCTGTTCGGTCTTGCAC |
| XM_022113657.2 | HaPYR1a-F | CACCACAAACCACCACAACT |
|  | HaPYR1a-R | TGGGAGGGTGAGAGATGGTA |
| XM_022113987.2 | HaPYL9b-F | ATACGAAGACACCACAGGCA |
|  | HaPYL9b-R | ATCCACCTCTCGAACACTCC |
| XM_022116937.2 | HaPYL2b-F | CAATCCCACAAGGCCTAACC |
|  | HaPYL2b-R | ACCTTTGTGGGTTGTCGAAC |
| XM_022118303.2 | HaPYL4c-F | CCGTCAACCACCACAAACAA |
|  | HaPYL4c-R | GGACCAACGACATGAGTGTG |
| XM_022118530.2 | HaPYL8b-F | CCGAGAGGTTGGAACTCCTT |
|  | HaPYL8b-R | CCTTCTGGCACATCAACCAC |
| XM_022120093.2 | HaPYL2d-F | GGGCAGTATCCGGGAAGTTA |
|  | HaPYL2d-R | GTGTTCACCACCCACAACTC |
| XM_022121546.2 | HaPYL4d-F | ACCACCAGTCTAGCACCATC |
|  | HaPYL4d-R | AAGCATTGGTTGTGGGACAC |
| XM_022126755.2 | HaPYL9c-F | GGTGGTGATCACAGACTCCA |
|  | HaPYL9c-R | AAACGCTCTGACACGTTAGC |
| XM_022127849.2 | HaPYL4a-F | ACGTCAAGTCCACGTCATCT |
|  | HaPYL4a-R | GACGGAGCGGTAGTTCGATA |
| XM_022132184.2 | HaPYL4f-F | CCCACCACCACTATTCCGAT |
|  | HaPYL4f-R | GGCGTTGATGTGTTGGATCA |
| XM_022141537.2 | HaPYR1b-F | TGCACGCGTGATGTTATTGT |
|  | HaPYR1b-R | ATTTGGTGCACTGTGGTGAC |
| XM_022146069.2 | HaPYL2c-F | CTCCCAGCCTCTACTAGCAC |
|  | HaPYL2c-R | CCCAACAGGCACATCAACAA |
| XM_022150663.2 | HaPYL4g-F | TTAACCACCACCACACCCAT |
|  | HaPYL4g-R | ACCACGTGGACTTCTCTCAG |
| XM_022154567.2 | HaPYL4e-F | CACGTGATCAGTTTCAGCGT |
|  | HaPYL4e-R | TGGTGTCAACAAACACGCAT |
| XM_022169452.2 | HaPYL4b-F | CAGACGATCTCTGCTCCAGT |
|  | HaPYL4b-R | CGATGACGTCACAGCTCTTG |
| XM_022170317.2 | HaPYL8c-F | TGCTACTTTGTGGAGGCTCT |
|  | HaPYL8c-R | CACGCCAACTATGGGTTCTG |
| XM_022173057.2 | HaPYL2a-F | ATCAATCCCACAAGGCCTCA |
|  | HaPYL2a-R | TGTCGAAACGGCGTACTAGA |
| XM_022180006.2 | HaPYL9a-F | TAAACAGTGCGCTTCTGTCG |
|  | HaPYL9a-R | TGGAAGCCCTGACTTGACAT |

**Additional File 6. Gene Ontology enrichment of *HaPYL*s**

| name | GO | KO_id | KO_name | paths | cog | cog_description | pfam | swissprot | entrez |
| --- | --- | --- | --- | --- | --- | --- | --- | --- | --- |
| HaPYL2a |  | K14496 | PYL | map04016(MAPK signaling pathway - plant); map04075(Plant hormone signal transduction) | ENOG410YE0A(S:Function unknown) | ENOG410YE0A(abscisic acid receptor) | PF10604.6(Polyketide_cyc2:Polyketide cyclase / dehydrase and lipid transport); PF00407.16(Bet_v_1:Pathogenesis-related protein Bet v I family) | sp\|O80992\|PYL2_ARATH(Abscisic acid receptor PYL2 OS=Arabidopsis thaliana OX=3702 GN=PYL2 PE=1 SV=1) | 110929874 |
| HaPYL9a |  | K14496 | PYL | map04016(MAPK signaling pathway - plant); map04075(Plant hormone signal transduction) | ENOG4111HU5(S:Function unknown); ENOG41114FA(S:Function unknown) | ENOG4111HU5(abscisic acid receptor); ENOG41114FA(Polyketide cyclase / dehydrase and lipid transport) | PF10604.6(Polyketide_cyc2:Polyketide cyclase / dehydrase and lipid transport) | sp\|Q84MC7\|PYL9_ARATH(Abscisic acid receptor PYL9 OS=Arabidopsis thaliana OX=3702 GN=PYL9 PE=1 SV=1) | 110937569 |
| HaPYL8a | GO:0005737(cellular_component:cytoplasm); GO:0005634(cellular_component:nucleus); GO:0009738(biological_process:abscisic acid-activated signaling pathway); GO:0080163(biological_process:regulation of protein serine/threonine phosphatase activity); GO:0004864(molecular_function:protein phosphatase inhibitor activity); GO:0004872(molecular_function:receptor activity); GO:0043086(biological_process:negative regulation of catalytic activity); GO:0010427(molecular_function:abscisic acid binding) | K14496 | PYL | map04016(MAPK signaling pathway - plant); map04075(Plant hormone signal transduction) | ENOG4111HU5(S:Function unknown) | ENOG4111HU5(abscisic acid receptor) | PF10604.6(Polyketide_cyc2:Polyketide cyclase / dehydrase and lipid transport) | sp\|Q9FGM1\|PYL8_ARATH(Abscisic acid receptor PYL8 OS=Arabidopsis thaliana OX=3702 GN=PYL8 PE=1 SV=1) | 110864467 |
| HaPYR1a |  | K14496 | PYL | map04016(MAPK signaling pathway - plant); map04075(Plant hormone signal transduction) | ENOG410YDHK(S:Function unknown) | ENOG410YDHK(abscisic acid receptor) | PF10604.6(Polyketide_cyc2:Polyketide cyclase / dehydrase and lipid transport); PF03364.17(Polyketide_cyc:Polyketide cyclase / dehydrase and lipid transport) | sp\|Q8VZS8\|PYL1_ARATH(Abscisic acid receptor PYL1 OS=Arabidopsis thaliana OX=3702 GN=PYL1 PE=1 SV=1) | 110864553 |
| HaPYL9b |  | K14496 | PYL | map04016(MAPK signaling pathway - plant); map04075(Plant hormone signal transduction) | ENOG4111HU5(S:Function unknown); ENOG41114FA(S:Function unknown) | ENOG4111HU5(abscisic acid receptor); ENOG41114FA(Polyketide cyclase / dehydrase and lipid transport) | PF10604.6(Polyketide_cyc2:Polyketide cyclase / dehydrase and lipid transport) | sp\|Q84MC7\|PYL9_ARATH(Abscisic acid receptor PYL9 OS=Arabidopsis thaliana OX=3702 GN=PYL9 PE=1 SV=1) | 110864828 |
| HaPYL4c | GO:0005737(cellular_component:cytoplasm); GO:0016021(cellular_component:integral component of membrane); GO:0009738(biological_process:abscisic acid-activated signaling pathway); GO:0080163(biological_process:regulation of protein serine/threonine phosphatase activity); GO:0004864(molecular_function:protein phosphatase inhibitor activity); GO:0004872(molecular_function:receptor activity); GO:0005634(cellular_component:nucleus); GO:0043086(biological_process:negative regulation of catalytic activity); GO:0010427(molecular_function:abscisic acid binding) | K14496 | PYL | map04016(MAPK signaling pathway - plant); map04075(Plant hormone signal transduction) | ENOG4111MYF(S:Function unknown); ENOG4111D40(S:Function unknown) | ENOG4111MYF(abscisic acid receptor); ENOG4111D40(abscisic acid receptor) | PF10604.6(Polyketide_cyc2:Polyketide cyclase / dehydrase and lipid transport); PF00407.16(Bet_v_1:Pathogenesis-related protein Bet v I family); PF03364.17(Polyketide_cyc:Polyketide cyclase / dehydrase and lipid transport) | sp\|O80920\|PYL4_ARATH(Abscisic acid receptor PYL4 OS=Arabidopsis thaliana OX=3702 GN=PYL4 PE=1 SV=1) | 110869013 |
| HaPYL8b | GO:0005737(cellular_component:cytoplasm); GO:0005634(cellular_component:nucleus); GO:0009738(biological_process:abscisic acid-activated signaling pathway); GO:0080163(biological_process:regulation of protein serine/threonine phosphatase activity); GO:0004864(molecular_function:protein phosphatase inhibitor activity); GO:0004872(molecular_function:receptor activity); GO:0043086(biological_process:negative regulation of catalytic activity); GO:0010427(molecular_function:abscisic acid binding) | K14496 | PYL | map04016(MAPK signaling pathway - plant); map04075(Plant hormone signal transduction) | ENOG4111HU5(S:Function unknown) | ENOG4111HU5(abscisic acid receptor) | PF10604.6(Polyketide_cyc2:Polyketide cyclase / dehydrase and lipid transport) | sp\|Q9FGM1\|PYL8_ARATH(Abscisic acid receptor PYL8 OS=Arabidopsis thaliana OX=3702 GN=PYL8 PE=1 SV=1) | 110869242 |
| HaPYL4d | GO:0005737(cellular_component:cytoplasm); GO:0005634(cellular_component:nucleus); GO:0009738(biological_process:abscisic acid-activated signaling pathway); GO:0080163(biological_process:regulation of protein serine/threonine phosphatase activity); GO:0004864(molecular_function:protein phosphatase inhibitor activity); GO:0004872(molecular_function:receptor activity); GO:0010427(molecular_function:abscisic acid binding) | K14496 | PYL | map04016(MAPK signaling pathway - plant); map04075(Plant hormone signal transduction) | ENOG4111MYF(S:Function unknown); ENOG4111D40(S:Function unknown) | ENOG4111MYF(abscisic acid receptor); ENOG4111D40(abscisic acid receptor) | PF10604.6(Polyketide_cyc2:Polyketide cyclase / dehydrase and lipid transport); PF00407.16(Bet_v_1:Pathogenesis-related protein Bet v I family) | sp\|O80920\|PYL4_ARATH(Abscisic acid receptor PYL4 OS=Arabidopsis thaliana OX=3702 GN=PYL4 PE=1 SV=1) | 110872687 |
| HaPYL2d |  | K14496 | PYL | map04016(MAPK signaling pathway - plant); map04075(Plant hormone signal transduction) | ENOG410YE0A(S:Function unknown) | ENOG410YE0A(abscisic acid receptor) | PF10604.6(Polyketide_cyc2:Polyketide cyclase / dehydrase and lipid transport); PF00407.16(Bet_v_1:Pathogenesis-related protein Bet v I family); PF03364.17(Polyketide_cyc:Polyketide cyclase / dehydrase and lipid transport) | sp\|O80992\|PYL2_ARATH(Abscisic acid receptor PYL2 OS=Arabidopsis thaliana OX=3702 GN=PYL2 PE=1 SV=1) | 110871181 |
| HaPYL9c | GO:0005737(cellular_component:cytoplasm); GO:0005634(cellular_component:nucleus); GO:0009738(biological_process:abscisic acid-activated signaling pathway); GO:0080163(biological_process:regulation of protein serine/threonine phosphatase activity); GO:0004864(molecular_function:protein phosphatase inhibitor activity); GO:0004872(molecular_function:receptor activity); GO:0043086(biological_process:negative regulation of catalytic activity); GO:0010427(molecular_function:abscisic acid binding) | K14496 | PYL | map04016(MAPK signaling pathway - plant); map04075(Plant hormone signal transduction) | ENOG4111HU5(S:Function unknown) | ENOG4111HU5(abscisic acid receptor) | PF10604.6(Polyketide_cyc2:Polyketide cyclase / dehydrase and lipid transport) | sp\|Q9FGM1\|PYL8_ARATH(Abscisic acid receptor PYL8 OS=Arabidopsis thaliana OX=3702 GN=PYL8 PE=1 SV=1) | 110878452 |
| HaPYL4a | GO:0005737(cellular_component:cytoplasm); GO:0005634(cellular_component:nucleus); GO:0009738(biological_process:abscisic acid-activated signaling pathway); GO:0080163(biological_process:regulation of protein serine/threonine phosphatase activity); GO:0004864(molecular_function:protein phosphatase inhibitor activity); GO:0004872(molecular_function:receptor activity); GO:0010427(molecular_function:abscisic acid binding) | K14496 | PYL | map04016(MAPK signaling pathway - plant); map04075(Plant hormone signal transduction) | ENOG4111MYF(S:Function unknown); ENOG4111D40(S:Function unknown) | ENOG4111MYF(abscisic acid receptor); ENOG4111D40(abscisic acid receptor) | PF10604.6(Polyketide_cyc2:Polyketide cyclase / dehydrase and lipid transport); PF03364.17(Polyketide_cyc:Polyketide cyclase / dehydrase and lipid transport); PF00407.16(Bet_v_1:Pathogenesis-related protein Bet v I family) | sp\|O80920\|PYL4_ARATH(Abscisic acid receptor PYL4 OS=Arabidopsis thaliana OX=3702 GN=PYL4 PE=1 SV=1) | 110879398 |
| HaPYL4f |  | K14496 | PYL | map04016(MAPK signaling pathway - plant); map04075(Plant hormone signal transduction) | ENOG4111MYF(S:Function unknown); ENOG4111D40(S:Function unknown) | ENOG4111MYF(abscisic acid receptor); ENOG4111D40(abscisic acid receptor) | PF10604.6(Polyketide_cyc2:Polyketide cyclase / dehydrase and lipid transport); PF00407.16(Bet_v_1:Pathogenesis-related protein Bet v I family); PF03364.17(Polyketide_cyc:Polyketide cyclase / dehydrase and lipid transport) | sp\|O80920\|PYL4_ARATH(Abscisic acid receptor PYL4 OS=Arabidopsis thaliana OX=3702 GN=PYL4 PE=1 SV=1) | 110884474 |
| HaPYR1b |  | K14496 | PYL | map04016(MAPK signaling pathway - plant); map04075(Plant hormone signal transduction) | ENOG410YDHK(S:Function unknown) | ENOG410YDHK(abscisic acid receptor) | PF10604.6(Polyketide_cyc2:Polyketide cyclase / dehydrase and lipid transport); PF03364.17(Polyketide_cyc:Polyketide cyclase / dehydrase and lipid transport) | sp\|Q8VZS8\|PYL1_ARATH(Abscisic acid receptor PYL1 OS=Arabidopsis thaliana OX=3702 GN=PYL1 PE=1 SV=1) | 110894325 |
| HaPYL2c |  | K14496 | PYL | map04016(MAPK signaling pathway - plant); map04075(Plant hormone signal transduction) | ENOG410YE0A(S:Function unknown) | ENOG410YE0A(abscisic acid receptor) | PF10604.6(Polyketide_cyc2:Polyketide cyclase / dehydrase and lipid transport); PF00407.16(Bet_v_1:Pathogenesis-related protein Bet v I family); PF03364.17(Polyketide_cyc:Polyketide cyclase / dehydrase and lipid transport) | sp\|O80992\|PYL2_ARATH(Abscisic acid receptor PYL2 OS=Arabidopsis thaliana OX=3702 GN=PYL2 PE=1 SV=1) | 110899197 |
| HaPYL4g | GO:0005737(cellular_component:cytoplasm); GO:0016020(cellular_component:membrane); GO:0005634(cellular_component:nucleus); GO:0009738(biological_process:abscisic acid-activated signaling pathway); GO:0080163(biological_process:regulation of protein serine/threonine phosphatase activity); GO:0004864(molecular_function:protein phosphatase inhibitor activity); GO:0004872(molecular_function:receptor activity); GO:0010427(molecular_function:abscisic acid binding) | K14496 | PYL | map04016(MAPK signaling pathway - plant); map04075(Plant hormone signal transduction) | ENOG4111MYF(S:Function unknown); ENOG4111D40(S:Function unknown) | ENOG4111MYF(abscisic acid receptor); ENOG4111D40(abscisic acid receptor) | PF10604.6(Polyketide_cyc2:Polyketide cyclase / dehydrase and lipid transport); PF00407.16(Bet_v_1:Pathogenesis-related protein Bet v I family) | sp\|O80920\|PYL4_ARATH(Abscisic acid receptor PYL4 OS=Arabidopsis thaliana OX=3702 GN=PYL4 PE=1 SV=1) | 110904802 |
| HaPYL4e | GO:0005737(cellular_component:cytoplasm); GO:0005634(cellular_component:nucleus); GO:0009738(biological_process:abscisic acid-activated signaling pathway); GO:0080163(biological_process:regulation of protein serine/threonine phosphatase activity); GO:0004864(molecular_function:protein phosphatase inhibitor activity); GO:0004872(molecular_function:receptor activity); GO:0043086(biological_process:negative regulation of catalytic activity); GO:0010427(molecular_function:abscisic acid binding) | K14496 | PYL | map04016(MAPK signaling pathway - plant); map04075(Plant hormone signal transduction) | ENOG4111MYF(S:Function unknown); ENOG4111D40(S:Function unknown) | ENOG4111MYF(abscisic acid receptor); ENOG4111D40(abscisic acid receptor) | PF10604.6(Polyketide_cyc2:Polyketide cyclase / dehydrase and lipid transport); PF00407.16(Bet_v_1:Pathogenesis-related protein Bet v I family); PF03364.17(Polyketide_cyc:Polyketide cyclase / dehydrase and lipid transport) | sp\|O80920\|PYL4_ARATH(Abscisic acid receptor PYL4 OS=Arabidopsis thaliana OX=3702 GN=PYL4 PE=1 SV=1) | 110909816 |
| HaPYL8c | GO:0005737(cellular_component:cytoplasm); GO:0005634(cellular_component:nucleus); GO:0009738(biological_process:abscisic acid-activated signaling pathway); GO:0080163(biological_process:regulation of protein serine/threonine phosphatase activity); GO:0004864(molecular_function:protein phosphatase inhibitor activity); GO:0004872(molecular_function:receptor activity); GO:0043086(biological_process:negative regulation of catalytic activity); GO:0010427(molecular_function:abscisic acid binding) | K14496 | PYL | map04016(MAPK signaling pathway - plant); map04075(Plant hormone signal transduction) | ENOG4111HU5(S:Function unknown) | ENOG4111HU5(abscisic acid receptor) | PF10604.6(Polyketide_cyc2:Polyketide cyclase / dehydrase and lipid transport) | sp\|Q9FGM1\|PYL8_ARATH(Abscisic acid receptor PYL8 OS=Arabidopsis thaliana OX=3702 GN=PYL8 PE=1 SV=1) | 110926532 |
| HaPYL4b |  | K14496 | PYL | map04016(MAPK signaling pathway - plant); map04075(Plant hormone signal transduction) | ENOG4111MYF(S:Function unknown); ENOG4111D40(S:Function unknown) | ENOG4111MYF(abscisic acid receptor); ENOG4111D40(abscisic acid receptor) | PF10604.6(Polyketide_cyc2:Polyketide cyclase / dehydrase and lipid transport); PF00407.16(Bet_v_1:Pathogenesis-related protein Bet v I family); PF03364.17(Polyketide_cyc:Polyketide cyclase / dehydrase and lipid transport) | sp\|O80920\|PYL4_ARATH(Abscisic acid receptor PYL4 OS=Arabidopsis thaliana OX=3702 GN=PYL4 PE=1 SV=1) | 110925507 |
| HaPYL2b |  | K14496 | PYL | map04016(MAPK signaling pathway - plant); map04075(Plant hormone signal transduction) | ENOG410YE0A(S:Function unknown) | ENOG410YE0A(abscisic acid receptor) | PF10604.6(Polyketide_cyc2:Polyketide cyclase / dehydrase and lipid transport); PF03364.17(Polyketide_cyc:Polyketide cyclase / dehydrase and lipid transport); PF00407.16(Bet_v_1:Pathogenesis-related protein Bet v I family) | sp\|O80992\|PYL2_ARATH(Abscisic acid receptor PYL2 OS=Arabidopsis thaliana OX=3702 GN=PYL2 PE=1 SV=1) | 110867882 |

**Additional File 7. Protein interaction network of the HaPYL protein with other sunflower proteins predicted by the STRING online service**

| node1 | node2 | node1_string_id | node2_string_id | coexpression | experimentally_determined_interaction | database_annotated | automated_textmining | combined_score |
| --- | --- | --- | --- | --- | --- | --- | --- | --- |
| A0A251S3H5 | ATPP2CA | 4232.A0A251S3H5 | 4232.A0A251TTP8 | 0 | 0.144 | 0.665 | 0.176 | 0.743 |
| A0A251S3H5 | A0A251UCX7 | 4232.A0A251S3H5 | 4232.A0A251UCX7 | 0 | 0.144 | 0.665 | 0.176 | 0.743 |
| A0A251S3H5 | A0A251UJ72 | 4232.A0A251S3H5 | 4232.A0A251UJ72 | 0 | 0.144 | 0.665 | 0.176 | 0.743 |
| A0A251S3H5 | A0A251UN79 | 4232.A0A251S3H5 | 4232.A0A251UN79 | 0 | 0.144 | 0.665 | 0.176 | 0.743 |
| A0A251S3H5 | A0A251U9L3 | 4232.A0A251S3H5 | 4232.A0A251U9L3 | 0 | 0.144 | 0.665 | 0.176 | 0.743 |
| A0A251S3H5 | A0A251UCZ1 | 4232.A0A251S3H5 | 4232.A0A251UCZ1 | 0 | 0.144 | 0.665 | 0.176 | 0.743 |
| A0A251S3H5 | A0A251TP95 | 4232.A0A251S3H5 | 4232.A0A251TP95 | 0 | 0.144 | 0.665 | 0.176 | 0.743 |
| A0A251S3H5 | A0A251UEA5 | 4232.A0A251S3H5 | 4232.A0A251UEA5 | 0 | 0.144 | 0.665 | 0.176 | 0.743 |
| A0A251S3H5 | HAI2 | 4232.A0A251S3H5 | 4232.A0A251SSF2 | 0 | 0.144 | 0.665 | 0.202 | 0.751 |
| A0A251S3H5 | A0A251UNA0 | 4232.A0A251S3H5 | 4232.A0A251UNA0 | 0 | 0.144 | 0.665 | 0.202 | 0.751 |
| A0A251S3H5 | A0A251SAZ2 | 4232.A0A251S3H5 | 4232.A0A251SAZ2 | 0 | 0.144 | 0.665 | 0.202 | 0.751 |
| A0A251S3H5 | ABI2 | 4232.A0A251S3H5 | 4232.A0A251TQT8 | 0 | 0.144 | 0.665 | 0.222 | 0.757 |
| A0A251S3H5 | A0A251U0K7 | 4232.A0A251S3H5 | 4232.A0A251U0K7 | 0 | 0.144 | 0.665 | 0.222 | 0.757 |
| A0A251S3H5 | A0A251SBW4 | 4232.A0A251S3H5 | 4232.A0A251SBW4 | 0 | 0.144 | 0.665 | 0.252 | 0.767 |
| A0A251S3H5 | P2C06 | 4232.A0A251S3H5 | 4232.A0A251TU99 | 0 | 0.273 | 0.665 | 0.208 | 0.79 |
| A0A251S3H5 | HAB1 | 4232.A0A251S3H5 | 4232.A0A251U1I6 | 0 | 0.273 | 0.665 | 0.208 | 0.79 |
| A0A251S3H5 | A0A251TQ41 | 4232.A0A251S3H5 | 4232.A0A251TQ41 | 0 | 0.273 | 0.665 | 0.208 | 0.79 |
| HaPYL4e | A0A251U9L3 | 4232.A0A251S413 | 4232.A0A251U9L3 | 0 | 0.259 | 0.65 | 0.08 | 0.74 |
| HaPYL4e | A0A251UN79 | 4232.A0A251S413 | 4232.A0A251UN79 | 0 | 0.259 | 0.65 | 0.08 | 0.74 |
| HaPYL4e | A0A251UJ72 | 4232.A0A251S413 | 4232.A0A251UJ72 | 0 | 0.259 | 0.65 | 0.08 | 0.74 |
| HaPYL4e | A0A251UCX7 | 4232.A0A251S413 | 4232.A0A251UCX7 | 0 | 0.259 | 0.65 | 0.086 | 0.742 |
| HaPYL4e | ATPP2CA | 4232.A0A251S413 | 4232.A0A251TTP8 | 0 | 0.259 | 0.65 | 0.086 | 0.742 |
| HaPYL4e | A0A251UCZ1 | 4232.A0A251S413 | 4232.A0A251UCZ1 | 0 | 0.259 | 0.65 | 0.086 | 0.742 |
| HaPYL4e | A0A251TP95 | 4232.A0A251S413 | 4232.A0A251TP95 | 0 | 0.259 | 0.65 | 0.086 | 0.742 |
| HaPYL4e | A0A251UEA5 | 4232.A0A251S413 | 4232.A0A251UEA5 | 0 | 0.259 | 0.65 | 0.086 | 0.742 |
| HaPYL4e | HAB1 | 4232.A0A251S413 | 4232.A0A251U1I6 | 0 | 0.273 | 0.65 | 0.181 | 0.773 |
| HaPYL4e | A0A251TQ41 | 4232.A0A251S413 | 4232.A0A251TQ41 | 0 | 0.273 | 0.65 | 0.181 | 0.773 |
| HaPYL4e | P2C06 | 4232.A0A251S413 | 4232.A0A251TU99 | 0 | 0.273 | 0.65 | 0.181 | 0.773 |
| A0A251SAY3 | A0A251UCX7 | 4232.A0A251SAY3 | 4232.A0A251UCX7 | 0 | 0.144 | 0.665 | 0.105 | 0.721 |
| A0A251SAY3 | A0A251UJ72 | 4232.A0A251SAY3 | 4232.A0A251UJ72 | 0 | 0.144 | 0.665 | 0.105 | 0.721 |
| A0A251SAY3 | A0A251UN79 | 4232.A0A251SAY3 | 4232.A0A251UN79 | 0 | 0.144 | 0.665 | 0.105 | 0.721 |
| A0A251SAY3 | A0A251UCZ1 | 4232.A0A251SAY3 | 4232.A0A251UCZ1 | 0 | 0.144 | 0.665 | 0.105 | 0.721 |
| A0A251SAY3 | A0A251U9L3 | 4232.A0A251SAY3 | 4232.A0A251U9L3 | 0 | 0.144 | 0.665 | 0.105 | 0.721 |
| A0A251SAY3 | A0A251UEA5 | 4232.A0A251SAY3 | 4232.A0A251UEA5 | 0 | 0.144 | 0.665 | 0.105 | 0.721 |
| A0A251SAY3 | A0A251TP95 | 4232.A0A251SAY3 | 4232.A0A251TP95 | 0 | 0.144 | 0.665 | 0.105 | 0.721 |
| A0A251SAY3 | ATPP2CA | 4232.A0A251SAY3 | 4232.A0A251TTP8 | 0 | 0.144 | 0.665 | 0.105 | 0.721 |
| A0A251SAY3 | A0A251U0K7 | 4232.A0A251SAY3 | 4232.A0A251U0K7 | 0 | 0.144 | 0.665 | 0.174 | 0.742 |
| A0A251SAY3 | ABI2 | 4232.A0A251SAY3 | 4232.A0A251TQT8 | 0 | 0.144 | 0.665 | 0.174 | 0.742 |
| A0A251SAY3 | A0A251UNA0 | 4232.A0A251SAY3 | 4232.A0A251UNA0 | 0 | 0.144 | 0.665 | 0.176 | 0.743 |
| A0A251SAY3 | A0A251SAZ2 | 4232.A0A251SAY3 | 4232.A0A251SAZ2 | 0 | 0.144 | 0.665 | 0.176 | 0.743 |
| A0A251SAY3 | HAI2 | 4232.A0A251SAY3 | 4232.A0A251SSF2 | 0 | 0.144 | 0.665 | 0.176 | 0.743 |
| A0A251SAY3 | A0A251SBW4 | 4232.A0A251SAY3 | 4232.A0A251SBW4 | 0 | 0.144 | 0.665 | 0.219 | 0.756 |
| A0A251SAY3 | HAB1 | 4232.A0A251SAY3 | 4232.A0A251U1I6 | 0 | 0.386 | 0.665 | 0.158 | 0.812 |
| A0A251SAY3 | A0A251TQ41 | 4232.A0A251SAY3 | 4232.A0A251TQ41 | 0 | 0.386 | 0.665 | 0.158 | 0.812 |
| A0A251SAY3 | P2C06 | 4232.A0A251SAY3 | 4232.A0A251TU99 | 0 | 0.386 | 0.665 | 0.158 | 0.812 |
| A0A251SAZ2 | HaPYL8c | 4232.A0A251SAZ2 | 4232.A0A251RLE6 | 0 | 0.203 | 0.65 | 0.153 | 0.743 |
| A0A251SAZ2 | OST1 | 4232.A0A251SAZ2 | 4232.A0A251RWW9 | 0 | 0.144 | 0.665 | 0.251 | 0.766 |
| A0A251SAZ2 | MPK4 | 4232.A0A251SAZ2 | 4232.A0A251TTG0 | 0.06 | 0.192 | 0.081 | 0.654 | 0.726 |
| A0A251SAZ2 | HaPYR1a | 4232.A0A251SAZ2 | 4232.A0A251UG74 | 0 | 0.105 | 0.65 | 0.222 | 0.735 |
| A0A251SAZ2 | HaPYL8b | 4232.A0A251SAZ2 | 4232.A0A251UE63 | 0 | 0.203 | 0.65 | 0.153 | 0.743 |
| A0A251SAZ2 | HaPYL9b | 4232.A0A251SAZ2 | 4232.A0A251UGY0 | 0 | 0.203 | 0.65 | 0.153 | 0.743 |
| A0A251SAZ2 | HaPYL9a | 4232.A0A251SAZ2 | 4232.A0A251V2E7 | 0 | 0.203 | 0.65 | 0.153 | 0.743 |
| A0A251SAZ2 | HaPYL8a | 4232.A0A251SAZ2 | 4232.A0A251UGS1 | 0 | 0.203 | 0.65 | 0.153 | 0.743 |
| A0A251SAZ2 | HaPYL9c | 4232.A0A251SAZ2 | 4232.A0A251TWF7 | 0 | 0.203 | 0.65 | 0.153 | 0.743 |
| A0A251SAZ2 | A0A251TFU6 | 4232.A0A251SAZ2 | 4232.A0A251TFU6 | 0 | 0.144 | 0.665 | 0.185 | 0.746 |
| A0A251SAZ2 | A0A251SL15 | 4232.A0A251SAZ2 | 4232.A0A251SL15 | 0 | 0.144 | 0.665 | 0.202 | 0.751 |
| A0A251SAZ2 | A0A251UJ18 | 4232.A0A251SAZ2 | 4232.A0A251UJ18 | 0 | 0.144 | 0.665 | 0.202 | 0.751 |
| A0A251SAZ2 | ASK2 | 4232.A0A251SAZ2 | 4232.A0A251TBT3 | 0 | 0.144 | 0.665 | 0.202 | 0.751 |
| A0A251SAZ2 | SNRK2-10 | 4232.A0A251SAZ2 | 4232.A0A251TJT8 | 0 | 0.144 | 0.665 | 0.202 | 0.751 |
| A0A251SAZ2 | HaPYL2a | 4232.A0A251SAZ2 | 4232.A0A251V933 | 0 | 0.27 | 0.65 | 0.108 | 0.752 |
| A0A251SAZ2 | HaPYL2d | 4232.A0A251SAZ2 | 4232.A0A251U5Y4 | 0 | 0.27 | 0.65 | 0.108 | 0.752 |
| A0A251SAZ2 | HaPYL2c | 4232.A0A251SAZ2 | 4232.A0A251STT1 | 0 | 0.27 | 0.65 | 0.108 | 0.752 |
| A0A251SAZ2 | HaPYL2b | 4232.A0A251SAZ2 | 4232.A0A251UAP0 | 0 | 0.27 | 0.65 | 0.108 | 0.752 |
| A0A251SAZ2 | A0A251V6D2 | 4232.A0A251SAZ2 | 4232.A0A251V6D2 | 0 | 0.144 | 0.665 | 0.251 | 0.766 |
| A0A251SAZ2 | SPK-2-2 | 4232.A0A251SAZ2 | 4232.A0A251SEN3 | 0 | 0.144 | 0.665 | 0.251 | 0.766 |
| A0A251SAZ2 | A0A251T7F1 | 4232.A0A251SAZ2 | 4232.A0A251T7F1 | 0 | 0.144 | 0.665 | 0.251 | 0.766 |
| A0A251SAZ2 | A0A251VAR3 | 4232.A0A251SAZ2 | 4232.A0A251VAR3 | 0 | 0.144 | 0.665 | 0.364 | 0.802 |
| A0A251SAZ2 | A0A251SWA4 | 4232.A0A251SAZ2 | 4232.A0A251SWA4 | 0 | 0.144 | 0.665 | 0.364 | 0.802 |
| A0A251SAZ2 | SAPK3 | 4232.A0A251SAZ2 | 4232.A0A251VNN9 | 0 | 0.144 | 0.665 | 0.364 | 0.802 |
| A0A251SBW4 | HaPYL8c | 4232.A0A251SBW4 | 4232.A0A251RLE6 | 0 | 0.203 | 0.65 | 0 | 0.709 |
| A0A251SBW4 | OST1 | 4232.A0A251SBW4 | 4232.A0A251RWW9 | 0 | 0.144 | 0.665 | 0.421 | 0.819 |
| A0A251SBW4 | HaPYR1a | 4232.A0A251SBW4 | 4232.A0A251UG74 | 0 | 0.105 | 0.65 | 0.131 | 0.704 |
| A0A251SBW4 | HaPYL8a | 4232.A0A251SBW4 | 4232.A0A251UGS1 | 0 | 0.203 | 0.65 | 0 | 0.709 |
| A0A251SBW4 | HaPYL9a | 4232.A0A251SBW4 | 4232.A0A251V2E7 | 0 | 0.203 | 0.65 | 0 | 0.709 |
| A0A251SBW4 | HaPYL8b | 4232.A0A251SBW4 | 4232.A0A251UE63 | 0 | 0.203 | 0.65 | 0 | 0.709 |
| A0A251SBW4 | HaPYL9b | 4232.A0A251SBW4 | 4232.A0A251UGY0 | 0 | 0.203 | 0.65 | 0 | 0.709 |
| A0A251SBW4 | HaPYL9c | 4232.A0A251SBW4 | 4232.A0A251TWF7 | 0 | 0.203 | 0.65 | 0 | 0.709 |
| A0A251SBW4 | MPK4 | 4232.A0A251SBW4 | 4232.A0A251TTG0 | 0.06 | 0.192 | 0.081 | 0.654 | 0.726 |
| A0A251SBW4 | HaPYL2a | 4232.A0A251SBW4 | 4232.A0A251V933 | 0 | 0.27 | 0.65 | 0.144 | 0.762 |
| A0A251SBW4 | HaPYL2c | 4232.A0A251SBW4 | 4232.A0A251STT1 | 0 | 0.27 | 0.65 | 0.144 | 0.762 |
| A0A251SBW4 | HaPYL2b | 4232.A0A251SBW4 | 4232.A0A251UAP0 | 0 | 0.27 | 0.65 | 0.144 | 0.762 |
| A0A251SBW4 | HaPYL2d | 4232.A0A251SBW4 | 4232.A0A251U5Y4 | 0 | 0.27 | 0.65 | 0.144 | 0.762 |
| A0A251SBW4 | A0A251SL15 | 4232.A0A251SBW4 | 4232.A0A251SL15 | 0 | 0.144 | 0.665 | 0.252 | 0.767 |
| A0A251SBW4 | SNRK2-10 | 4232.A0A251SBW4 | 4232.A0A251TJT8 | 0 | 0.144 | 0.665 | 0.252 | 0.767 |
| A0A251SBW4 | A0A251UJ18 | 4232.A0A251SBW4 | 4232.A0A251UJ18 | 0 | 0.144 | 0.665 | 0.252 | 0.767 |
| A0A251SBW4 | ASK2 | 4232.A0A251SBW4 | 4232.A0A251TBT3 | 0 | 0.144 | 0.665 | 0.252 | 0.767 |
| A0A251SBW4 | A0A251TFU6 | 4232.A0A251SBW4 | 4232.A0A251TFU6 | 0 | 0.144 | 0.665 | 0.302 | 0.782 |
| A0A251SBW4 | A0A251V6D2 | 4232.A0A251SBW4 | 4232.A0A251V6D2 | 0 | 0.144 | 0.665 | 0.421 | 0.819 |
| A0A251SBW4 | A0A251T7F1 | 4232.A0A251SBW4 | 4232.A0A251T7F1 | 0 | 0.144 | 0.665 | 0.421 | 0.819 |
| A0A251SBW4 | SPK-2-2 | 4232.A0A251SBW4 | 4232.A0A251SEN3 | 0 | 0.144 | 0.665 | 0.421 | 0.819 |
| A0A251SBW4 | SAPK3 | 4232.A0A251SBW4 | 4232.A0A251VNN9 | 0 | 0.144 | 0.665 | 0.504 | 0.845 |
| A0A251SBW4 | A0A251VAR3 | 4232.A0A251SBW4 | 4232.A0A251VAR3 | 0 | 0.144 | 0.665 | 0.504 | 0.845 |
| A0A251SBW4 | A0A251SWA4 | 4232.A0A251SBW4 | 4232.A0A251SWA4 | 0 | 0.144 | 0.665 | 0.504 | 0.845 |
| A0A251SL15 | ATPP2CA | 4232.A0A251SL15 | 4232.A0A251TTP8 | 0 | 0.144 | 0.665 | 0.176 | 0.743 |
| A0A251SL15 | A0A251UEA5 | 4232.A0A251SL15 | 4232.A0A251UEA5 | 0 | 0.144 | 0.665 | 0.176 | 0.743 |
| A0A251SL15 | A0A251TP95 | 4232.A0A251SL15 | 4232.A0A251TP95 | 0 | 0.144 | 0.665 | 0.176 | 0.743 |
| A0A251SL15 | A0A251UCZ1 | 4232.A0A251SL15 | 4232.A0A251UCZ1 | 0 | 0.144 | 0.665 | 0.176 | 0.743 |
| A0A251SL15 | A0A251U9L3 | 4232.A0A251SL15 | 4232.A0A251U9L3 | 0 | 0.144 | 0.665 | 0.176 | 0.743 |
| A0A251SL15 | A0A251UN79 | 4232.A0A251SL15 | 4232.A0A251UN79 | 0 | 0.144 | 0.665 | 0.176 | 0.743 |
| A0A251SL15 | A0A251UJ72 | 4232.A0A251SL15 | 4232.A0A251UJ72 | 0 | 0.144 | 0.665 | 0.176 | 0.743 |
| A0A251SL15 | A0A251UCX7 | 4232.A0A251SL15 | 4232.A0A251UCX7 | 0 | 0.144 | 0.665 | 0.176 | 0.743 |
| A0A251SL15 | HAI2 | 4232.A0A251SL15 | 4232.A0A251SSF2 | 0 | 0.144 | 0.665 | 0.202 | 0.751 |
| A0A251SL15 | A0A251UNA0 | 4232.A0A251SL15 | 4232.A0A251UNA0 | 0 | 0.144 | 0.665 | 0.202 | 0.751 |
| A0A251SL15 | A0A251U0K7 | 4232.A0A251SL15 | 4232.A0A251U0K7 | 0 | 0.144 | 0.665 | 0.222 | 0.757 |
| A0A251SL15 | ABI2 | 4232.A0A251SL15 | 4232.A0A251TQT8 | 0 | 0.144 | 0.665 | 0.222 | 0.757 |
| A0A251SL15 | P2C06 | 4232.A0A251SL15 | 4232.A0A251TU99 | 0 | 0.273 | 0.665 | 0.208 | 0.79 |
| A0A251SL15 | A0A251TQ41 | 4232.A0A251SL15 | 4232.A0A251TQ41 | 0 | 0.273 | 0.665 | 0.208 | 0.79 |
| A0A251SL15 | HAB1 | 4232.A0A251SL15 | 4232.A0A251U1I6 | 0 | 0.273 | 0.665 | 0.208 | 0.79 |
| HaPYL4g | A0A251UN79 | 4232.A0A251SL86 | 4232.A0A251UN79 | 0 | 0.259 | 0.65 | 0.08 | 0.74 |
| HaPYL4g | A0A251UJ72 | 4232.A0A251SL86 | 4232.A0A251UJ72 | 0 | 0.259 | 0.65 | 0.08 | 0.74 |
| HaPYL4g | A0A251UEA5 | 4232.A0A251SL86 | 4232.A0A251UEA5 | 0 | 0.259 | 0.65 | 0.08 | 0.74 |
| HaPYL4g | A0A251TP95 | 4232.A0A251SL86 | 4232.A0A251TP95 | 0 | 0.259 | 0.65 | 0.08 | 0.74 |
| HaPYL4g | A0A251UCZ1 | 4232.A0A251SL86 | 4232.A0A251UCZ1 | 0 | 0.259 | 0.65 | 0.08 | 0.74 |
| HaPYL4g | A0A251U9L3 | 4232.A0A251SL86 | 4232.A0A251U9L3 | 0 | 0.259 | 0.65 | 0.08 | 0.74 |
| HaPYL4g | ATPP2CA | 4232.A0A251SL86 | 4232.A0A251TTP8 | 0 | 0.259 | 0.65 | 0.08 | 0.74 |
| HaPYL4g | A0A251UCX7 | 4232.A0A251SL86 | 4232.A0A251UCX7 | 0 | 0.259 | 0.65 | 0.08 | 0.74 |
| HaPYL4g | P2C06 | 4232.A0A251SL86 | 4232.A0A251TU99 | 0 | 0.273 | 0.65 | 0.181 | 0.773 |
| HaPYL4g | A0A251TQ41 | 4232.A0A251SL86 | 4232.A0A251TQ41 | 0 | 0.273 | 0.65 | 0.181 | 0.773 |
| HaPYL4g | HAB1 | 4232.A0A251SL86 | 4232.A0A251U1I6 | 0 | 0.273 | 0.65 | 0.181 | 0.773 |
| HaPYL2c | HAI2 | 4232.A0A251STT1 | 4232.A0A251SSF2 | 0 | 0.27 | 0.65 | 0.108 | 0.752 |
| HaPYL2c | ABI2 | 4232.A0A251STT1 | 4232.A0A251TQT8 | 0 | 0.27 | 0.65 | 0 | 0.733 |
| HaPYL2c | A0A251U0K7 | 4232.A0A251STT1 | 4232.A0A251U0K7 | 0 | 0.27 | 0.65 | 0 | 0.733 |
| HaPYL2c | A0A251UNA0 | 4232.A0A251STT1 | 4232.A0A251UNA0 | 0 | 0.27 | 0.65 | 0.108 | 0.752 |
| HaPYL2c | A0A251TSF6 | 4232.A0A251STT1 | 4232.A0A251TSF6 | 0 | 0.68 | 0 | 0.262 | 0.753 |
| HaPYL2c | A0A251U9L3 | 4232.A0A251STT1 | 4232.A0A251U9L3 | 0 | 0.322 | 0.65 | 0.085 | 0.763 |
| HaPYL2c | A0A251UCZ1 | 4232.A0A251STT1 | 4232.A0A251UCZ1 | 0 | 0.322 | 0.65 | 0.085 | 0.763 |
| HaPYL2c | ATPP2CA | 4232.A0A251STT1 | 4232.A0A251TTP8 | 0 | 0.322 | 0.65 | 0.085 | 0.763 |
| HaPYL2c | A0A251UCX7 | 4232.A0A251STT1 | 4232.A0A251UCX7 | 0 | 0.322 | 0.65 | 0.085 | 0.763 |
| HaPYL2c | A0A251UJ72 | 4232.A0A251STT1 | 4232.A0A251UJ72 | 0 | 0.322 | 0.65 | 0.085 | 0.763 |
| HaPYL2c | A0A251TP95 | 4232.A0A251STT1 | 4232.A0A251TP95 | 0 | 0.322 | 0.65 | 0.085 | 0.763 |
| HaPYL2c | A0A251UEA5 | 4232.A0A251STT1 | 4232.A0A251UEA5 | 0 | 0.322 | 0.65 | 0.085 | 0.763 |
| HaPYL2c | A0A251UN79 | 4232.A0A251STT1 | 4232.A0A251UN79 | 0 | 0.322 | 0.65 | 0.085 | 0.763 |
| HaPYL2c | HAB1 | 4232.A0A251STT1 | 4232.A0A251U1I6 | 0 | 0.68 | 0.65 | 0.262 | 0.91 |
| HaPYL2c | P2C06 | 4232.A0A251STT1 | 4232.A0A251TU99 | 0 | 0.68 | 0.65 | 0.262 | 0.91 |
| HaPYL2c | A0A251TQ41 | 4232.A0A251STT1 | 4232.A0A251TQ41 | 0 | 0.68 | 0.65 | 0.262 | 0.91 |
| A0A251SWA4 | HAI2 | 4232.A0A251SWA4 | 4232.A0A251SSF2 | 0 | 0.144 | 0.665 | 0.364 | 0.802 |
| A0A251SWA4 | A0A251UEA5 | 4232.A0A251SWA4 | 4232.A0A251UEA5 | 0 | 0.144 | 0.665 | 0.176 | 0.743 |
| A0A251SWA4 | A0A251TP95 | 4232.A0A251SWA4 | 4232.A0A251TP95 | 0 | 0.144 | 0.665 | 0.176 | 0.743 |
| A0A251SWA4 | A0A251UCZ1 | 4232.A0A251SWA4 | 4232.A0A251UCZ1 | 0 | 0.144 | 0.665 | 0.176 | 0.743 |
| A0A251SWA4 | A0A251U9L3 | 4232.A0A251SWA4 | 4232.A0A251U9L3 | 0 | 0.144 | 0.665 | 0.176 | 0.743 |
| A0A251SWA4 | A0A251UJ72 | 4232.A0A251SWA4 | 4232.A0A251UJ72 | 0 | 0.144 | 0.665 | 0.176 | 0.743 |
| A0A251SWA4 | A0A251UN79 | 4232.A0A251SWA4 | 4232.A0A251UN79 | 0 | 0.144 | 0.665 | 0.176 | 0.743 |
| A0A251SWA4 | A0A251UCX7 | 4232.A0A251SWA4 | 4232.A0A251UCX7 | 0 | 0.144 | 0.665 | 0.176 | 0.743 |
| A0A251SWA4 | ATPP2CA | 4232.A0A251SWA4 | 4232.A0A251TTP8 | 0 | 0.144 | 0.665 | 0.176 | 0.743 |
| A0A251SWA4 | A0A251UNA0 | 4232.A0A251SWA4 | 4232.A0A251UNA0 | 0 | 0.144 | 0.665 | 0.364 | 0.802 |
| A0A251SWA4 | A0A251U0K7 | 4232.A0A251SWA4 | 4232.A0A251U0K7 | 0 | 0.144 | 0.665 | 0.373 | 0.804 |
| A0A251SWA4 | ABI2 | 4232.A0A251SWA4 | 4232.A0A251TQT8 | 0 | 0.144 | 0.665 | 0.373 | 0.804 |
| A0A251SWA4 | A0A251TQ41 | 4232.A0A251SWA4 | 4232.A0A251TQ41 | 0 | 0.394 | 0.665 | 0.308 | 0.847 |
| A0A251SWA4 | HAB1 | 4232.A0A251SWA4 | 4232.A0A251U1I6 | 0 | 0.394 | 0.665 | 0.308 | 0.847 |
| A0A251SWA4 | P2C06 | 4232.A0A251SWA4 | 4232.A0A251TU99 | 0 | 0.394 | 0.665 | 0.308 | 0.847 |
| A0A251T7F1 | HAI2 | 4232.A0A251T7F1 | 4232.A0A251SSF2 | 0 | 0.144 | 0.665 | 0.251 | 0.766 |
| A0A251T7F1 | A0A251TSF6 | 4232.A0A251T7F1 | 4232.A0A251TSF6 | 0 | 0.623 | 0.084 | 0.252 | 0.719 |
| A0A251T7F1 | A0A251U9L3 | 4232.A0A251T7F1 | 4232.A0A251U9L3 | 0 | 0.144 | 0.665 | 0.153 | 0.736 |
| A0A251T7F1 | A0A251UCZ1 | 4232.A0A251T7F1 | 4232.A0A251UCZ1 | 0 | 0.144 | 0.665 | 0.153 | 0.736 |
| A0A251T7F1 | A0A251TP95 | 4232.A0A251T7F1 | 4232.A0A251TP95 | 0 | 0.144 | 0.665 | 0.153 | 0.736 |
| A0A251T7F1 | A0A251UEA5 | 4232.A0A251T7F1 | 4232.A0A251UEA5 | 0 | 0.144 | 0.665 | 0.153 | 0.736 |
| A0A251T7F1 | A0A251UCX7 | 4232.A0A251T7F1 | 4232.A0A251UCX7 | 0 | 0.144 | 0.665 | 0.153 | 0.736 |
| A0A251T7F1 | A0A251UN79 | 4232.A0A251T7F1 | 4232.A0A251UN79 | 0 | 0.144 | 0.665 | 0.153 | 0.736 |
| A0A251T7F1 | A0A251UJ72 | 4232.A0A251T7F1 | 4232.A0A251UJ72 | 0 | 0.144 | 0.665 | 0.153 | 0.736 |
| A0A251T7F1 | ATPP2CA | 4232.A0A251T7F1 | 4232.A0A251TTP8 | 0 | 0.144 | 0.665 | 0.153 | 0.736 |
| A0A251T7F1 | A0A251UNA0 | 4232.A0A251T7F1 | 4232.A0A251UNA0 | 0 | 0.144 | 0.665 | 0.251 | 0.766 |
| A0A251T7F1 | ABI2 | 4232.A0A251T7F1 | 4232.A0A251TQT8 | 0 | 0.144 | 0.665 | 0.302 | 0.782 |
| A0A251T7F1 | A0A251U0K7 | 4232.A0A251T7F1 | 4232.A0A251U0K7 | 0 | 0.144 | 0.665 | 0.302 | 0.782 |
| A0A251T7F1 | A0A251TQ41 | 4232.A0A251T7F1 | 4232.A0A251TQ41 | 0 | 0.623 | 0.665 | 0.252 | 0.897 |
| A0A251T7F1 | HAB1 | 4232.A0A251T7F1 | 4232.A0A251U1I6 | 0 | 0.623 | 0.665 | 0.252 | 0.897 |
| A0A251T7F1 | P2C06 | 4232.A0A251T7F1 | 4232.A0A251TU99 | 0 | 0.623 | 0.665 | 0.252 | 0.897 |
| A0A251TFU6 | HAI2 | 4232.A0A251TFU6 | 4232.A0A251SSF2 | 0 | 0.144 | 0.665 | 0.185 | 0.746 |
| A0A251TFU6 | ATPP2CA | 4232.A0A251TFU6 | 4232.A0A251TTP8 | 0 | 0.144 | 0.665 | 0.143 | 0.733 |
| A0A251TFU6 | A0A251UCX7 | 4232.A0A251TFU6 | 4232.A0A251UCX7 | 0 | 0.144 | 0.665 | 0.143 | 0.733 |
| A0A251TFU6 | A0A251TP95 | 4232.A0A251TFU6 | 4232.A0A251TP95 | 0 | 0.144 | 0.665 | 0.143 | 0.733 |
| A0A251TFU6 | A0A251UEA5 | 4232.A0A251TFU6 | 4232.A0A251UEA5 | 0 | 0.144 | 0.665 | 0.143 | 0.733 |
| A0A251TFU6 | A0A251UCZ1 | 4232.A0A251TFU6 | 4232.A0A251UCZ1 | 0 | 0.144 | 0.665 | 0.143 | 0.733 |
| A0A251TFU6 | ABI2 | 4232.A0A251TFU6 | 4232.A0A251TQT8 | 0 | 0.144 | 0.665 | 0.182 | 0.745 |
| A0A251TFU6 | A0A251U0K7 | 4232.A0A251TFU6 | 4232.A0A251U0K7 | 0 | 0.144 | 0.665 | 0.182 | 0.745 |
| A0A251TFU6 | A0A251UNA0 | 4232.A0A251TFU6 | 4232.A0A251UNA0 | 0 | 0.144 | 0.665 | 0.185 | 0.746 |
| A0A251TFU6 | A0A251UJ72 | 4232.A0A251TFU6 | 4232.A0A251UJ72 | 0 | 0.144 | 0.665 | 0.214 | 0.755 |
| A0A251TFU6 | A0A251UN79 | 4232.A0A251TFU6 | 4232.A0A251UN79 | 0 | 0.144 | 0.665 | 0.214 | 0.755 |
| A0A251TFU6 | A0A251U9L3 | 4232.A0A251TFU6 | 4232.A0A251U9L3 | 0 | 0.144 | 0.665 | 0.214 | 0.755 |
| A0A251TFU6 | P2C06 | 4232.A0A251TFU6 | 4232.A0A251TU99 | 0 | 0.394 | 0.665 | 0.172 | 0.817 |
| A0A251TFU6 | HAB1 | 4232.A0A251TFU6 | 4232.A0A251U1I6 | 0 | 0.394 | 0.665 | 0.226 | 0.829 |
| A0A251TFU6 | A0A251TQ41 | 4232.A0A251TFU6 | 4232.A0A251TQ41 | 0 | 0.394 | 0.665 | 0.226 | 0.829 |
| HaPYL4f | A0A251UJ72 | 4232.A0A251TKA1 | 4232.A0A251UJ72 | 0 | 0.259 | 0.65 | 0.08 | 0.74 |
| HaPYL4f | A0A251UN79 | 4232.A0A251TKA1 | 4232.A0A251UN79 | 0 | 0.259 | 0.65 | 0.08 | 0.74 |
| HaPYL4f | A0A251U9L3 | 4232.A0A251TKA1 | 4232.A0A251U9L3 | 0 | 0.259 | 0.65 | 0.08 | 0.74 |
| HaPYL4f | A0A251TP95 | 4232.A0A251TKA1 | 4232.A0A251TP95 | 0 | 0.259 | 0.65 | 0.086 | 0.742 |
| HaPYL4f | A0A251UEA5 | 4232.A0A251TKA1 | 4232.A0A251UEA5 | 0 | 0.259 | 0.65 | 0.086 | 0.742 |
| HaPYL4f | ATPP2CA | 4232.A0A251TKA1 | 4232.A0A251TTP8 | 0 | 0.259 | 0.65 | 0.086 | 0.742 |
| HaPYL4f | A0A251UCX7 | 4232.A0A251TKA1 | 4232.A0A251UCX7 | 0 | 0.259 | 0.65 | 0.086 | 0.742 |
| HaPYL4f | A0A251UCZ1 | 4232.A0A251TKA1 | 4232.A0A251UCZ1 | 0 | 0.259 | 0.65 | 0.086 | 0.742 |
| HaPYL4f | HAB1 | 4232.A0A251TKA1 | 4232.A0A251U1I6 | 0 | 0.273 | 0.65 | 0.181 | 0.773 |
| HaPYL4f | P2C06 | 4232.A0A251TKA1 | 4232.A0A251TU99 | 0 | 0.273 | 0.65 | 0.181 | 0.773 |
| HaPYL4f | A0A251TQ41 | 4232.A0A251TKA1 | 4232.A0A251TQ41 | 0 | 0.273 | 0.65 | 0.181 | 0.773 |
| A0A251TKH2 | HAB1 | 4232.A0A251TKH2 | 4232.A0A251U1I6 | 0 | 0.623 | 0.084 | 0.252 | 0.719 |
| A0A251TKH2 | A0A251TQ41 | 4232.A0A251TKH2 | 4232.A0A251TQ41 | 0 | 0.623 | 0.084 | 0.252 | 0.719 |
| A0A251TKH2 | A0A251TSF6 | 4232.A0A251TKH2 | 4232.A0A251TSF6 | 0 | 0.623 | 0.084 | 0.252 | 0.719 |
| A0A251TKH2 | P2C06 | 4232.A0A251TKH2 | 4232.A0A251TU99 | 0 | 0.623 | 0.084 | 0.252 | 0.719 |
| A0A251TP95 | HaPYL8c | 4232.A0A251TP95 | 4232.A0A251RLE6 | 0 | 0.203 | 0.65 | 0.08 | 0.72 |
| A0A251TP95 | HaPYL4b | 4232.A0A251TP95 | 4232.A0A251RSA8 | 0 | 0.259 | 0.65 | 0.08 | 0.74 |
| A0A251TP95 | OST1 | 4232.A0A251TP95 | 4232.A0A251RWW9 | 0 | 0.144 | 0.665 | 0.153 | 0.736 |
| A0A251TP95 | SPK-2-2 | 4232.A0A251TP95 | 4232.A0A251SEN3 | 0 | 0.144 | 0.665 | 0.153 | 0.736 |
| A0A251TP95 | ASK2 | 4232.A0A251TP95 | 4232.A0A251TBT3 | 0 | 0.144 | 0.665 | 0.176 | 0.743 |
| A0A251TP95 | SNRK2-10 | 4232.A0A251TP95 | 4232.A0A251TJT8 | 0 | 0.144 | 0.665 | 0.176 | 0.743 |
| A0A251TP95 | HaPYL8a | 4232.A0A251TP95 | 4232.A0A251UGS1 | 0 | 0.203 | 0.65 | 0.08 | 0.72 |
| A0A251TP95 | HaPYL9a | 4232.A0A251TP95 | 4232.A0A251V2E7 | 0 | 0.203 | 0.65 | 0.08 | 0.72 |
| A0A251TP95 | HaPYL8b | 4232.A0A251TP95 | 4232.A0A251UE63 | 0 | 0.203 | 0.65 | 0.08 | 0.72 |
| A0A251TP95 | HaPYL9b | 4232.A0A251TP95 | 4232.A0A251UGY0 | 0 | 0.203 | 0.65 | 0.08 | 0.72 |
| A0A251TP95 | HaPYL9c | 4232.A0A251TP95 | 4232.A0A251TWF7 | 0 | 0.203 | 0.65 | 0.08 | 0.72 |
| A0A251TP95 | MPK4 | 4232.A0A251TP95 | 4232.A0A251TTG0 | 0.06 | 0.192 | 0.081 | 0.654 | 0.726 |
| A0A251TP95 | A0A251V6D2 | 4232.A0A251TP95 | 4232.A0A251V6D2 | 0 | 0.144 | 0.665 | 0.153 | 0.736 |
| A0A251TP95 | HaPYL4c | 4232.A0A251TP95 | 4232.A0A251UDF9 | 0 | 0.259 | 0.65 | 0.08 | 0.74 |
| A0A251TP95 | HaPYL4a | 4232.A0A251TP95 | 4232.A0A251TY59 | 0 | 0.259 | 0.65 | 0.086 | 0.742 |
| A0A251TP95 | SAPK3 | 4232.A0A251TP95 | 4232.A0A251VNN9 | 0 | 0.144 | 0.665 | 0.176 | 0.743 |
| A0A251TP95 | A0A251VAR3 | 4232.A0A251TP95 | 4232.A0A251VAR3 | 0 | 0.144 | 0.665 | 0.176 | 0.743 |
| A0A251TP95 | A0A251UJ18 | 4232.A0A251TP95 | 4232.A0A251UJ18 | 0 | 0.144 | 0.665 | 0.176 | 0.743 |
| A0A251TP95 | HaPYL2a | 4232.A0A251TP95 | 4232.A0A251V933 | 0 | 0.322 | 0.65 | 0.085 | 0.763 |
| A0A251TP95 | HaPYL2b | 4232.A0A251TP95 | 4232.A0A251UAP0 | 0 | 0.322 | 0.65 | 0.085 | 0.763 |
| A0A251TP95 | HaPYL2d | 4232.A0A251TP95 | 4232.A0A251U5Y4 | 0 | 0.322 | 0.65 | 0.085 | 0.763 |
| A0A251TP95 | HaPYR1a | 4232.A0A251TP95 | 4232.A0A251UG74 | 0 | 0.395 | 0.65 | 0.146 | 0.803 |
| A0A251TQ41 | HaPYL8c | 4232.A0A251TQ41 | 4232.A0A251RLE6 | 0 | 0.621 | 0.65 | 0.181 | 0.881 |
| A0A251TQ41 | HaPYL4b | 4232.A0A251TQ41 | 4232.A0A251RSA8 | 0 | 0.273 | 0.65 | 0.181 | 0.773 |
| A0A251TQ41 | OST1 | 4232.A0A251TQ41 | 4232.A0A251RWW9 | 0 | 0.623 | 0.665 | 0.252 | 0.897 |
| A0A251TQ41 | SPK-2-2 | 4232.A0A251TQ41 | 4232.A0A251SEN3 | 0 | 0.623 | 0.665 | 0.252 | 0.897 |
| A0A251TQ41 | ASK2 | 4232.A0A251TQ41 | 4232.A0A251TBT3 | 0 | 0.273 | 0.665 | 0.208 | 0.79 |
| A0A251TQ41 | SNRK2-10 | 4232.A0A251TQ41 | 4232.A0A251TJT8 | 0 | 0.273 | 0.665 | 0.208 | 0.79 |
| A0A251TQ41 | MPK4 | 4232.A0A251TQ41 | 4232.A0A251TTG0 | 0.06 | 0.192 | 0.081 | 0.654 | 0.726 |
| A0A251TQ41 | HaPYL4c | 4232.A0A251TQ41 | 4232.A0A251UDF9 | 0 | 0.273 | 0.65 | 0.181 | 0.773 |
| A0A251TQ41 | HaPYL4a | 4232.A0A251TQ41 | 4232.A0A251TY59 | 0 | 0.273 | 0.65 | 0.181 | 0.773 |
| A0A251TQ41 | A0A251UJ18 | 4232.A0A251TQ41 | 4232.A0A251UJ18 | 0 | 0.273 | 0.665 | 0.208 | 0.79 |
| A0A251TQ41 | A0A251VAR3 | 4232.A0A251TQ41 | 4232.A0A251VAR3 | 0 | 0.394 | 0.665 | 0.308 | 0.847 |
| A0A251TQ41 | SAPK3 | 4232.A0A251TQ41 | 4232.A0A251VNN9 | 0 | 0.394 | 0.665 | 0.308 | 0.847 |
| A0A251TQ41 | HaPYR1a | 4232.A0A251TQ41 | 4232.A0A251UG74 | 0 | 0.477 | 0.65 | 0.311 | 0.862 |
| A0A251TQ41 | HaPYL9c | 4232.A0A251TQ41 | 4232.A0A251TWF7 | 0 | 0.621 | 0.65 | 0.181 | 0.881 |
| A0A251TQ41 | HaPYL8b | 4232.A0A251TQ41 | 4232.A0A251UE63 | 0 | 0.621 | 0.65 | 0.181 | 0.881 |
| A0A251TQ41 | HaPYL9b | 4232.A0A251TQ41 | 4232.A0A251UGY0 | 0 | 0.621 | 0.65 | 0.181 | 0.881 |
| A0A251TQ41 | HaPYL8a | 4232.A0A251TQ41 | 4232.A0A251UGS1 | 0 | 0.621 | 0.65 | 0.181 | 0.881 |
| A0A251TQ41 | HaPYL9a | 4232.A0A251TQ41 | 4232.A0A251V2E7 | 0 | 0.621 | 0.65 | 0.181 | 0.881 |
| A0A251TQ41 | A0A251V6D2 | 4232.A0A251TQ41 | 4232.A0A251V6D2 | 0 | 0.623 | 0.665 | 0.252 | 0.897 |
| A0A251TQ41 | HaPYL2d | 4232.A0A251TQ41 | 4232.A0A251U5Y4 | 0 | 0.68 | 0.65 | 0.262 | 0.91 |
| A0A251TQ41 | HaPYL2b | 4232.A0A251TQ41 | 4232.A0A251UAP0 | 0 | 0.68 | 0.65 | 0.262 | 0.91 |
| A0A251TQ41 | HaPYL2a | 4232.A0A251TQ41 | 4232.A0A251V933 | 0 | 0.68 | 0.65 | 0.262 | 0.91 |
| A0A251TSF6 | OST1 | 4232.A0A251TSF6 | 4232.A0A251RWW9 | 0 | 0.623 | 0.084 | 0.252 | 0.719 |
| A0A251TSF6 | SPK-2-2 | 4232.A0A251TSF6 | 4232.A0A251SEN3 | 0 | 0.623 | 0.084 | 0.252 | 0.719 |
| A0A251TSF6 | A0A251V6D2 | 4232.A0A251TSF6 | 4232.A0A251V6D2 | 0 | 0.623 | 0.084 | 0.252 | 0.719 |
| A0A251TSF6 | HaPYL2d | 4232.A0A251TSF6 | 4232.A0A251U5Y4 | 0 | 0.68 | 0 | 0.262 | 0.753 |
| A0A251TSF6 | HaPYL2b | 4232.A0A251TSF6 | 4232.A0A251UAP0 | 0 | 0.68 | 0 | 0.262 | 0.753 |
| A0A251TSF6 | HaPYL2a | 4232.A0A251TSF6 | 4232.A0A251V933 | 0 | 0.68 | 0 | 0.262 | 0.753 |
| HaPYL9c | HAI2 | 4232.A0A251TWF7 | 4232.A0A251SSF2 | 0 | 0.203 | 0.65 | 0.153 | 0.743 |
| HaPYL9c | ABI2 | 4232.A0A251TWF7 | 4232.A0A251TQT8 | 0 | 0.203 | 0.65 | 0 | 0.709 |
| HaPYL9c | ATPP2CA | 4232.A0A251TWF7 | 4232.A0A251TTP8 | 0 | 0.203 | 0.65 | 0.08 | 0.72 |
| HaPYL9c | P2C06 | 4232.A0A251TWF7 | 4232.A0A251TU99 | 0 | 0.621 | 0.65 | 0.181 | 0.881 |
| HaPYL9c | A0A251U0K7 | 4232.A0A251TWF7 | 4232.A0A251U0K7 | 0 | 0.203 | 0.65 | 0 | 0.709 |
| HaPYL9c | A0A251UCZ1 | 4232.A0A251TWF7 | 4232.A0A251UCZ1 | 0 | 0.203 | 0.65 | 0.08 | 0.72 |
| HaPYL9c | A0A251UJ72 | 4232.A0A251TWF7 | 4232.A0A251UJ72 | 0 | 0.203 | 0.65 | 0.08 | 0.72 |
| HaPYL9c | A0A251UEA5 | 4232.A0A251TWF7 | 4232.A0A251UEA5 | 0 | 0.203 | 0.65 | 0.08 | 0.72 |
| HaPYL9c | A0A251UN79 | 4232.A0A251TWF7 | 4232.A0A251UN79 | 0 | 0.203 | 0.65 | 0.08 | 0.72 |
| HaPYL9c | A0A251U9L3 | 4232.A0A251TWF7 | 4232.A0A251U9L3 | 0 | 0.203 | 0.65 | 0.08 | 0.72 |
| HaPYL9c | A0A251UCX7 | 4232.A0A251TWF7 | 4232.A0A251UCX7 | 0 | 0.203 | 0.65 | 0.08 | 0.72 |
| HaPYL9c | A0A251UNA0 | 4232.A0A251TWF7 | 4232.A0A251UNA0 | 0 | 0.203 | 0.65 | 0.153 | 0.743 |
| HaPYL9c | HAB1 | 4232.A0A251TWF7 | 4232.A0A251U1I6 | 0 | 0.621 | 0.65 | 0.181 | 0.881 |
| HaPYL4a | ATPP2CA | 4232.A0A251TY59 | 4232.A0A251TTP8 | 0 | 0.259 | 0.65 | 0.086 | 0.742 |
| HaPYL4a | P2C06 | 4232.A0A251TY59 | 4232.A0A251TU99 | 0 | 0.273 | 0.65 | 0.181 | 0.773 |
| HaPYL4a | A0A251UN79 | 4232.A0A251TY59 | 4232.A0A251UN79 | 0 | 0.259 | 0.65 | 0.08 | 0.74 |
| HaPYL4a | A0A251UJ72 | 4232.A0A251TY59 | 4232.A0A251UJ72 | 0 | 0.259 | 0.65 | 0.08 | 0.74 |
| HaPYL4a | A0A251U9L3 | 4232.A0A251TY59 | 4232.A0A251U9L3 | 0 | 0.259 | 0.65 | 0.08 | 0.74 |
| HaPYL4a | A0A251UEA5 | 4232.A0A251TY59 | 4232.A0A251UEA5 | 0 | 0.259 | 0.65 | 0.086 | 0.742 |
| HaPYL4a | A0A251UCZ1 | 4232.A0A251TY59 | 4232.A0A251UCZ1 | 0 | 0.259 | 0.65 | 0.086 | 0.742 |
| HaPYL4a | A0A251UCX7 | 4232.A0A251TY59 | 4232.A0A251UCX7 | 0 | 0.259 | 0.65 | 0.086 | 0.742 |
| HaPYL4a | HAB1 | 4232.A0A251TY59 | 4232.A0A251U1I6 | 0 | 0.273 | 0.65 | 0.181 | 0.773 |
| A0A251U0K7 | HaPYL8c | 4232.A0A251U0K7 | 4232.A0A251RLE6 | 0 | 0.203 | 0.65 | 0 | 0.709 |
| A0A251U0K7 | OST1 | 4232.A0A251U0K7 | 4232.A0A251RWW9 | 0 | 0.144 | 0.665 | 0.302 | 0.782 |
| A0A251U0K7 | SPK-2-2 | 4232.A0A251U0K7 | 4232.A0A251SEN3 | 0 | 0.144 | 0.665 | 0.302 | 0.782 |
| A0A251U0K7 | ASK2 | 4232.A0A251U0K7 | 4232.A0A251TBT3 | 0 | 0.144 | 0.665 | 0.222 | 0.757 |
| A0A251U0K7 | SNRK2-10 | 4232.A0A251U0K7 | 4232.A0A251TJT8 | 0 | 0.144 | 0.665 | 0.222 | 0.757 |
| A0A251U0K7 | MPK4 | 4232.A0A251U0K7 | 4232.A0A251TTG0 | 0.06 | 0.192 | 0.081 | 0.654 | 0.726 |
| A0A251U0K7 | HaPYL8b | 4232.A0A251U0K7 | 4232.A0A251UE63 | 0 | 0.203 | 0.65 | 0 | 0.709 |
| A0A251U0K7 | HaPYL9b | 4232.A0A251U0K7 | 4232.A0A251UGY0 | 0 | 0.203 | 0.65 | 0 | 0.709 |
| A0A251U0K7 | HaPYL8a | 4232.A0A251U0K7 | 4232.A0A251UGS1 | 0 | 0.203 | 0.65 | 0 | 0.709 |
| A0A251U0K7 | HaPYL9a | 4232.A0A251U0K7 | 4232.A0A251V2E7 | 0 | 0.203 | 0.65 | 0 | 0.709 |
| A0A251U0K7 | HaPYR1a | 4232.A0A251U0K7 | 4232.A0A251UG74 | 0 | 0.105 | 0.65 | 0.191 | 0.724 |
| A0A251U0K7 | HaPYL2a | 4232.A0A251U0K7 | 4232.A0A251V933 | 0 | 0.27 | 0.65 | 0 | 0.733 |
| A0A251U0K7 | HaPYL2d | 4232.A0A251U0K7 | 4232.A0A251U5Y4 | 0 | 0.27 | 0.65 | 0 | 0.733 |
| A0A251U0K7 | HaPYL2b | 4232.A0A251U0K7 | 4232.A0A251UAP0 | 0 | 0.27 | 0.65 | 0 | 0.733 |
| A0A251U0K7 | A0A251UJ18 | 4232.A0A251U0K7 | 4232.A0A251UJ18 | 0 | 0.144 | 0.665 | 0.222 | 0.757 |
| A0A251U0K7 | A0A251V6D2 | 4232.A0A251U0K7 | 4232.A0A251V6D2 | 0 | 0.144 | 0.665 | 0.302 | 0.782 |
| A0A251U0K7 | A0A251VAR3 | 4232.A0A251U0K7 | 4232.A0A251VAR3 | 0 | 0.144 | 0.665 | 0.373 | 0.804 |
| A0A251U0K7 | SAPK3 | 4232.A0A251U0K7 | 4232.A0A251VNN9 | 0 | 0.144 | 0.665 | 0.373 | 0.804 |
| HaPYL2d | HAI2 | 4232.A0A251U5Y4 | 4232.A0A251SSF2 | 0 | 0.27 | 0.65 | 0.108 | 0.752 |
| HaPYL2d | ABI2 | 4232.A0A251U5Y4 | 4232.A0A251TQT8 | 0 | 0.27 | 0.65 | 0 | 0.733 |
| HaPYL2d | ATPP2CA | 4232.A0A251U5Y4 | 4232.A0A251TTP8 | 0 | 0.322 | 0.65 | 0.085 | 0.763 |
| HaPYL2d | P2C06 | 4232.A0A251U5Y4 | 4232.A0A251TU99 | 0 | 0.68 | 0.65 | 0.262 | 0.91 |
| HaPYL2d | HAB1 | 4232.A0A251U5Y4 | 4232.A0A251U1I6 | 0 | 0.68 | 0.65 | 0.262 | 0.91 |
| HaPYL2d | A0A251UNA0 | 4232.A0A251U5Y4 | 4232.A0A251UNA0 | 0 | 0.27 | 0.65 | 0.108 | 0.752 |
| HaPYL2d | A0A251UCZ1 | 4232.A0A251U5Y4 | 4232.A0A251UCZ1 | 0 | 0.322 | 0.65 | 0.085 | 0.763 |
| HaPYL2d | A0A251U9L3 | 4232.A0A251U5Y4 | 4232.A0A251U9L3 | 0 | 0.322 | 0.65 | 0.085 | 0.763 |
| HaPYL2d | A0A251UN79 | 4232.A0A251U5Y4 | 4232.A0A251UN79 | 0 | 0.322 | 0.65 | 0.085 | 0.763 |
| HaPYL2d | A0A251UEA5 | 4232.A0A251U5Y4 | 4232.A0A251UEA5 | 0 | 0.322 | 0.65 | 0.085 | 0.763 |
| HaPYL2d | A0A251UCX7 | 4232.A0A251U5Y4 | 4232.A0A251UCX7 | 0 | 0.322 | 0.65 | 0.085 | 0.763 |
| HaPYL2d | A0A251UJ72 | 4232.A0A251U5Y4 | 4232.A0A251UJ72 | 0 | 0.322 | 0.65 | 0.085 | 0.763 |
| A0A251U9L3 | HaPYL8c | 4232.A0A251U9L3 | 4232.A0A251RLE6 | 0 | 0.203 | 0.65 | 0.08 | 0.72 |
| A0A251U9L3 | HaPYL4b | 4232.A0A251U9L3 | 4232.A0A251RSA8 | 0 | 0.259 | 0.65 | 0.08 | 0.74 |
| A0A251U9L3 | OST1 | 4232.A0A251U9L3 | 4232.A0A251RWW9 | 0 | 0.144 | 0.665 | 0.153 | 0.736 |
| A0A251U9L3 | SPK-2-2 | 4232.A0A251U9L3 | 4232.A0A251SEN3 | 0 | 0.144 | 0.665 | 0.153 | 0.736 |
| A0A251U9L3 | ASK2 | 4232.A0A251U9L3 | 4232.A0A251TBT3 | 0 | 0.144 | 0.665 | 0.176 | 0.743 |
| A0A251U9L3 | SNRK2-10 | 4232.A0A251U9L3 | 4232.A0A251TJT8 | 0 | 0.144 | 0.665 | 0.176 | 0.743 |
| A0A251U9L3 | MPK4 | 4232.A0A251U9L3 | 4232.A0A251TTG0 | 0.06 | 0.192 | 0.081 | 0.654 | 0.726 |
| A0A251U9L3 | HaPYL9b | 4232.A0A251U9L3 | 4232.A0A251UGY0 | 0 | 0.203 | 0.65 | 0.08 | 0.72 |
| A0A251U9L3 | HaPYL8b | 4232.A0A251U9L3 | 4232.A0A251UE63 | 0 | 0.203 | 0.65 | 0.08 | 0.72 |
| A0A251U9L3 | HaPYL9a | 4232.A0A251U9L3 | 4232.A0A251V2E7 | 0 | 0.203 | 0.65 | 0.08 | 0.72 |
| A0A251U9L3 | HaPYL8a | 4232.A0A251U9L3 | 4232.A0A251UGS1 | 0 | 0.203 | 0.65 | 0.08 | 0.72 |
| A0A251U9L3 | A0A251V6D2 | 4232.A0A251U9L3 | 4232.A0A251V6D2 | 0 | 0.144 | 0.665 | 0.153 | 0.736 |
| A0A251U9L3 | HaPYL4c | 4232.A0A251U9L3 | 4232.A0A251UDF9 | 0 | 0.259 | 0.65 | 0.08 | 0.74 |
| A0A251U9L3 | A0A251VAR3 | 4232.A0A251U9L3 | 4232.A0A251VAR3 | 0 | 0.144 | 0.665 | 0.176 | 0.743 |
| A0A251U9L3 | SAPK3 | 4232.A0A251U9L3 | 4232.A0A251VNN9 | 0 | 0.144 | 0.665 | 0.176 | 0.743 |
| A0A251U9L3 | A0A251UJ18 | 4232.A0A251U9L3 | 4232.A0A251UJ18 | 0 | 0.144 | 0.665 | 0.176 | 0.743 |
| A0A251U9L3 | HaPYL2a | 4232.A0A251U9L3 | 4232.A0A251V933 | 0 | 0.322 | 0.65 | 0.085 | 0.763 |
| A0A251U9L3 | HaPYL2b | 4232.A0A251U9L3 | 4232.A0A251UAP0 | 0 | 0.322 | 0.65 | 0.085 | 0.763 |
| A0A251U9L3 | HaPYR1a | 4232.A0A251U9L3 | 4232.A0A251UG74 | 0 | 0.395 | 0.65 | 0.146 | 0.803 |
| HaPYL2b | HAI2 | 4232.A0A251UAP0 | 4232.A0A251SSF2 | 0 | 0.27 | 0.65 | 0.108 | 0.752 |
| HaPYL2b | ABI2 | 4232.A0A251UAP0 | 4232.A0A251TQT8 | 0 | 0.27 | 0.65 | 0 | 0.733 |
| HaPYL2b | ATPP2CA | 4232.A0A251UAP0 | 4232.A0A251TTP8 | 0 | 0.322 | 0.65 | 0.085 | 0.763 |
| HaPYL2b | P2C06 | 4232.A0A251UAP0 | 4232.A0A251TU99 | 0 | 0.68 | 0.65 | 0.262 | 0.91 |
| HaPYL2b | HAB1 | 4232.A0A251UAP0 | 4232.A0A251U1I6 | 0 | 0.68 | 0.65 | 0.262 | 0.91 |
| HaPYL2b | A0A251UNA0 | 4232.A0A251UAP0 | 4232.A0A251UNA0 | 0 | 0.27 | 0.65 | 0.108 | 0.752 |
| HaPYL2b | A0A251UCZ1 | 4232.A0A251UAP0 | 4232.A0A251UCZ1 | 0 | 0.322 | 0.65 | 0.085 | 0.763 |
| HaPYL2b | A0A251UEA5 | 4232.A0A251UAP0 | 4232.A0A251UEA5 | 0 | 0.322 | 0.65 | 0.085 | 0.763 |
| HaPYL2b | A0A251UCX7 | 4232.A0A251UAP0 | 4232.A0A251UCX7 | 0 | 0.322 | 0.65 | 0.085 | 0.763 |
| HaPYL2b | A0A251UJ72 | 4232.A0A251UAP0 | 4232.A0A251UJ72 | 0 | 0.322 | 0.65 | 0.085 | 0.763 |
| HaPYL2b | A0A251UN79 | 4232.A0A251UAP0 | 4232.A0A251UN79 | 0 | 0.322 | 0.65 | 0.085 | 0.763 |
| A0A251UCX7 | HaPYL8c | 4232.A0A251UCX7 | 4232.A0A251RLE6 | 0 | 0.203 | 0.65 | 0.08 | 0.72 |
| A0A251UCX7 | HaPYL4b | 4232.A0A251UCX7 | 4232.A0A251RSA8 | 0 | 0.259 | 0.65 | 0.08 | 0.74 |
| A0A251UCX7 | OST1 | 4232.A0A251UCX7 | 4232.A0A251RWW9 | 0 | 0.144 | 0.665 | 0.153 | 0.736 |
| A0A251UCX7 | SPK-2-2 | 4232.A0A251UCX7 | 4232.A0A251SEN3 | 0 | 0.144 | 0.665 | 0.153 | 0.736 |
| A0A251UCX7 | ASK2 | 4232.A0A251UCX7 | 4232.A0A251TBT3 | 0 | 0.144 | 0.665 | 0.176 | 0.743 |
| A0A251UCX7 | SNRK2-10 | 4232.A0A251UCX7 | 4232.A0A251TJT8 | 0 | 0.144 | 0.665 | 0.176 | 0.743 |
| A0A251UCX7 | MPK4 | 4232.A0A251UCX7 | 4232.A0A251TTG0 | 0.06 | 0.192 | 0.081 | 0.654 | 0.726 |
| A0A251UCX7 | HaPYL9a | 4232.A0A251UCX7 | 4232.A0A251V2E7 | 0 | 0.203 | 0.65 | 0.08 | 0.72 |
| A0A251UCX7 | HaPYL8a | 4232.A0A251UCX7 | 4232.A0A251UGS1 | 0 | 0.203 | 0.65 | 0.08 | 0.72 |
| A0A251UCX7 | HaPYL9b | 4232.A0A251UCX7 | 4232.A0A251UGY0 | 0 | 0.203 | 0.65 | 0.08 | 0.72 |
| A0A251UCX7 | HaPYL8b | 4232.A0A251UCX7 | 4232.A0A251UE63 | 0 | 0.203 | 0.65 | 0.08 | 0.72 |
| A0A251UCX7 | A0A251V6D2 | 4232.A0A251UCX7 | 4232.A0A251V6D2 | 0 | 0.144 | 0.665 | 0.153 | 0.736 |
| A0A251UCX7 | HaPYL4c | 4232.A0A251UCX7 | 4232.A0A251UDF9 | 0 | 0.259 | 0.65 | 0.08 | 0.74 |
| A0A251UCX7 | A0A251UJ18 | 4232.A0A251UCX7 | 4232.A0A251UJ18 | 0 | 0.144 | 0.665 | 0.176 | 0.743 |
| A0A251UCX7 | SAPK3 | 4232.A0A251UCX7 | 4232.A0A251VNN9 | 0 | 0.144 | 0.665 | 0.176 | 0.743 |
| A0A251UCX7 | A0A251VAR3 | 4232.A0A251UCX7 | 4232.A0A251VAR3 | 0 | 0.144 | 0.665 | 0.176 | 0.743 |
| A0A251UCX7 | HaPYL2a | 4232.A0A251UCX7 | 4232.A0A251V933 | 0 | 0.322 | 0.65 | 0.085 | 0.763 |
| A0A251UCX7 | HaPYR1a | 4232.A0A251UCX7 | 4232.A0A251UG74 | 0 | 0.395 | 0.65 | 0.146 | 0.803 |
| A0A251UCZ1 | HaPYL8c | 4232.A0A251UCZ1 | 4232.A0A251RLE6 | 0 | 0.203 | 0.65 | 0.08 | 0.72 |
| A0A251UCZ1 | HaPYL4b | 4232.A0A251UCZ1 | 4232.A0A251RSA8 | 0 | 0.259 | 0.65 | 0.08 | 0.74 |
| A0A251UCZ1 | OST1 | 4232.A0A251UCZ1 | 4232.A0A251RWW9 | 0 | 0.144 | 0.665 | 0.153 | 0.736 |
| A0A251UCZ1 | SPK-2-2 | 4232.A0A251UCZ1 | 4232.A0A251SEN3 | 0 | 0.144 | 0.665 | 0.153 | 0.736 |
| A0A251UCZ1 | ASK2 | 4232.A0A251UCZ1 | 4232.A0A251TBT3 | 0 | 0.144 | 0.665 | 0.176 | 0.743 |
| A0A251UCZ1 | SNRK2-10 | 4232.A0A251UCZ1 | 4232.A0A251TJT8 | 0 | 0.144 | 0.665 | 0.176 | 0.743 |
| A0A251UCZ1 | MPK4 | 4232.A0A251UCZ1 | 4232.A0A251TTG0 | 0.06 | 0.192 | 0.081 | 0.654 | 0.726 |
| A0A251UCZ1 | HaPYL9b | 4232.A0A251UCZ1 | 4232.A0A251UGY0 | 0 | 0.203 | 0.65 | 0.08 | 0.72 |
| A0A251UCZ1 | HaPYL8b | 4232.A0A251UCZ1 | 4232.A0A251UE63 | 0 | 0.203 | 0.65 | 0.08 | 0.72 |
| A0A251UCZ1 | HaPYL9a | 4232.A0A251UCZ1 | 4232.A0A251V2E7 | 0 | 0.203 | 0.65 | 0.08 | 0.72 |
| A0A251UCZ1 | HaPYL8a | 4232.A0A251UCZ1 | 4232.A0A251UGS1 | 0 | 0.203 | 0.65 | 0.08 | 0.72 |
| A0A251UCZ1 | A0A251V6D2 | 4232.A0A251UCZ1 | 4232.A0A251V6D2 | 0 | 0.144 | 0.665 | 0.153 | 0.736 |
| A0A251UCZ1 | HaPYL4c | 4232.A0A251UCZ1 | 4232.A0A251UDF9 | 0 | 0.259 | 0.65 | 0.08 | 0.74 |
| A0A251UCZ1 | A0A251VAR3 | 4232.A0A251UCZ1 | 4232.A0A251VAR3 | 0 | 0.144 | 0.665 | 0.176 | 0.743 |
| A0A251UCZ1 | SAPK3 | 4232.A0A251UCZ1 | 4232.A0A251VNN9 | 0 | 0.144 | 0.665 | 0.176 | 0.743 |
| A0A251UCZ1 | A0A251UJ18 | 4232.A0A251UCZ1 | 4232.A0A251UJ18 | 0 | 0.144 | 0.665 | 0.176 | 0.743 |
| A0A251UCZ1 | HaPYL2a | 4232.A0A251UCZ1 | 4232.A0A251V933 | 0 | 0.322 | 0.65 | 0.085 | 0.763 |
| A0A251UCZ1 | HaPYR1a | 4232.A0A251UCZ1 | 4232.A0A251UG74 | 0 | 0.395 | 0.65 | 0.146 | 0.803 |
| A0A251UEA5 | HaPYL8c | 4232.A0A251UEA5 | 4232.A0A251RLE6 | 0 | 0.203 | 0.65 | 0.08 | 0.72 |
| A0A251UEA5 | HaPYL4b | 4232.A0A251UEA5 | 4232.A0A251RSA8 | 0 | 0.259 | 0.65 | 0.08 | 0.74 |
| A0A251UEA5 | OST1 | 4232.A0A251UEA5 | 4232.A0A251RWW9 | 0 | 0.144 | 0.665 | 0.153 | 0.736 |
| A0A251UEA5 | SPK-2-2 | 4232.A0A251UEA5 | 4232.A0A251SEN3 | 0 | 0.144 | 0.665 | 0.153 | 0.736 |
| A0A251UEA5 | ASK2 | 4232.A0A251UEA5 | 4232.A0A251TBT3 | 0 | 0.144 | 0.665 | 0.176 | 0.743 |
| A0A251UEA5 | SNRK2-10 | 4232.A0A251UEA5 | 4232.A0A251TJT8 | 0 | 0.144 | 0.665 | 0.176 | 0.743 |
| A0A251UEA5 | MPK4 | 4232.A0A251UEA5 | 4232.A0A251TTG0 | 0.06 | 0.192 | 0.081 | 0.654 | 0.726 |
| A0A251UEA5 | HaPYL4c | 4232.A0A251UEA5 | 4232.A0A251UDF9 | 0 | 0.259 | 0.65 | 0.08 | 0.74 |
| A0A251UEA5 | HaPYL8b | 4232.A0A251UEA5 | 4232.A0A251UE63 | 0 | 0.203 | 0.65 | 0.08 | 0.72 |
| A0A251UEA5 | HaPYL9b | 4232.A0A251UEA5 | 4232.A0A251UGY0 | 0 | 0.203 | 0.65 | 0.08 | 0.72 |
| A0A251UEA5 | HaPYL8a | 4232.A0A251UEA5 | 4232.A0A251UGS1 | 0 | 0.203 | 0.65 | 0.08 | 0.72 |
| A0A251UEA5 | HaPYL9a | 4232.A0A251UEA5 | 4232.A0A251V2E7 | 0 | 0.203 | 0.65 | 0.08 | 0.72 |
| A0A251UEA5 | A0A251V6D2 | 4232.A0A251UEA5 | 4232.A0A251V6D2 | 0 | 0.144 | 0.665 | 0.153 | 0.736 |
| A0A251UEA5 | A0A251UJ18 | 4232.A0A251UEA5 | 4232.A0A251UJ18 | 0 | 0.144 | 0.665 | 0.176 | 0.743 |
| A0A251UEA5 | A0A251VAR3 | 4232.A0A251UEA5 | 4232.A0A251VAR3 | 0 | 0.144 | 0.665 | 0.176 | 0.743 |
| A0A251UEA5 | SAPK3 | 4232.A0A251UEA5 | 4232.A0A251VNN9 | 0 | 0.144 | 0.665 | 0.176 | 0.743 |
| A0A251UEA5 | HaPYL2a | 4232.A0A251UEA5 | 4232.A0A251V933 | 0 | 0.322 | 0.65 | 0.085 | 0.763 |
| A0A251UEA5 | HaPYR1a | 4232.A0A251UEA5 | 4232.A0A251UG74 | 0 | 0.395 | 0.65 | 0.146 | 0.803 |
| HaPYR1a | HAI2 | 4232.A0A251UG74 | 4232.A0A251SSF2 | 0 | 0.105 | 0.65 | 0.222 | 0.735 |
| HaPYR1a | ABI2 | 4232.A0A251UG74 | 4232.A0A251TQT8 | 0 | 0.105 | 0.65 | 0.191 | 0.724 |
| HaPYR1a | ATPP2CA | 4232.A0A251UG74 | 4232.A0A251TTP8 | 0 | 0.395 | 0.65 | 0.146 | 0.803 |
| HaPYR1a | P2C06 | 4232.A0A251UG74 | 4232.A0A251TU99 | 0 | 0.477 | 0.65 | 0.311 | 0.862 |
| HaPYR1a | HAB1 | 4232.A0A251UG74 | 4232.A0A251U1I6 | 0 | 0.477 | 0.65 | 0.311 | 0.862 |
| HaPYR1a | A0A251UNA0 | 4232.A0A251UG74 | 4232.A0A251UNA0 | 0 | 0.105 | 0.65 | 0.222 | 0.735 |
| HaPYR1a | A0A251UN79 | 4232.A0A251UG74 | 4232.A0A251UN79 | 0 | 0.395 | 0.65 | 0.146 | 0.803 |
| HaPYR1a | A0A251UJ72 | 4232.A0A251UG74 | 4232.A0A251UJ72 | 0 | 0.395 | 0.65 | 0.146 | 0.803 |
| HaPYL9b | HAI2 | 4232.A0A251UGY0 | 4232.A0A251SSF2 | 0 | 0.203 | 0.65 | 0.153 | 0.743 |
| HaPYL9b | ABI2 | 4232.A0A251UGY0 | 4232.A0A251TQT8 | 0 | 0.203 | 0.65 | 0 | 0.709 |
| HaPYL9b | ATPP2CA | 4232.A0A251UGY0 | 4232.A0A251TTP8 | 0 | 0.203 | 0.65 | 0.08 | 0.72 |
| HaPYL9b | P2C06 | 4232.A0A251UGY0 | 4232.A0A251TU99 | 0 | 0.621 | 0.65 | 0.181 | 0.881 |
| HaPYL9b | HAB1 | 4232.A0A251UGY0 | 4232.A0A251U1I6 | 0 | 0.621 | 0.65 | 0.181 | 0.881 |
| HaPYL9b | A0A251UN79 | 4232.A0A251UGY0 | 4232.A0A251UN79 | 0 | 0.203 | 0.65 | 0.08 | 0.72 |
| HaPYL9b | A0A251UJ72 | 4232.A0A251UGY0 | 4232.A0A251UJ72 | 0 | 0.203 | 0.65 | 0.08 | 0.72 |
| HaPYL9b | A0A251UNA0 | 4232.A0A251UGY0 | 4232.A0A251UNA0 | 0 | 0.203 | 0.65 | 0.153 | 0.743 |
| A0A251UJ18 | HAI2 | 4232.A0A251UJ18 | 4232.A0A251SSF2 | 0 | 0.144 | 0.665 | 0.202 | 0.751 |
| A0A251UJ18 | ABI2 | 4232.A0A251UJ18 | 4232.A0A251TQT8 | 0 | 0.144 | 0.665 | 0.222 | 0.757 |
| A0A251UJ18 | ATPP2CA | 4232.A0A251UJ18 | 4232.A0A251TTP8 | 0 | 0.144 | 0.665 | 0.176 | 0.743 |
| A0A251UJ18 | P2C06 | 4232.A0A251UJ18 | 4232.A0A251TU99 | 0 | 0.273 | 0.665 | 0.208 | 0.79 |
| A0A251UJ18 | HAB1 | 4232.A0A251UJ18 | 4232.A0A251U1I6 | 0 | 0.273 | 0.665 | 0.208 | 0.79 |
| A0A251UJ18 | A0A251UJ72 | 4232.A0A251UJ18 | 4232.A0A251UJ72 | 0 | 0.144 | 0.665 | 0.176 | 0.743 |
| A0A251UJ18 | A0A251UN79 | 4232.A0A251UJ18 | 4232.A0A251UN79 | 0 | 0.144 | 0.665 | 0.176 | 0.743 |
| A0A251UJ18 | A0A251UNA0 | 4232.A0A251UJ18 | 4232.A0A251UNA0 | 0 | 0.144 | 0.665 | 0.202 | 0.751 |
| A0A251UJ72 | HaPYL8c | 4232.A0A251UJ72 | 4232.A0A251RLE6 | 0 | 0.203 | 0.65 | 0.08 | 0.72 |
| A0A251UJ72 | HaPYL4b | 4232.A0A251UJ72 | 4232.A0A251RSA8 | 0 | 0.259 | 0.65 | 0.08 | 0.74 |
| A0A251UJ72 | OST1 | 4232.A0A251UJ72 | 4232.A0A251RWW9 | 0 | 0.144 | 0.665 | 0.153 | 0.736 |
| A0A251UJ72 | SPK-2-2 | 4232.A0A251UJ72 | 4232.A0A251SEN3 | 0 | 0.144 | 0.665 | 0.153 | 0.736 |
| A0A251UJ72 | ASK2 | 4232.A0A251UJ72 | 4232.A0A251TBT3 | 0 | 0.144 | 0.665 | 0.176 | 0.743 |
| A0A251UJ72 | SNRK2-10 | 4232.A0A251UJ72 | 4232.A0A251TJT8 | 0 | 0.144 | 0.665 | 0.176 | 0.743 |
| A0A251UJ72 | MPK4 | 4232.A0A251UJ72 | 4232.A0A251TTG0 | 0.06 | 0.192 | 0.081 | 0.654 | 0.726 |
| A0A251UJ72 | HaPYL4c | 4232.A0A251UJ72 | 4232.A0A251UDF9 | 0 | 0.259 | 0.65 | 0.08 | 0.74 |
| A0A251UJ72 | HaPYL8b | 4232.A0A251UJ72 | 4232.A0A251UE63 | 0 | 0.203 | 0.65 | 0.08 | 0.72 |
| A0A251UJ72 | HaPYL8a | 4232.A0A251UJ72 | 4232.A0A251UGS1 | 0 | 0.203 | 0.65 | 0.08 | 0.72 |
| A0A251UJ72 | HaPYL9a | 4232.A0A251UJ72 | 4232.A0A251V2E7 | 0 | 0.203 | 0.65 | 0.08 | 0.72 |
| A0A251UJ72 | A0A251V6D2 | 4232.A0A251UJ72 | 4232.A0A251V6D2 | 0 | 0.144 | 0.665 | 0.153 | 0.736 |
| A0A251UJ72 | SAPK3 | 4232.A0A251UJ72 | 4232.A0A251VNN9 | 0 | 0.144 | 0.665 | 0.176 | 0.743 |
| A0A251UJ72 | A0A251VAR3 | 4232.A0A251UJ72 | 4232.A0A251VAR3 | 0 | 0.144 | 0.665 | 0.176 | 0.743 |
| A0A251UJ72 | HaPYL2a | 4232.A0A251UJ72 | 4232.A0A251V933 | 0 | 0.322 | 0.65 | 0.085 | 0.763 |
| A0A251UN79 | HaPYL8c | 4232.A0A251UN79 | 4232.A0A251RLE6 | 0 | 0.203 | 0.65 | 0.08 | 0.72 |
| A0A251UN79 | HaPYL4b | 4232.A0A251UN79 | 4232.A0A251RSA8 | 0 | 0.259 | 0.65 | 0.08 | 0.74 |
| A0A251UN79 | OST1 | 4232.A0A251UN79 | 4232.A0A251RWW9 | 0 | 0.144 | 0.665 | 0.153 | 0.736 |
| A0A251UN79 | SPK-2-2 | 4232.A0A251UN79 | 4232.A0A251SEN3 | 0 | 0.144 | 0.665 | 0.153 | 0.736 |
| A0A251UN79 | ASK2 | 4232.A0A251UN79 | 4232.A0A251TBT3 | 0 | 0.144 | 0.665 | 0.176 | 0.743 |
| A0A251UN79 | SNRK2-10 | 4232.A0A251UN79 | 4232.A0A251TJT8 | 0 | 0.144 | 0.665 | 0.176 | 0.743 |
| A0A251UN79 | MPK4 | 4232.A0A251UN79 | 4232.A0A251TTG0 | 0.06 | 0.192 | 0.081 | 0.654 | 0.726 |
| A0A251UN79 | HaPYL4c | 4232.A0A251UN79 | 4232.A0A251UDF9 | 0 | 0.259 | 0.65 | 0.08 | 0.74 |
| A0A251UN79 | HaPYL8b | 4232.A0A251UN79 | 4232.A0A251UE63 | 0 | 0.203 | 0.65 | 0.08 | 0.72 |
| A0A251UN79 | HaPYL8a | 4232.A0A251UN79 | 4232.A0A251UGS1 | 0 | 0.203 | 0.65 | 0.08 | 0.72 |
| A0A251UN79 | HaPYL9a | 4232.A0A251UN79 | 4232.A0A251V2E7 | 0 | 0.203 | 0.65 | 0.08 | 0.72 |
| A0A251UN79 | A0A251V6D2 | 4232.A0A251UN79 | 4232.A0A251V6D2 | 0 | 0.144 | 0.665 | 0.153 | 0.736 |
| A0A251UN79 | A0A251VAR3 | 4232.A0A251UN79 | 4232.A0A251VAR3 | 0 | 0.144 | 0.665 | 0.176 | 0.743 |
| A0A251UN79 | SAPK3 | 4232.A0A251UN79 | 4232.A0A251VNN9 | 0 | 0.144 | 0.665 | 0.176 | 0.743 |
| A0A251UN79 | HaPYL2a | 4232.A0A251UN79 | 4232.A0A251V933 | 0 | 0.322 | 0.65 | 0.085 | 0.763 |
| A0A251UNA0 | HaPYL8c | 4232.A0A251UNA0 | 4232.A0A251RLE6 | 0 | 0.203 | 0.65 | 0.153 | 0.743 |
| A0A251UNA0 | OST1 | 4232.A0A251UNA0 | 4232.A0A251RWW9 | 0 | 0.144 | 0.665 | 0.251 | 0.766 |
| A0A251UNA0 | SPK-2-2 | 4232.A0A251UNA0 | 4232.A0A251SEN3 | 0 | 0.144 | 0.665 | 0.251 | 0.766 |
| A0A251UNA0 | ASK2 | 4232.A0A251UNA0 | 4232.A0A251TBT3 | 0 | 0.144 | 0.665 | 0.202 | 0.751 |
| A0A251UNA0 | SNRK2-10 | 4232.A0A251UNA0 | 4232.A0A251TJT8 | 0 | 0.144 | 0.665 | 0.202 | 0.751 |
| A0A251UNA0 | MPK4 | 4232.A0A251UNA0 | 4232.A0A251TTG0 | 0.06 | 0.192 | 0.081 | 0.654 | 0.726 |
| A0A251UNA0 | HaPYL8b | 4232.A0A251UNA0 | 4232.A0A251UE63 | 0 | 0.203 | 0.65 | 0.153 | 0.743 |
| A0A251UNA0 | HaPYL8a | 4232.A0A251UNA0 | 4232.A0A251UGS1 | 0 | 0.203 | 0.65 | 0.153 | 0.743 |
| A0A251UNA0 | HaPYL9a | 4232.A0A251UNA0 | 4232.A0A251V2E7 | 0 | 0.203 | 0.65 | 0.153 | 0.743 |
| A0A251UNA0 | HaPYL2a | 4232.A0A251UNA0 | 4232.A0A251V933 | 0 | 0.27 | 0.65 | 0.108 | 0.752 |
| A0A251UNA0 | A0A251V6D2 | 4232.A0A251UNA0 | 4232.A0A251V6D2 | 0 | 0.144 | 0.665 | 0.251 | 0.766 |
| A0A251UNA0 | SAPK3 | 4232.A0A251UNA0 | 4232.A0A251VNN9 | 0 | 0.144 | 0.665 | 0.364 | 0.802 |
| A0A251UNA0 | A0A251VAR3 | 4232.A0A251UNA0 | 4232.A0A251VAR3 | 0 | 0.144 | 0.665 | 0.364 | 0.802 |
| HaPYL9a | HAI2 | 4232.A0A251V2E7 | 4232.A0A251SSF2 | 0 | 0.203 | 0.65 | 0.153 | 0.743 |
| HaPYL9a | ABI2 | 4232.A0A251V2E7 | 4232.A0A251TQT8 | 0 | 0.203 | 0.65 | 0 | 0.709 |
| HaPYL9a | ATPP2CA | 4232.A0A251V2E7 | 4232.A0A251TTP8 | 0 | 0.203 | 0.65 | 0.08 | 0.72 |
| HaPYL9a | P2C06 | 4232.A0A251V2E7 | 4232.A0A251TU99 | 0 | 0.621 | 0.65 | 0.181 | 0.881 |
| HaPYL9a | HAB1 | 4232.A0A251V2E7 | 4232.A0A251U1I6 | 0 | 0.621 | 0.65 | 0.181 | 0.881 |
| A0A251V6D2 | HAI2 | 4232.A0A251V6D2 | 4232.A0A251SSF2 | 0 | 0.144 | 0.665 | 0.251 | 0.766 |
| A0A251V6D2 | ABI2 | 4232.A0A251V6D2 | 4232.A0A251TQT8 | 0 | 0.144 | 0.665 | 0.302 | 0.782 |
| A0A251V6D2 | ATPP2CA | 4232.A0A251V6D2 | 4232.A0A251TTP8 | 0 | 0.144 | 0.665 | 0.153 | 0.736 |
| A0A251V6D2 | P2C06 | 4232.A0A251V6D2 | 4232.A0A251TU99 | 0 | 0.623 | 0.665 | 0.252 | 0.897 |
| A0A251V6D2 | HAB1 | 4232.A0A251V6D2 | 4232.A0A251U1I6 | 0 | 0.623 | 0.665 | 0.252 | 0.897 |
| A0A251VAR3 | HAI2 | 4232.A0A251VAR3 | 4232.A0A251SSF2 | 0 | 0.144 | 0.665 | 0.364 | 0.802 |
| A0A251VAR3 | ABI2 | 4232.A0A251VAR3 | 4232.A0A251TQT8 | 0 | 0.144 | 0.665 | 0.373 | 0.804 |
| A0A251VAR3 | ATPP2CA | 4232.A0A251VAR3 | 4232.A0A251TTP8 | 0 | 0.144 | 0.665 | 0.176 | 0.743 |
| A0A251VAR3 | P2C06 | 4232.A0A251VAR3 | 4232.A0A251TU99 | 0 | 0.394 | 0.665 | 0.308 | 0.847 |
| A0A251VAR3 | HAB1 | 4232.A0A251VAR3 | 4232.A0A251U1I6 | 0 | 0.394 | 0.665 | 0.308 | 0.847 |
| ABI2 | HaPYL8c | 4232.A0A251TQT8 | 4232.A0A251RLE6 | 0 | 0.203 | 0.65 | 0 | 0.709 |
| ABI2 | OST1 | 4232.A0A251TQT8 | 4232.A0A251RWW9 | 0 | 0.144 | 0.665 | 0.302 | 0.782 |
| ABI2 | SPK-2-2 | 4232.A0A251TQT8 | 4232.A0A251SEN3 | 0 | 0.144 | 0.665 | 0.302 | 0.782 |
| ABI2 | ASK2 | 4232.A0A251TQT8 | 4232.A0A251TBT3 | 0 | 0.144 | 0.665 | 0.222 | 0.757 |
| ABI2 | SNRK2-10 | 4232.A0A251TQT8 | 4232.A0A251TJT8 | 0 | 0.144 | 0.665 | 0.222 | 0.757 |
| ABI2 | MPK4 | 4232.A0A251TQT8 | 4232.A0A251TTG0 | 0.06 | 0.192 | 0.081 | 0.632 | 0.709 |
| ABI2 | SOS2 | 4232.A0A251TQT8 | 4232.A0A251UKH9 | 0 | 0.144 | 0.084 | 0.691 | 0.736 |
| ABI2 | SAPK3 | 4232.A0A251TQT8 | 4232.A0A251VNN9 | 0 | 0.144 | 0.665 | 0.373 | 0.804 |
| ABI2 | HaPYL8b | 4232.A0A251TQT8 | 4232.A0A251UE63 | 0 | 0.203 | 0.65 | 0.461 | 0.836 |
| ABI2 | HaPYL8a | 4232.A0A251TQT8 | 4232.A0A251UGS1 | 0 | 0.203 | 0.65 | 0.496 | 0.847 |
| ABI2 | HaPYL2a | 4232.A0A251TQT8 | 4232.A0A251V933 | 0 | 0.27 | 0.65 | 0.494 | 0.859 |
| ASK2 | HAI2 | 4232.A0A251TBT3 | 4232.A0A251SSF2 | 0 | 0.144 | 0.665 | 0.202 | 0.751 |
| ASK2 | ATPP2CA | 4232.A0A251TBT3 | 4232.A0A251TTP8 | 0 | 0.144 | 0.665 | 0.176 | 0.743 |
| ASK2 | HAB1 | 4232.A0A251TBT3 | 4232.A0A251U1I6 | 0 | 0.273 | 0.665 | 0.208 | 0.79 |
| ASK2 | P2C06 | 4232.A0A251TBT3 | 4232.A0A251TU99 | 0 | 0.273 | 0.665 | 0.208 | 0.79 |
| ATPP2CA | HaPYL8c | 4232.A0A251TTP8 | 4232.A0A251RLE6 | 0 | 0.203 | 0.65 | 0.08 | 0.72 |
| ATPP2CA | HaPYL4b | 4232.A0A251TTP8 | 4232.A0A251RSA8 | 0 | 0.259 | 0.65 | 0.08 | 0.74 |
| ATPP2CA | OST1 | 4232.A0A251TTP8 | 4232.A0A251RWW9 | 0 | 0.144 | 0.665 | 0.153 | 0.736 |
| ATPP2CA | SPK-2-2 | 4232.A0A251TTP8 | 4232.A0A251SEN3 | 0 | 0.144 | 0.665 | 0.153 | 0.736 |
| ATPP2CA | SNRK2-10 | 4232.A0A251TTP8 | 4232.A0A251TJT8 | 0 | 0.144 | 0.665 | 0.176 | 0.743 |
| ATPP2CA | MPK4 | 4232.A0A251TTP8 | 4232.A0A251TTG0 | 0.06 | 0.192 | 0.081 | 0.654 | 0.726 |
| ATPP2CA | HaPYL8a | 4232.A0A251TTP8 | 4232.A0A251UGS1 | 0 | 0.203 | 0.65 | 0.08 | 0.72 |
| ATPP2CA | HaPYL8b | 4232.A0A251TTP8 | 4232.A0A251UE63 | 0 | 0.203 | 0.65 | 0.08 | 0.72 |
| ATPP2CA | HaPYL4c | 4232.A0A251TTP8 | 4232.A0A251UDF9 | 0 | 0.259 | 0.65 | 0.08 | 0.74 |
| ATPP2CA | SAPK3 | 4232.A0A251TTP8 | 4232.A0A251VNN9 | 0 | 0.144 | 0.665 | 0.176 | 0.743 |
| ATPP2CA | HaPYL2a | 4232.A0A251TTP8 | 4232.A0A251V933 | 0 | 0.322 | 0.65 | 0.085 | 0.763 |
| CYP707A1 | HaPYL2a | 4232.A0A251UHP7 | 4232.A0A251V933 | 0 | 0 | 0 | 0.701 | 0.701 |
| HAB1 | HaPYL8c | 4232.A0A251U1I6 | 4232.A0A251RLE6 | 0 | 0.621 | 0.65 | 0.181 | 0.881 |
| HAB1 | HaPYL4b | 4232.A0A251U1I6 | 4232.A0A251RSA8 | 0 | 0.273 | 0.65 | 0.181 | 0.773 |
| HAB1 | OST1 | 4232.A0A251U1I6 | 4232.A0A251RWW9 | 0 | 0.623 | 0.665 | 0.252 | 0.897 |
| HAB1 | SPK-2-2 | 4232.A0A251U1I6 | 4232.A0A251SEN3 | 0 | 0.623 | 0.665 | 0.252 | 0.897 |
| HAB1 | SNRK2-10 | 4232.A0A251U1I6 | 4232.A0A251TJT8 | 0 | 0.273 | 0.665 | 0.208 | 0.79 |
| HAB1 | MPK4 | 4232.A0A251U1I6 | 4232.A0A251TTG0 | 0.06 | 0.192 | 0.081 | 0.654 | 0.726 |
| HAB1 | HaPYL4c | 4232.A0A251U1I6 | 4232.A0A251UDF9 | 0 | 0.273 | 0.65 | 0.181 | 0.773 |
| HAB1 | SAPK3 | 4232.A0A251U1I6 | 4232.A0A251VNN9 | 0 | 0.394 | 0.665 | 0.308 | 0.847 |
| HAB1 | HaPYL8a | 4232.A0A251U1I6 | 4232.A0A251UGS1 | 0 | 0.621 | 0.65 | 0.181 | 0.881 |
| HAB1 | HaPYL8b | 4232.A0A251U1I6 | 4232.A0A251UE63 | 0 | 0.621 | 0.65 | 0.181 | 0.881 |
| HAB1 | HaPYL2a | 4232.A0A251U1I6 | 4232.A0A251V933 | 0 | 0.68 | 0.65 | 0.262 | 0.91 |
| HAI2 | HaPYL8c | 4232.A0A251SSF2 | 4232.A0A251RLE6 | 0 | 0.203 | 0.65 | 0.153 | 0.743 |
| HAI2 | OST1 | 4232.A0A251SSF2 | 4232.A0A251RWW9 | 0 | 0.144 | 0.665 | 0.251 | 0.766 |
| HAI2 | SPK-2-2 | 4232.A0A251SSF2 | 4232.A0A251SEN3 | 0 | 0.144 | 0.665 | 0.251 | 0.766 |
| HAI2 | MPK4 | 4232.A0A251SSF2 | 4232.A0A251TTG0 | 0.06 | 0.192 | 0.081 | 0.651 | 0.724 |
| HAI2 | HaPYL8b | 4232.A0A251SSF2 | 4232.A0A251UE63 | 0 | 0.203 | 0.65 | 0.153 | 0.743 |
| HAI2 | HaPYL8a | 4232.A0A251SSF2 | 4232.A0A251UGS1 | 0 | 0.203 | 0.65 | 0.153 | 0.743 |
| HAI2 | SNRK2-10 | 4232.A0A251SSF2 | 4232.A0A251TJT8 | 0 | 0.144 | 0.665 | 0.202 | 0.751 |
| HAI2 | HaPYL2a | 4232.A0A251SSF2 | 4232.A0A251V933 | 0 | 0.27 | 0.65 | 0.108 | 0.752 |
| HAI2 | SAPK3 | 4232.A0A251SSF2 | 4232.A0A251VNN9 | 0 | 0.144 | 0.665 | 0.364 | 0.802 |
| MPK4 | P2C06 | 4232.A0A251TTG0 | 4232.A0A251TU99 | 0.06 | 0.192 | 0.081 | 0.654 | 0.726 |
| OST1 | P2C06 | 4232.A0A251RWW9 | 4232.A0A251TU99 | 0 | 0.623 | 0.665 | 0.252 | 0.897 |
| P2C06 | HaPYL8c | 4232.A0A251TU99 | 4232.A0A251RLE6 | 0 | 0.621 | 0.65 | 0.181 | 0.881 |
| P2C06 | HaPYL4b | 4232.A0A251TU99 | 4232.A0A251RSA8 | 0 | 0.273 | 0.65 | 0.181 | 0.773 |
| P2C06 | SPK-2-2 | 4232.A0A251TU99 | 4232.A0A251SEN3 | 0 | 0.623 | 0.665 | 0.252 | 0.897 |
| P2C06 | SNRK2-10 | 4232.A0A251TU99 | 4232.A0A251TJT8 | 0 | 0.273 | 0.665 | 0.208 | 0.79 |
| P2C06 | HaPYL4c | 4232.A0A251TU99 | 4232.A0A251UDF9 | 0 | 0.273 | 0.65 | 0.181 | 0.773 |
| P2C06 | SAPK3 | 4232.A0A251TU99 | 4232.A0A251VNN9 | 0 | 0.394 | 0.665 | 0.308 | 0.847 |
| P2C06 | HaPYL8a | 4232.A0A251TU99 | 4232.A0A251UGS1 | 0 | 0.621 | 0.65 | 0.181 | 0.881 |
| P2C06 | HaPYL8b | 4232.A0A251TU99 | 4232.A0A251UE63 | 0 | 0.621 | 0.65 | 0.181 | 0.881 |
| P2C06 | HaPYL2a | 4232.A0A251TU99 | 4232.A0A251V933 | 0 | 0.68 | 0.65 | 0.262 | 0.91 |
| HaPYR1b | A0A251TQ41 | 4232.A0A251SZY4 | 4232.A0A251TQ41 | 0 | 0.477 | 0 | 0.311 | 0.624 |
| HaPYR1b | A0A251TSF6 | 4232.A0A251SZY4 | 4232.A0A251TSF6 | 0 | 0.477 | 0 | 0.311 | 0.624 |
| HaPYR1b | P2C06 | 4232.A0A251SZY4 | 4232.A0A251TU99 | 0 | 0.477 | 0 | 0.311 | 0.624 |
| HaPYR1b | HAB1 | 4232.A0A251SZY4 | 4232.A0A251U1I6 | 0 | 0.477 | 0 | 0.311 | 0.624 |

**Additional file 8. Protein annotation of HaPYLs interacting proteins predicted by the STRING online service.**

| STRING_ID | STRING_name | gene_name | gene_accession |
| --- | --- | --- | --- |
| A0A251TU99 | P2C06 | PP2C 6 | HanXRQr2_Chr09g0373871 |
| A0A251TTP8 | ATPP2CA | PP2C 51 | HanXRQr2_Chr09g0381581 |
| A0A251U1I6 | HAB1 | PP2C 16 | HanXRQr2_Chr09g0417071 |
| A0A251TQ41 | A0A251TQ41 | PP2C 16 | HanXRQr2_Chr10g0459251 |
| A0A251UJ72 | A0A251UJ72 | PP2C 24 | HanXRQr2_Chr06g0260851 |
| A0A251UN79 | A0A251UN79 | PP2C 24 | HanXRQr2_Chr05g0198501 |
| A0A251U0K7 | A0A251U0K7 | PP2C 24 | HanXRQr2_Chr09g0416241 |
| A0A251U9L3 | A0A251U9L3 | PP2C 24 | HanXRQr2_Chr08g0360961 |
| A0A251UCX7 | A0A251UCX7 | PP2C 37 | HanXRQr2_Chr07g0311111 |
| A0A251UCZ1 | A0A251UCZ1 | PP2C 37 | HannXRQ_Chr07g0201631, LOC110867050 |
| A0A251UEA5 | A0A251UEA5 | PP2C 37 | HanXRQr2_Chr07g0311101 |
| A0A251TP95 | A0A251TP95 | PP2C 37 | HanXRQr2_Chr14g0664951 |
| A0A251SBW4 | A0A251SBW4 | PP2C 38 | HanXRQr2_Chr15g0720051 |
| A0A251TSF6 | A0A251TSF6 | putative protein-serine/threonine phosphatase | HanXRQr2_Chr09g0367161 |
| A0A251SSF2 | HAI2 | PP2C 75 | HanXRQr2_Chr13g0591151 |
| A0A251UNA0 | A0A251UNA0 | PP2C 75 | HanXRQr2_Chr05g0210491 |
| A0A251SAZ2 | A0A251SAZ2 | PP2C 75 | HanXRQr2_Chr15g0707601 |
| A0A251TQT8 | ABI2 | PP2C 77 | HanXRQr2_Chr09g0363031 |
| A0A251UHP7 | CYP707A1 | abscisic acid 8'-hydroxylase | HanXRQr2_Chr06g0250871 |
| A0A251TJT8 | SNRK2-10 | SRK2A | HanXRQr2_Chr10g0442281 |
| A0A251TBT3 | ASK2 | SRK2A | HanXRQr2_Chr11g0504421 |
| A0A251UJ18 | A0A251UJ18 | SRK2A | HanXRQr2_Chr06g0270371 |
| A0A251SL15 | A0A251SL15 | SRK2A | HanXRQr2_Chr11g0487221 |
| A0A251S3H5 | A0A251S3H5 | SRK2A | HanXRQr2_Chr16g0750251 |
| A0A251TFU6 | A0A251TFU6 | SAPK2 | HanXRQr2_Chr10g0419071 |
| A0A251VNN9 | SAPK3 | SAPK3 | HanXRQr2_Chr06g0242431 |
| A0A251VAR3 | A0A251VAR3 | SAPK3 | HanXRQr2_Chr03g0132901 |
| A0A251SWA4 | A0A251SWA4 | SAPK3 | HanXRQr2_Chr13g0607501 |
| A0A251UKH9 | SOS2 | non-specific serine/threonine protein kinase | HanXRQr2_Chr06g0264621 |
| A0A251RWW9 | OST1 | SRK2E | HanXRQr2_Chr16g0731451 |
| A0A251V6D2 | A0A251V6D2 | SRK2E | HanXRQr2_Chr03g0110581 |
| A0A251SAY3 | A0A251SAY3 | SRK2E | HanXRQr2_Chr15g0713271 |
| A0A251SEN3 | SPK-2-2 | SRK2E, SPK-2-2 | HanXRQr2_Chr09g0398901 |
| A0A251T7F1 | A0A251T7F1 | SRK2I | HanXRQr2_Chr12g0558601 |
| A0A251TKH2 | A0A251TKH2 | Protein kinase domain-containing protein | HanXRQr2_Chr10g0443161 |
| A0A251TTG0 | MPK4 | Mitogen-activated protein kinase | HanXRQr2_Chr02g0059291 |
